# Hemoglobin modulation affects physiology and patient reported outcomes in anemic and non-anemic subjects: An umbrella review

**DOI:** 10.3389/fphys.2023.1086839

**Published:** 2023-02-15

**Authors:** R. P. B. Tonino, L. M. Zwaginga, M. R. Schipperus, J. J. Zwaginga

**Affiliations:** ^1^ Research, TRIP, Leiden, Netherlands; ^2^ Hematology, Haga Teaching Hospital, The Hague, Netherlands; ^3^ Hematology, LUMC, Leiden, Netherlands; ^4^ AUMC, Amsterdam, Netherlands; ^5^ Department of Clinical Affairs, Sanquin Bloodbank, Amsterdam, Netherlands

**Keywords:** hemoglobin, physiology, patient reported outcome measures (PROMs), red blood cell, umbrella review, oxygen transport capacity

## Abstract

**Background:** An abnormal hemoglobin concentration has a substantial effect on a person’s quality of life and physiology. Lack of tools that effectively evaluate hemoglobin-related outcomes leads to uncertainty regarding optimal hemoglobin levels, transfusion thresholds and treatment targets. We therefore aim to summarize reviews that assess the effects of hemoglobin modulation on the human physiology at various baseline hemoglobin levels, and identify gaps in existing evidence.

**Methods:** We conducted an umbrella review of systematic reviews. PubMed, MEDLINE (OVID), Embase, Web of Science, Cochrane Library and Emcare were searched from inception to the 15th of April 2022 for studies that reported on physiological and patient reported outcomes following a hemoglobin change.

**Results:** Thirty-three reviews were included of which 7 were scored as of *high* quality and 24 of *critically low* quality using the AMSTAR-2 tool. The reported data generally show that an increase in hemoglobin leads to improvement of patient reported and physical outcomes in anaemic and non-anaemic subjects. At lower hemoglobin levels, the effect of a hemoglobin modulation on quality of life measures appears more pronounced.

**Conclusion:** This overview has revealed many knowledge gaps due to a lack of high-quality evidence. For chronic kidney disease patients, a clinically relevant benefit of increasing the hemoglobin levels up until 12 g/dL was found. However, a personalized approach remains necessary due to the many patient-specific factors that affect outcomes. We strongly encourage future trials to incorporate physiological outcomes as objective parameters together with subjective, but still very important, patient reported outcome measures.

## Introduction

The main function of hemoglobin (Hb) in red blood cells (RBC) is to transport oxygen. The Hb level is biologically maintained between 12.0 and 16.0 g/dL in females, and 13.0–16.5 g/dL in males. However, an abnormal Hb level is frequently found. Globally, the prevalence of anemia (Hb < 12.0/13.0 g/dL for F/M, respectively) is estimated to be 23.2% (Safiri et al., 2021) and the prevalence of erythrocytosis (defined as Hb > 16.0/16.5 g/dL or Hematocrit (Hct) > 48%/49% for F/M, respectively) is estimated to be in the range of 0.8%–3.4%, in various populations (Ruggeri et al., 2003; Wouters et al., 2019). Both a low and a high Hb concentration may have a substantial effect on a person’s quality of life. For instance, impaired oxygen transport due to anemia may cause symptoms of fatigue and decreased physical and cognitive function (Penninx et al., 2004; Denny et al., 2006; Thein et al., 2009; Soni et al., 2010). On the other hand, erythrocytosis may result in hyperviscosity, which may cause, amongst other symptoms, shortness of breath, and physical and cognitive deterioration (McMullin et al., 2005).

For abnormal Hb-levels, often only symptomatic treatments are available: for anaemic patients, RBC transfusions, erythropoietin stimulating agents (ESAs), iron and vitamin supplementation can be utilized, amongst other therapies, typically with the goal to increase Hb levels and hence QoL, and/or decrease morbidity/mortality. Moreover, athletes may try to enhance their exercise capacity by increasing their Hb to supraphysiologic levels. For patients with erythrocytosis, on the other hand, phlebotomies and cytoreduction may be utilized to reduce hyperviscosity symptoms and the risk of thrombo-embolic complications.

The relationship between the total Hb-mass and the absolute O_2_ transport is influenced by numerous factors like age, macro-/microvascular performance and type of Hb. E.g., HbF, and HbH have a higher oxygen affinity than HbA, with HbH having an extremely high affinity, leading to tissue hypoxia even at normal Hb-levels. Contrastingly, HbS has a lower oxygen affinity compared to HbA, facilitating tissue O_2_ delivery at a lower PaO_2_. Within a single subject however, it can be assumed to some extent that this relationship between the total Hb-mass and oxygen transport is linear (Calbet et al., 2006). The lower the Hb-mass, the less O_2_ transport. Indeed, it has been reported that in patients with chronic anemia, red blood cell transfusions improve walk test distances and fatigue scores (Lezin et al., 2019). Moreover, studies showed a decrease in VO_2_max following isovolumic hemodilution and an increase in VO_2_max following RBC transfusions (Calbet et al., 2006), and studies evaluating blood donors have shown that blood donation increases the resting heart rate 24 h after donation, and decreases VO_2_max (Gordon et al., 2010). Therefore in these groups, it has been established that a change in Hb has an effect on the O_2_ transport capacity. What we do not know, however, is the magnitude of this effect at different Hb levels*.* Do these effects correlate linearly to the change in Hb level, and is there a certain level above which an increase will not exert a beneficial but even a negative effect on the patient? Does O_2_ transport decrease at a certain supraphysiological Hb level due to rheological reasons hampering microcirculatory perfusion by the more viscous blood? And if so, how do we find the patient specific Hb balancing the best possible O_2_ transport vs. microcirculatory perfusion and hyperviscosity risk?

Likewise, there is an ongoing debate about transfusion thresholds. In patients with reversible anemia restrictive transfusion strategies have become the default because of the intrinsic costs, the lack of better outcomes at higher thresholds and the small but remaining risks of blood transfusion. In contrast, in chronic deeply anaemic and transfusion dependent patients, it can be argued that a more liberal strategy might increase the QoL (Wood and McQuilten, 2020; Oliva et al., 2021). Of course, negative side-effects of Hb-modulating therapies have been established: There are well known risks in transfusion like transfusion reactions and iron overload; ESAs increase the risk of venous thromboembolism or may cause polycythemia putting the patient at risk for heart attack, stroke, or even death; and both iron and ESAs may cause tumors to grow in patients who have cancer (Salamin et al., 2018; Wilson et al., 2018). However, the balance between beneficial effects of a higher Hb level and the negative effects, costs and logistics is complex, especially if the benefits are yet unknown or challenging to quantify.

Assessing functional outcomes, like VO_2_max, exercise capacity, heart rate, and patient reported outcomes like HRQoL, fatigue and dyspnea, at various Hb levels may help to understand what effect changes in total Hb-mass have on the physiology of human beings, whether or not they are anaemic. While a reduction in Hb level from 16 to 15 g/dL may not be clinically relevant for the subject itself, documentation of its effect will help to put the puzzle of Hb optimization together. If we would have data on every shift in Hb-level in every patient group with any start off Hb level, we could predict the outcomes in patients for whom an increase or decrease in Hb level is considered. Consequently, we could make an evidenced-based decision on Hb thresholds and targets.

**FIGURE 1:**
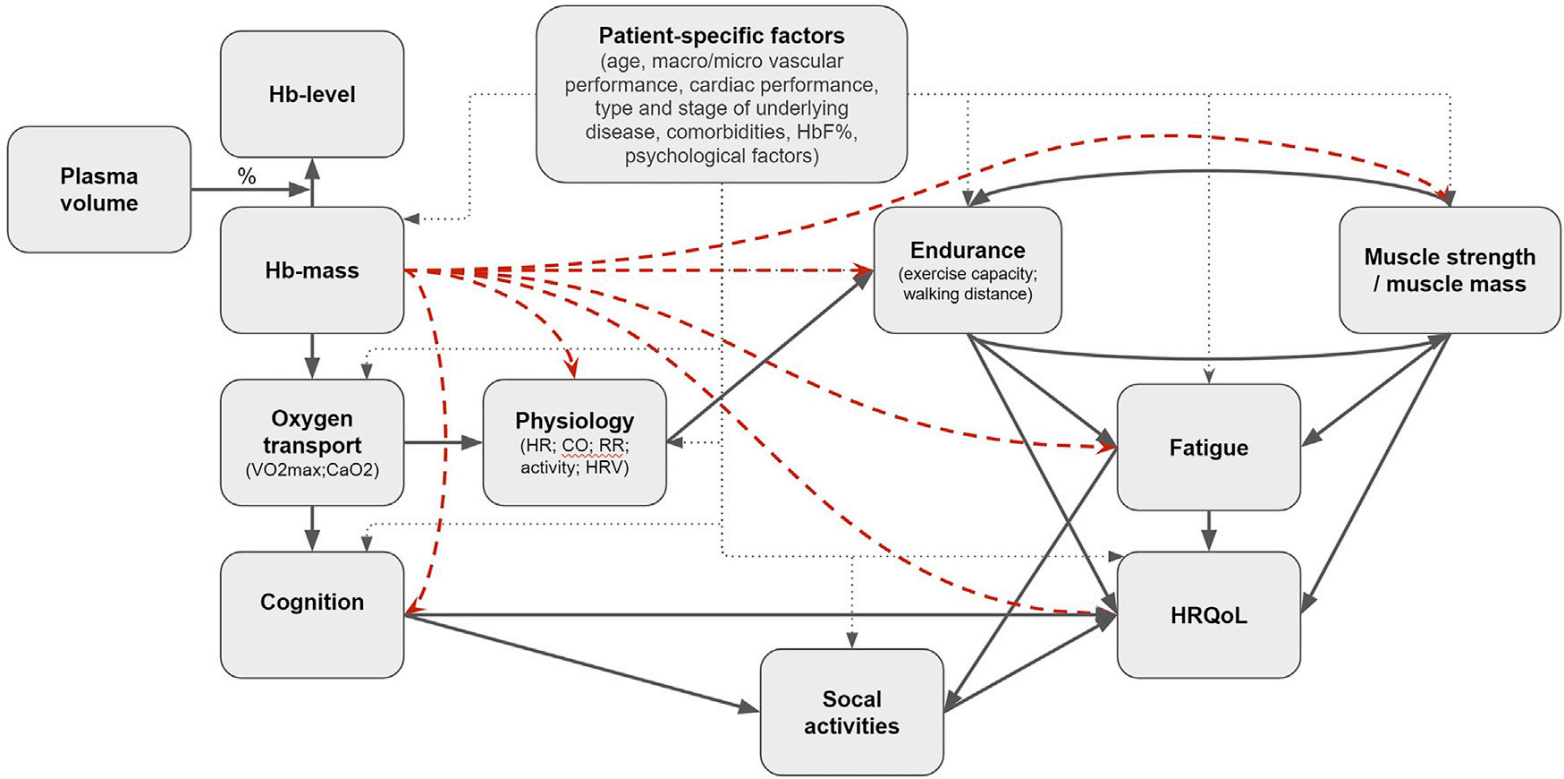
Proposed casual pathway in black arrows. The red dashed arrows are the (indirect) effects this umbrella review is intended to reflect on.

In conducting this umbrella review, we took into consideration that there are many accompanying effects of the included therapies, the disorders and the different treatments. Hence, the measured effects might not always primarily or only reflect the Hb-change. Moreover, while we do not want to compare interventions, i.e., whether ESAs are more effective than iron or transfusion, we looked per Hb modulating intervention at the hematological outcome and the consequential effect on outcomes. Due to the many patient-specific factors that affect the outcomes of Hb-levels (Figure 1), a personalized approach to the patient is always a necessity for good healthcare. Notwithstanding this, combining data from all types of interventions, patient groups and outcomes creates an overview, after which we can put the individual’s situation into perspective. Still, in a way we are comparing apples and oranges, however, this approach might tell us a great deal about our fruit—Hb modulation and the effect on outcomes—in general (Holly, 2021).

In Figure 1 we propose a hypothetical causal pathway of effects of changes in Hb on various outcomes, and show the individual effects we intend to investigate. Since we did not find comprehensive summaries of existing research on this topic, we aimed to review reviews that examined the effects of Hb modulating interventions on physiology in anaemic and non-anaemic subjects, assess whether sufficient evidence is available for evidence based guidelines, and therewith to identify gaps in existing evidence.

## Methods

### Protocol and registration

An umbrella review was conducted, based on the Joanna Briggs Institute methodology (Ioannidis, 2009). The predefined protocol, published in PROSPERO International prospective register of systematic reviews (CRD42022299392), adhered to the PRISMA-P guidelines (Moher et al., 2009).

### Information sources and search strategy

PubMed, MEDLINE (OVID), Embase, Web of Science, Cochrane Library and Emcare were searched from inception to the 15th of April 2022 for studies that reported on physiological and patient reported outcomes following a Hb change. Additionally, we used hand-searching and screened reference lists and guidelines for finding additional potential inclusions for our umbrella review. There were no language or publication year restrictions. The detailed search strategy is reported in Supplementary Material S6.

### Eligibility criteria and outcomes

Studies were eligible if they were published systematic reviews (SRs) or meta-analyses which included primary studies that evaluated the clinical effect of interventions that change the Hb-level. Non-systematic reviews, reviews in which no Hb levels were reported, or in which Hb change was solely due to blood loss in surgery or other forms of acute bleeding (other than intended phlebotomies) were excluded. We aimed to include systematic reviews on all types of anemias, non-anemias and polycythemia. Eligible interventions that change the Hb level are transfusion, phlebotomy, isovolumic hemodilution, altitude training, EPO, and iron and vitamin supplementation.

Outcomes that were recorded in this umbrella review are: VO_2_max, heart rate, respiratory rate, heart rate variability, exercise capacity, fatigue, patient reported outcome measures, patient reported experience measures, quality of life and hematological outcomes (Hb/Hct/transfusions). Within-subject designs and placebo/standard of care/other comparator-controlled designs are included. Screening, data extraction and quality assessment was performed independently in duplicate by two authors (RT and LZ). Discrepancies were discussed and when necessary, resolved with the help of a third author.

### Data management and selection process

We screened all titles and abstracts of retrieved citations to assess suitability for inclusion. Two authors (RT and LZ) independently screened the full texts of articles potentially meeting eligibility criteria. Before data extraction, articles meeting eligibility criteria were checked whether they were qualitatively sufficient using the JBI Critical Appraisal checklist for systematic reviews and research syntheses. If articles were scored less than 33% on the JBI checklist, they were excluded. In case of discordant reviews, the Jadad decision algorithm was used to ascertain which meta-analysis represented the best evidence (Jadad et al., 1997). CADIMA software (Julius Kühn Institute, Germany) was used to manage records and data. The data extraction was conducted according to a modified JBI extraction tool (Supplementary Material S6). Author, year, type of participants, control groups, number of participants, description of intervention and control, number of studies, types of studies, databases searched, country of origin of the included studies, outcomes and findings, type of effect model used in the meta-analysis (fixed or random), between study heterogeneity estimates (I^2^ values), any presented measure of publication bias, Grading of Recommendations Assessment Development and Evaluation (GRADE) rating, and sources of funding and conflicts of interest were extracted from the studies. We assessed whether the studies evaluated possible sources of heterogeneity across studies and whether the authors conducted a sensitivity analysis.

### Assessment of methodological quality and quality of evidence

Methodological and reporting quality was assessed using the Assessment of Multiple Systematic Reviews (AMSTAR)-2 tool. This tool rates systematic reviews/meta-analysis on literature search, study selection, data extraction and transparency of the studies (Shea et al., 2007). The GRADE score was utilized to score the quality of the evidence found in the included studies (Guyatt et al., 2009). We evaluated the quality on five items: risk of bias, imprecision, inconsistency, indirectness and publication bias using the GRADE Handbook (Schünemann et al., 2013).

### Data synthesis and meta-biases

We performed a narrative umbrella review. Meta-analyses were not feasible with the included heterogenic data. However, forest plots of PROMs and physiological outcomes were conducted without a meta-analysis for overview purposes. In these forest plots, the effect sizes have been transformed to standardized mean differences (SMD) using R’s “MBESS” and “esc” packages, depending on available data. If sample sizes for intervention and control groups were not available separately, we assumed equal groups to estimate the confidence interval for the SMDs. Moreover, with these computed SMDs, we produced funnel plots for PROMs and physiological outcomes separately, to evaluate the possibility of publication bias.

### Sensitivity analysis

Though a formal sensitivity analysis was not possible without a meta-analysis, we assessed whether outcomes were notably different when selecting only high-quality SRs, to reduce confounding and bias, or when leaving out SRs that evaluated ESAs. ESAs, a group of drugs that has been evaluated in larger studies with more pharmaceutical company sponsorship, may give biased results due to conflicts of interests, or contrastingly, less-biased more adequate results due to proper financing for larger, methodological superior trials.

## Results

### Review selection

**FIGURE 2:**
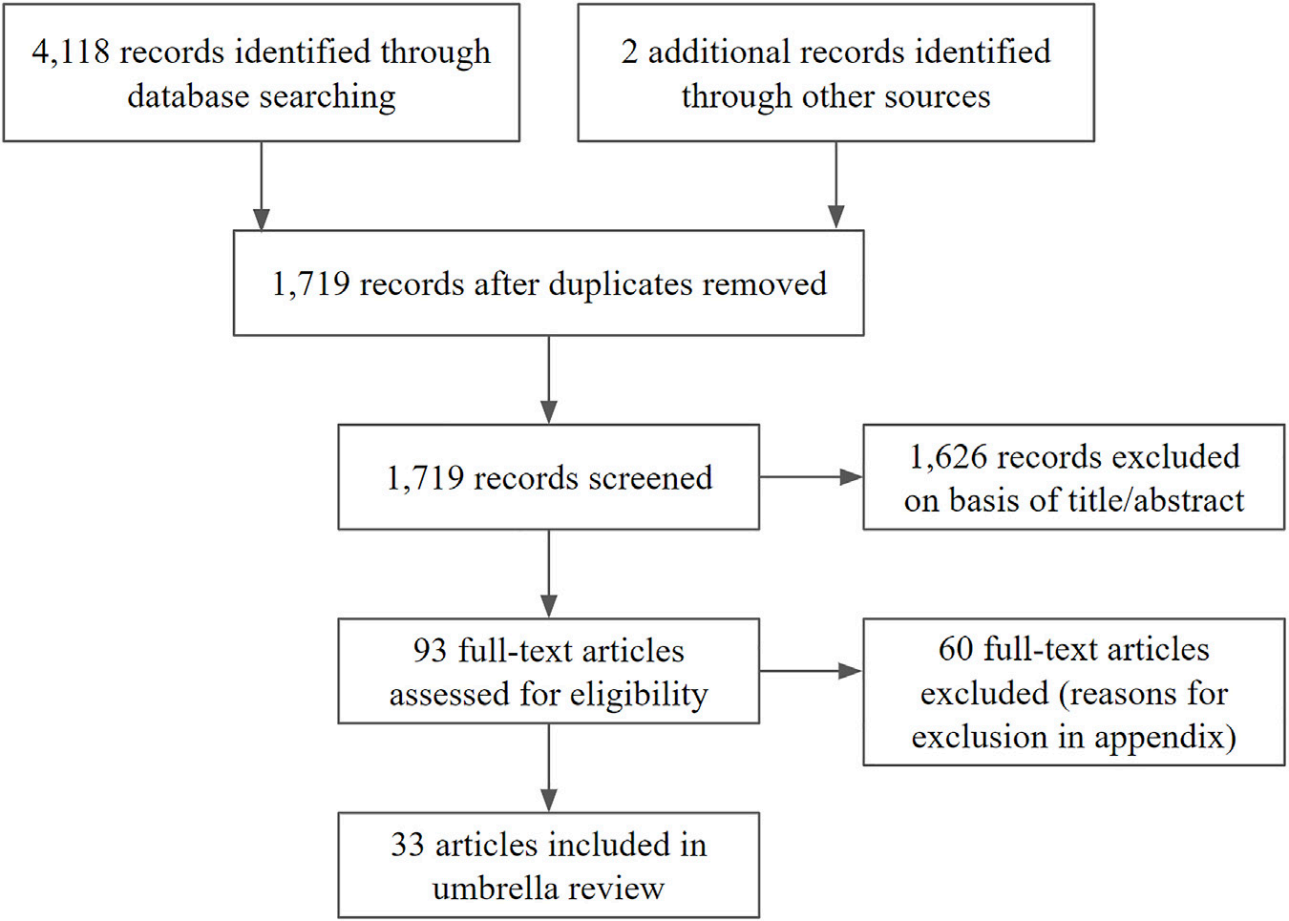
Flow diagram of study selection.

The search strategy yielded 4,120 results. After screening of titles and abstracts, the full texts of 93 articles were further evaluated. Of these 33 studies with 169,878 participants met the inclusion criteria (Figure 2). The included studies were published from 2009 to 2020. The majority of primary studies were conducted in Europe and North-America and 24% (8/33) of the included reviews declared pharmaceutical company sponsorship. We included quantitative reviews with meta-analyses for the interventions ESAs, iron supplementation, altitude training and blood donation. The included patient groups comprising patients with cancer, hematological malignancies, bone marrow failure, chronic kidney disease, chronic heart failure, autoimmune disorders, critically illness in the ICU, iron-deficiency and healthy subjects. We did not encounter suitable SRs that covered hemochromatosis, polycythemia, thalassemia, sickle cell disease and other hemoglobinopathies and subtypes of anemias. For red cell transfusion only narrative reviews were included. No relevant reviews were encountered on the subjects of vitamin supplementation and phlebotomy. Details of studies included for review are shown in Supplementary Material S1. A full reference list of the excluded studies with reasons for exclusion is provided in Supplementary Material S2.

### Critical appraisal and risk of bias

**FIGURE 3:**
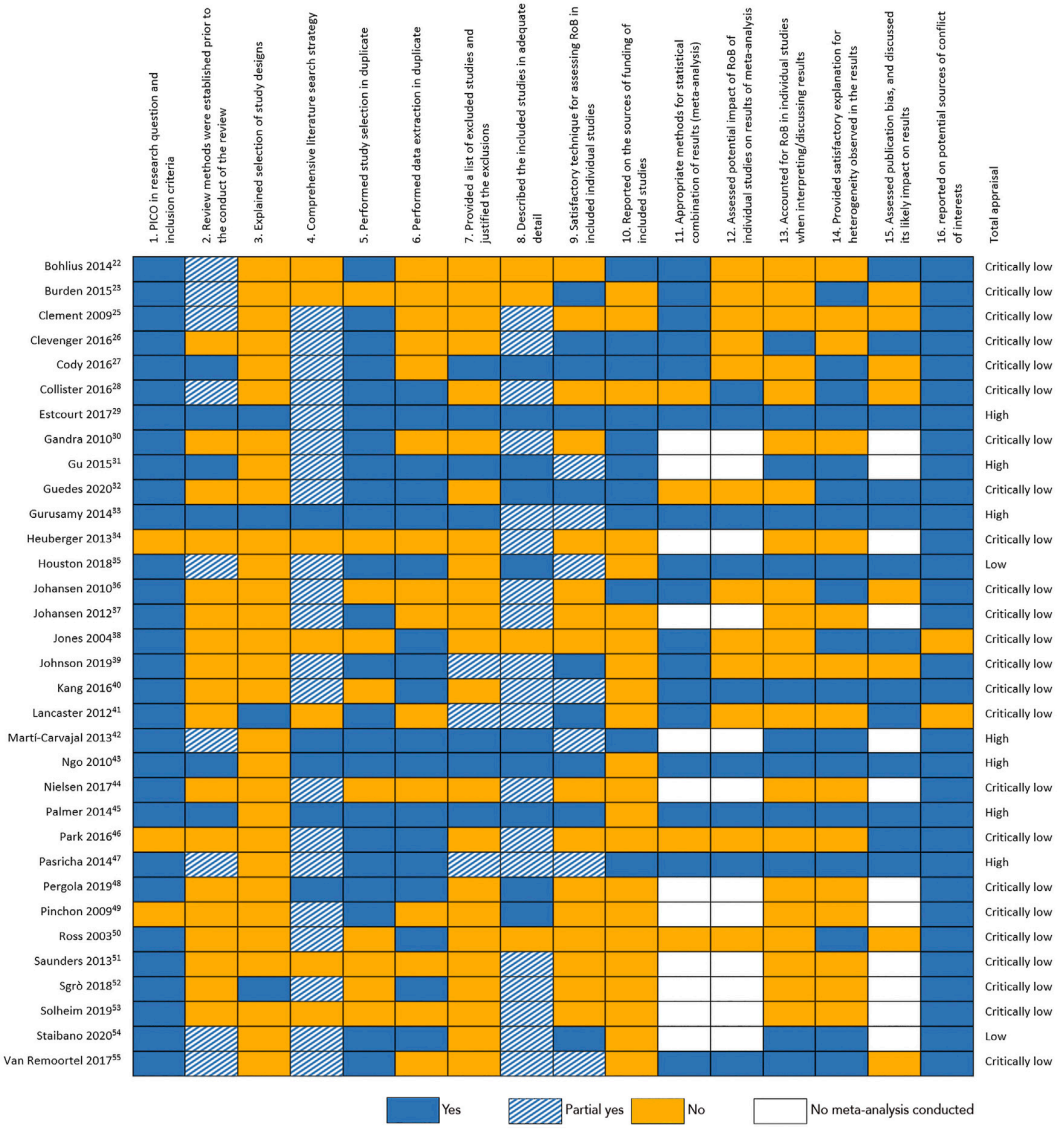
Individual systematic review assessment with the AMSTAR-2 tool.

The individual assessment for each SR with the AMSTAR-2 critical appraisal tool is shown in Figure 3. The quality was found to be *high* in seven SRs, two SRs scored *low* and 24 SRs were appraised as of *critically low* quality. Furthermore, we plotted all quantitative data in funnel plots to assess possible publication bias (Supplementary Figures S1, S2). For physiological outcomes, we found no signs of publication bias (Egger’s test: *p* = 0.277). Patient reported outcome measures, however, showed clear signs of publication bias. The funnel plot in Supplementary Figure S1 has a very pronounced asymmetry, with less powerful studies finding larger effect estimates, suggesting substantial bias. This is supported by the Egger’s test: *p* < 0.001.

**FIGURE 4:**
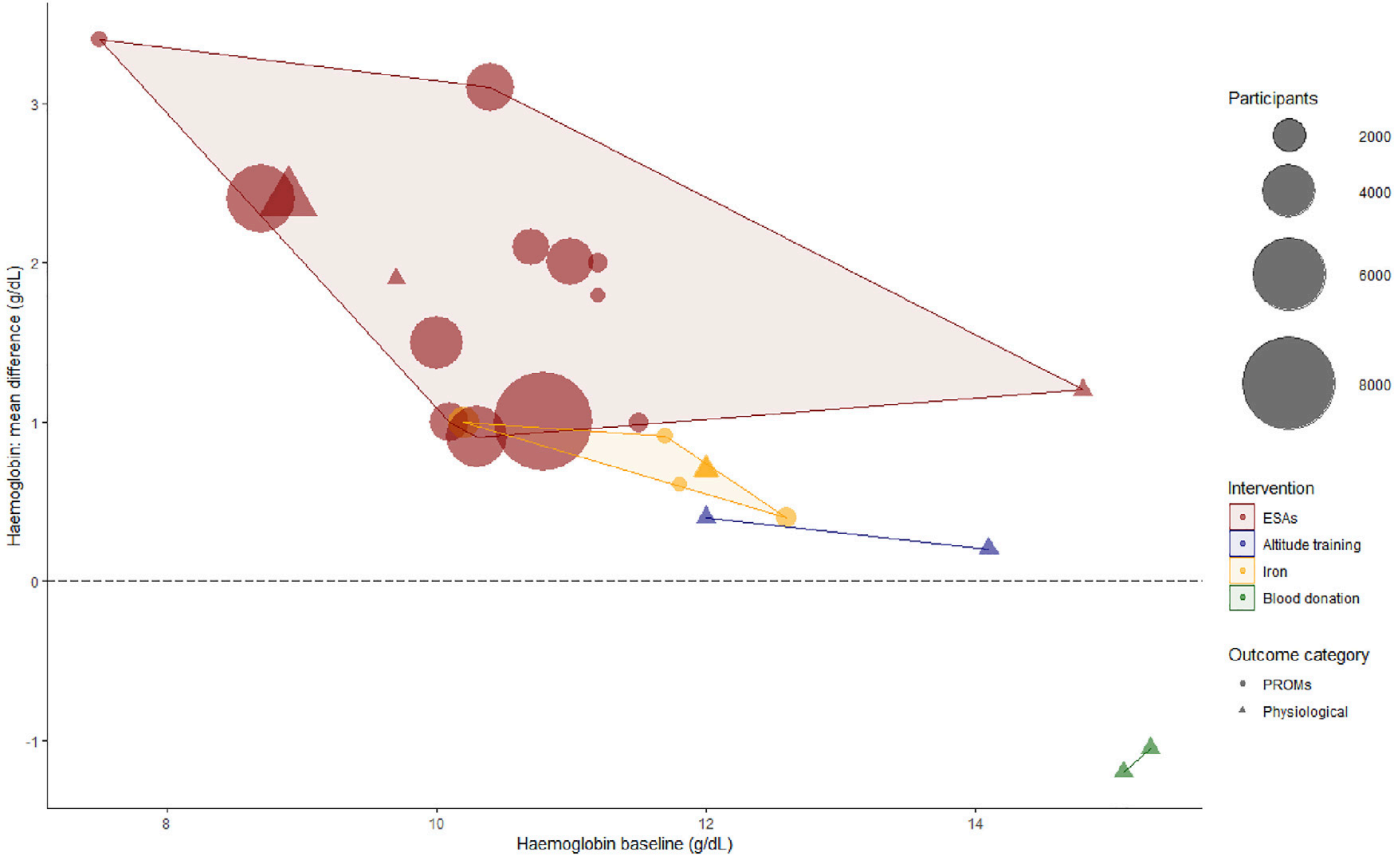
Bubble plot of hemoglobin baseline (pre-intervention) and hemoglobin mean difference (post-intervention—pre-intervention). At lower baseline levels, higher Hb-increments were achieved. Furthermore, systematic reviews evaluating ESAs contributed the majority of included evidence. For the purpose of overview, when a Hb mean difference was not reported, we used the (target—baseline) instead in this figure. Moreover, not all baseline values were reported, in which case we used inclusion criteria cut-offs. As 10/3 three studies only reported heterogenetic data of primary studies or percentages without an absolute Hb-level, we could only include 26/33 reviews in this figure. ESAs, erythropoietin stimulating agents; PROMs, patient reported outcome measures.

An overview of the baseline Hb-levels and the achieved Hb-increment by intervention per study is presented in Figure 4. The mean increase in Hb after ESA-treatment lies between 1 and 3 g/dL. Iron treatment and altitude training show mean increases in Hb of <1 g/dL. The colored areas in this figure indicate per intervention what increment per baseline-Hb has been studied. These areas do not overlap, creating a patchwork-like image. While the lack of overlap shows that we cannot accurately compare the interventions with one another, this supports the combining of these data: to get a more complete picture of the effect of various increments at different baseline-Hb’s.

### ESA treatment

**FIGURE 5:**
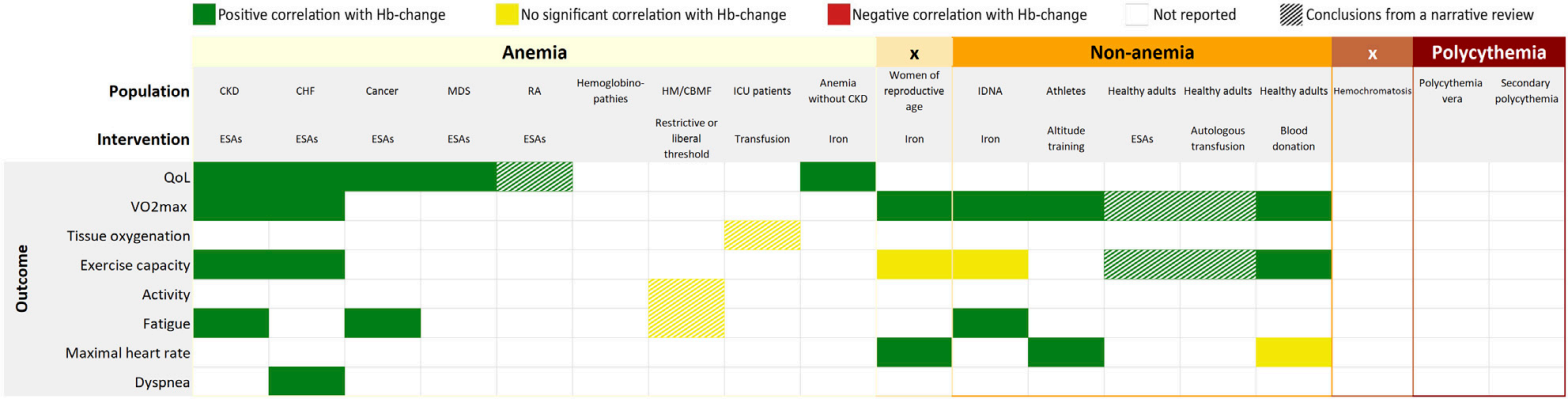
Overall findings of the umbrella review by outcome measures and interventions. CKD, chronic kidney disease; CHF, chronic heart failure; RA, rheumatoid arthritis; HM, hematological malignancies; CBMF, chronic bone marrow failure; ICU, intensive care unit; IDNA, iron deficiency non-anemia; ESAs, erythropoiesis stimulating agents.

**FIGURE 6:**
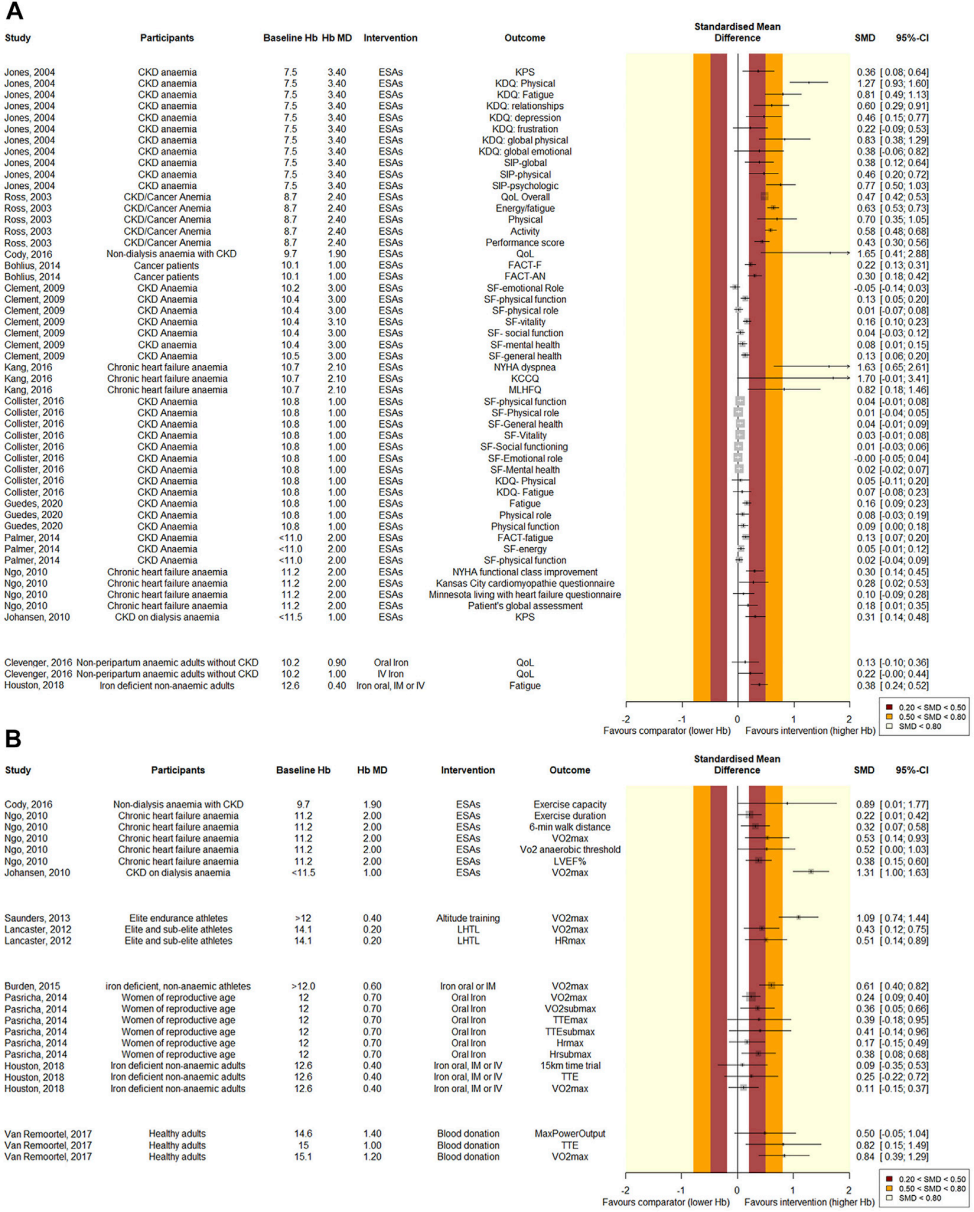
Forest plot of quantitative **(A)** PROM and **(B)** physiological outcomes. Results are grouped per intervention, listed from lowest to highest baseline hemoglobin level. The effect of Hb-changes appears more pronounced at lower baseline Hb-levels. Standardized mean differences were computed by transforming the reported effect sizes using R “MBESS” or “esc” packages, depending on the available data. SMD values of 0.2–0.5 are considered small, 0.5–0.8 medium, and values >0.8 are considered large. For overview purposes, if a Hb mean difference was not reported, we used the target instead in this figure. Also, not all baseline values were reported, in which case we used inclusion criteria cut-offs.

Twenty citations that investigated the effect of ESA treatment were eligible for evaluation (Supplementary Materials S1, S3). The SRs were published between 2003 and 2020, and AMSTAR-2 quality of reporting ranged from critically low to high, the GRADE quality of evidence was generally low, ranging from very low to high. ESAs were compared with placebo, lower targets or no ESAs. Results are depicted in Figures 5, 6. Due to heterogeneity of the investigated subjects, we summarized the outcomes per patient group (Supplementary Figures S3, S4).

#### Chronic kidney disease

Eight quantitative (Jones et al., 2004; Ross et al., 2007; Clement et al., 2009; Johansen et al., 2010; Palmer et al., 2014; Cody and Hodson, 2016; Collister et al., 2016; Guedes et al., 2020) and four qualitative (Gandra et al., 2010; Johansen et al., 2012; Pergola et al., 2019; Staibano et al., 2020) SRs that investigated chronic kidney disease patients were included. QoL, VO_2_max, exercise capacity and fatigue were reported to improve with an increase in Hb in quantitative analyses. Intriguingly however, the review with the most participants found no significant correlation between the Hb-change and PROMs, although the most important items did approach significance (SF-General health: MD = −1.9, CI: −4.1, 0.4, SF-physical function: MD = −2.6, CI: −5.6, 0.4, SF-Vitality: MD = −2.0, CI: −4.4, 0.4). (Collister et al., 2016). Effects of Hb-modulation on PROMs at baseline levels >10 g/dL appear less pronounced than at Hb-levels <10 g/dL. However, the increase in Hb-level is greater at lower levels than at higher baseline levels. Furthermore, two SRs reported that HRQoL benefits associated with hemoglobin targets of >12 g/dL appeared modest and of uncertain clinical significance (Clement et al., 2009; Pergola et al., 2019).

#### Anemia with underlying malignancy

One quantitative SR of *critically low* quality investigated ESA-induced Hb-modulation in patients with underlying malignancies. Fatigue and QoL improved after an increase in Hb from a mean baseline of 10.1 g/dL (Bohlius et al., 2014). Two qualitative SRs reported on Hb-modulation in patients with myelodysplasia, reporting that patients that had a hematological response to ESAs improved on QoL-scores.

#### Other: Chronic heart failure, healthy subjects, rheumatoid arthritis

Two quantitative SRs investigating chronic heart failure patients reported that QoL, VO_2_max, exercise capacity and dyspnea improved significantly after a mean Hb increase of 2.0–2.1 g/dL at a mean baseline Hb of 10.7–11.2 g/dL (Ngo et al., 2009; Kang et al., 2016). In healthy subjects, two qualitative SRs evaluated the effect of ESAs on VO_2_max, exercise capacity and the maximal heart rate. All these parameters were suggested to be positively affected by an increase in Hb, although no meta-analyses were performed to support this (Heuberger et al., 2013; Sgrò et al., 2018). Finally, in rheumatoid arthritis, QoL was suggested to be positively corelated to Hb in a qualitative SR (Martí-Carvajal et al., 2013).

### Iron treatment

Five SRs evaluating iron treatment were eligible (Supplementary Materials S1, S3) (Gurusamy and Richards, 2013; Pasricha et al., 2014; Burden et al., 2015; Clevenger et al., 2016; Houston et al., 2018). Treatment with intravenous, oral and intramuscular iron was compared with placebo, iron oral, or no iron in anaemic and non-anaemic patient groups. The articles were published between 2013 and 2018. AMSTAR-2 scores ranged from *critically low* to *high* and the evidence was graded *very low* to *moderate*. In Figures 5, 6 the effects of iron on various outcomes are depicted. Clevenger et al. investigated the effect of iron on QoL in anaemic patients without chronic kidney disease and found that these patients benefit significantly from parental iron (Hb MD = 1.04 g/dL, CI: 0.52, 1.57, *n* = 1744), but oral application (Hb MD: 0.91 g/dL, CI: 0.48, 1.35, *n* = 851) did not lead to a significant difference in QoL. Pasricha et al. (2014) evaluated studies with women of reproductive age, anaemic and non-anaemic together, and concluded that oral iron supplementation increased the Hb-level and had a significantly positive effect on VO_2_max (MD = 2.35, CI: 0.82, 3.88) and the heartrate (MD −4.05 CI:−7.25, −0.85). Pasricha et al. (2014) also mentioned the role of iron in the mitochondrial respiratory chain, suggesting that improved tissue iron might improve exercise capacity, independent of a Hb-modulation. The exercise capacity showed a positive trend, although this was not significant. Two SRs evaluated iron treatment in iron deficient non-anaemic groups. These groups significantly benefited from the iron-induced Hb-change for the outcomes VO_2_max and fatigue. The exercise capacity however, again did not change significantly.

### Transfusion

Two narrative SRs were eligible for evaluation with regard to transfusion (Supplementary Materials S1, S3). Nielsen et al. (2017) evaluated the effect of transfusion on tissue oxygenation in critically ill ICU patients with anemia compared to similar controls. They found no significant effect on tissue oxygenation (Figure 5). However, nine of the primary studies revealed a consistent pattern—patients with abnormal tissue oxygenation prior to transfusion improved with transfusion, whereas those with normal pre-transfusion indices did not. The authors conclude that this supports a strategy for future clinical trials which focuses on abnormalities of tissue oxygenation or microcirculatory parameters rather than hemoglobin levels as the trigger for transfusion. Solheim et al. (2019) evaluated the effect of autologous transfusion on the VO_2_max and exercise capacity in trained and untrained adults, compared to controls that did not receive an autologous blood transfusion. They reached the conclusions that the magnitude of change in Hb explains the increase in VO_2_max, and reinfusion with as little as 135 mL packed red blood cells increases time-trial performance by elevating CaO_2_. The AMSTAR-2 scores were *critically low* for both SRs, the GRADE quality of evidence was *very low*.

### Restrictive *versus* liberal threshold

Of the retrieved citations evaluating restrictive (7.0–9.0 g/dL) and liberal thresholds (9.6–12.0 g/dL), two SRs were eligible for review (Supplementary Materials S1, S3). The SRs were published in 2015 and 2017, and both evaluated the effect of transfusion thresholds on quality of life. Estcourt et al. (2017) concluded to be very uncertain whether a restrictive threshold reduces quality of life in a population with hematological malignancies: one trial, 89 participants, fatigue score: restrictive median 4.8 (IQR 4–5.2); liberal median 4.5 (IQR 3.6–5) (*very low*-quality evidence). Gu et al. (2015) aimed to evaluate fatigue in a myelodysplastic syndrome population, but reported only one study which was terminated early due to poor recruitment and reported no results for their primary outcome measure. Both SRs are of *high* quality as scored with the AMSTAR-2 tool, but produced *very low* GRADE quality evidence due to small sample sizes and low quality primary studies.

### Altitude training

Two SRs, published in 2012 and 2013, evaluated altitude training in athlete populations (Supplementary Materials S1, S3). Whereas Saunders et al. (2013) investigated several types of altitude training together, Lancaster and Smart (2012) focused only on live-high train-low altitude training. The AMSTAR-2 scores for both SRs were *critically low*, and GRADE quality of the evidence was *low*. Both SRs reported a small increase in Hb (0.57–0.69 g/dL and ∼3%). Lancaster reported an improvement in maximal heart rate (MD = 1.77, CI:0.50, 3.03) and both SRs concluded that altitude training improves VO_2_max significantly (MD = 1.51, CI: 0.44, 2.58 and slope = 0.48, CI: 0.30, 0.67) in athletes, compared to normoxic training (Figures 5, 6).

### Blood donation

Two SRs evaluating blood donation were eligible for review (Supplementary Materials S1, S3). The AMSTAR-2 scores of both articles were *critically low*. The GRADE quality of the evidence ranged from *very low* to *low*. (Van Remoortel et al. (2017) evaluated several studies that compared healthy adults that underwent blood donation before and after donation, and one study that used sham bleeding as comparator. Johnson et al. (2019) performed a similar SR with comparable eligibility criteria. The results and conclusions presented by Johnson et al. (2019) were not completely understood, and Johnson et al. (2019) could unfortunately not be reached for comments. Intriguingly, Johnson and Van Remoortel reached contrasting conclusions. With much overlap in primary studies, we selected the review with the best data synthesis on the basis of the JADAD decision algorithm. Hence, we selected Van Remoortel’s SR. The latter concluded that the VO_2_max and time to exhaustion decreased significantly after blood donation. Maximum power showed no significant difference pre- and post-donation (Figures 5, 6).

### Sensitivity analysis

Systematic reviews evaluating ESAs make up for a large part of the included evidence. Therefore, we decided to additionally evaluate what the outcome of this umbrella review would have been without these SRs evaluating ESAs. A forest plot, funnel plot and an overview table of results, all without ESA-related trials, are shown in Supplementary Material S5a (Supplementary Figures S5–S8). Remarkably, very little evidence remains regarding PROMs and regarding anaemic patients in general. Too few reviews could, however, be included to create a funnel plot for PROMs in this sensitivity analysis. Non-etheless, there is no evident change of significance direction notable.

Furthermore, for a second sensitivity analysis, we selected only *high*-quality SRs. In doing so, no data for non-anemia were left (Supplementary Figures S9–S12). For anaemic patients, however, the outcomes again remained similar in this analysis.

## Discussion

The present umbrella review examined the body of evidence on the effect of changes in Hb levels on physiological and patient reported outcomes. The gathered evidence suggests that an increase in Hb is generally followed by better physiological and patient reported outcomes (Figures 5, 6). Likewise, a decrease in Hb would lead to inferior outcomes. The magnitude of the effect appears more pronounced at lower baseline Hb-levels. Although, the increment was also higher at lower baseline Hb-levels, which might be an important confounder. Despite rigorous and systematic methodology, we were only able to identify 33 eligible systematic reviews, mainly reporting on the effect of ESAs. Therefore, as illustrated by Figure 5, our umbrella review reveals many gaps in evidence. Of many interventions, the effects are not yet well evaluated. The added value of this umbrella review, illustrated by Figure 4, is that through a patchwork of data from studies that evaluate Hb-changes with different interventions, we can imagine an extrapolated effect in our knowledge gaps. The present overview thus provides us with what might be expected of the general effect of a Hb-shift, regardless of the cause/intervention, which should be considered in the light of numerous patient-specific factors that affect the outcome for the individual. More importantly, this overview demonstrates that currently there is very little high quality evidence on this subject and identifies the knowledge gaps that require further research.

The domains of fatigue and physical functioning are reported to be affected most in PROMs. When focusing on physiological outcomes, VO_2_max and exercise capacity have been studied most frequently, but hardly in patient groups with Hb-levels <10 g/dL. While a trend towards a positive correlation between Hb-increment and physiological outcomes is apparent, this is not always significant, possibly due to underpowering. VO_2_max improves significantly with an increase in Hb in both anaemic and non-anaemic patients. Exercise capacity is not always improved after an increase in Hb. Perhaps, longer standing Hb-improvements are needed to have a noticeable effect on the exercise capacity. While a reduction in fatigue and increase in VO_2_max will lead to increased activity, an increased muscle mass will only ensue later.

We found no eligible SRs evaluating the effect of a Hb-change on physiological outcomes in polycythemia patients. Intriguingly, no SRs evaluated the effect of Hb-changes on respiratory rate, resting heart rate, heart rate variability, cardiac output or other basic physiological outcomes in any of the patient groups.

### Limitations

The reporting of evidence is generally scored by the AMSTAR-2 checklist to be of *critically low* quality, and meta-analyses with substantial sample sizes are mostly restricted to one intervention: ESAs. We did not impose any restrictions by language or publication year and used very wide eligibility criteria to decrease the chance of selection bias. However, because of the wide eligibility criteria for this overview, the included populations are very heterogeneous. It is therefore emphatically not our intention to compare the included reviews with each other. Likewise, all quantitative outcomes—from VO_2_max to PROMs—have been presented together in the forest plots in Figure 6. The intention here is to give a visual overview of the standardized effect sizes of all results, not for meta-analytic purposes. Moreover, due to incomplete reporting in several reviews, multiple assumptions had to be made to compute SMDs from the reported effect sizes, limiting possibilities and accuracy of deeper analyses. Non-etheless, we believe this gives a good overview of the available evidence. Furthermore, it was commonly noted by the included narrative SRs that studies and outcomes were often too heterogenous to be compared in meta-analyses. International cooperation and standardization of methods for producing and reporting outcomes would thus be helpful to reach more meaningful conclusions and to compare outcomes with other trials. As per custom for umbrella reviews, we chose to only include systematic reviews and meta-analyses to compare and contrast published reviews and to provide an overall examination of a body of information that is available for this topic (Hartling et al., 2012). Therefore, primary studies may exist that have assessed areas that we identified as gaps in evidence. Reviewing primary studies in these identified gaps, preferably in a meta-analysis, may be a next step for future research. Furthermore, the Hb-increment was higher in groups with a lower baseline Hb than in groups with a higher baseline Hb (Figure 4). This is important to realize when interpreting the forest plots in Figure 6: the effect of a Hb-change seems more pronounced at lower baseline-levels. However, as the increment is higher at lower baseline Hbs, a dose-dependency effect might have confounded this result. Other bias may have been introduced by side-effects of the investigated therapies. With current techniques it is impossible to determine whether an improvement in QoL is caused solely by a Hb modulation. It is conceivable that other metabolic effects of iron supplementation or treatments like ESAs may cause this change in QoL.

Our meta-bias analysis suggests that reviews analyzing PROMs are prone to publication bias, whereas physiological outcomes do not show meta-biases in the funnel plot or sensitivity analysis. Indeed, PROMs are a subjective tool, and therefore easily biased if the methodology of the primary study lacks blinding and such. Possibly, larger primary studies were methodologically sounder than those with small sample sizes, in which case, the funnel plot is a result of information bias. The other option is that non-significant meta-analyses were not published. Either way, as this issue did not occur with the physiological outcomes, we recommend for future primary studies to at least incorporate physiological outcomes, preferably in combination with PROMs. With fast emerging wearables, eHealth and remote monitoring options (Tonino et al., 2019), incorporating physiological outcomes has rapidly become more feasible and may provide intriguing new insights.

Our sensitivity analyses underline the fact that there is a lack of high-quality evidence to support guidelines. Selecting high quality SRs only, the results for anaemic patients were similar. However, there were no high quality SRs that assessed Hb-changes in non-anaemic patients. After removing ESA-related reviews there were hardly any reviews left that assessed PROMs and very little evidence for anaemic patients in general. Since ESA-studies are predominantly funded by pharmaceutical companies, this demonstrates the importance of funding by pharma for research. Even though pharma-funded studies may be prone to conflicts of interest, they do contribute significantly to current knowledge. Non-etheless, independent high-quality evidence is very scarce.

In search of evidence based guidance for hemoglobin optimalization, whether for transfusion thresholds, ESA-targets, hyperviscosity management or optimal athletic performance, we found that for a quantitative benefit-harm assessment, we lack the data to draw meaningful conclusions. In some cases, as in ESA treatment in chronic kidney disease, there are sufficient data for a moderately certain conclusion that there is benefit in increasing the Hb-level up until about 12 g/dL for both patient reported and physiological outcomes. With the heterogeneity, confounding and scarce high quality evidence, it is difficult to compare and/or extrapolate these findings to Hb-levels >12 g/dL. Additionally, due to data suggesting an increased thrombotic risk above 12.0 g/dL in ESA-treated chronic kidney disease-patients, we would advise against Hb-targets >12.0 g/dL in this patient group (Palmer, 2010). For other treatments and patient groups, and the subjects of donation and phlebotomies, the evidence is thin or non-existent, and no conclusions about optimal Hb-levels can be drawn.

Critics may state that the Hb level is an inadequate measure to begin with. Indeed, the total Hb-mass is not affected by changes in plasma volume and would be more stable and render more accurate insights (Prommer et al., 2008). However, most studies use the Hb level as a proxy for total Hb-mass due to its ready availability. And while less accurate, it can be insightful in larger samples. In the future, however, we need to look at alternative objective parameters for oxygen transport optimization. With current technology, wearable devices are able to remotely measure basic physiology parameters like heart rate, heart rate variability, respiration rate, tissue oxygenation, capillary oxygenation and many more. Collecting these parameters and using big data algorithms may be a step towards a more accurate assessment of the patients’ treatment requirements when it comes to oxygen transport optimization. As this umbrella review shows, we still have many data gaps to fill.

In conclusion, this overview has revealed many knowledge gaps due to a lack of high-quality evidence. For chronic kidney disease patients, a clinically relevant benefit of increasing the Hb levels up until at least 12 g/L on PROMs and physiological outcomes was found. However, a personalized approach of patient care remains a necessity due to the many patient-specific factors that can affect the tolerance to deviating Hb-levels. In non-anaemic patients, the evidence suggests that increasing the Hb-level improves physiological and patient reported outcomes, but the effect is less pronounced. Decreasing the Hb-level has a clear inverse effect on physiological outcomes in non-anemic subjects. For supraphysiological Hb-levels, no eligible reviews were found. Because of the many knowledge gaps in general in the reported data sets, we strongly encourage future trials to incorporate physiological outcomes as objective parameters together with subjective, but still very important, patient reported outcome measures.
