# Supplementary material for: Hemoglobin modulation affects physiology and patient reported outcomes in anemic and non-anemic subjects: An umbrella review
Source: Front Physiol. 2023 Feb 15;14:1086839. doi: 10.3389/fphys.2023.1086839 (PMC9975154; doi:10.3389/fphys.2023.1086839)
Supplement: Supplementary file 1 [file DataSheet1.docx]

**Supplementary material**

[Supplementary material 1: Included studies 2](#_Toc125967690)

[Supplementary material 2: Excluded studies with reason for exclusion 15](#_Toc125967691)

[Supplementary material 3: Outcomes per comparison 1](#_Toc125967692)7

[**Comparison 1.** ESAs vs placebo, lower target, or no ESAs 17](#_Toc125967693)

[**Comparison 2.** Iron vs placebo, Iron IV vs oral, or no iron 21](#_Toc125967694)

[**Comparison 3.** Pre-transfusion vs. post-transfusion 22](#_Toc125967695)

[**Comparison 4.** Restrictive transfusion threshold vs. liberal threshold 22](#_Toc125967696)

[**Comparison 5.** Altitude training vs. no altitude training 22](#_Toc125967697)

[**Comparison 6.** Before blood donation vs. post-donation 23](#_Toc125967698)

[Supplementary material 4. Funnel plots 24](#_Toc125967699)

[Supplementary material 5. ESA outcomes per patient group 25](#_Toc125967700)

[Supplementary material 6. Sensitivity analysis figures 26](#_Toc125967701)

[**Sensitivity analysis 1:** Outcomes without reviews assessing ESAs 26](#_Toc125967702)

[**Sensitivity analysis 2**: Selection of high quality systematic reviews, as scored by the AMSTAR-2 tool. 28](#_Toc125967703)

[Supplementary material 7. Extraction form 30](#_Toc125967704)

[Supplementary material 8. Search strategy 31](#_Toc125967705)

[Supplementary material 9: PRIO tool 5](#_Toc125967706)6

[Supplementary references 60](#_Toc125967707)

# **Supplementary material 1:** Included studies

**Bohlius, J. 2014**

| Type of participants | Cancer patients receiving or not receiving anticancer treatment. |
| --- | --- |
| Control group | Placebo or best standard of care. |
| Number of participants | 10,581 |
| Description of interventions | Epoetin or darbepoetin, various doses. |
| Number of studies | 37 |
| Types of studies | Randomized controlled trials. |
| Country of origin of studies | Not reported. |
| Outcome assessed | FACT-F, FACT-AN and FACT-G. |
| Funding | Funded by OncoSuisse, grant number OCS-02232-04-2008. |
| Last search date | January 2011 |
| Language and publication restriction | Published and unpublished data was included. Language restriction not reported. |
| Appraisal instruments | Not reported |

**Burden, R. 2015**

| Type of participants | Athletes who were classified as being iron deficient but non-anaemic. |
| --- | --- |
| Control group | No iron treatment or receiving placebo treatment. |
| Number of participants | 443 |
| Description of interventions | Oral or intramuscular iron 20-418 mg/day for 8-168 days. |
| Number of studies | 17 |
| Types of studies | Randomized controlled trials and non-randomized controlled trials. |
| Country of origin of studies | Not reported |
| Outcome assessed | Hb, sFer, Tsat, sFe and VO_2_max. |
| Funding | None |
| Last search date | November 2013 |
| Language and publication restriction | Not reported |
| Appraisal instruments | Cochrane Risk of Bias tool |

**Clement, F.M. 2009**

| Type of participants | Patients with chronic kidney disease |
| --- | --- |
| Control group | Lower Hb-target or placebo. |
| Number of participants | 3784 |
| Description of interventions | Epoetin or darbepoetin |
| Number of studies | 11 |
| Types of studies | Randomized controlled studies |
| Country of origin of studies | Not reported |
| Outcome assessed | HQOL |
| Funding | AHFMR & CIHR |
| Last search date | 12^th^ December 2006 |
| Language and publication restriction | No language restriction. Publication restriction is not reported. |
| Appraisal instruments | JADAD-5 points |

**Clevenger, B. 2016**

| Type of participants | Non-pregnant and non-lactating anaemic adults without chronic kidney disease, irrespective of setting and degree of anaemia. |
| --- | --- |
| Control group | Control treatment or placebo treatment. |
| Number of participants | 9004 |
| Description of interventions | Intravenous or oral iron. |
| Number of studies | 65 |
| Types of studies | Randomized controlled trials |
| Country of origin of studies | Not reported |
| Outcome assessed | Mortality primarily, Hb and QoL. |
| Funding | Funded by a National Institute for Health Research Health Technology Assessment (NIHR HTA) grant, Pharmacosmos and Vifor. |
| Last search date | November 2014 |
| Language and publication restriction | No language and publication status restrictions |
| Appraisal instruments | Cochrane risk of bias tool. |

**Cody, J. 2016**

| Type of participants | Patients with anaemia of chronic kidney disease who have not yet commenced dialysis. |
| --- | --- |
| Control group | Placebo treatment or not receiving rHuEPO. |
| Number of participants | 993 |
| Description of interventions | Treatment with rHuEPO irrespective of dose or mode of delivery. |
| Number of studies | 19 |
| Types of studies | Randomized controlled trials or quasi-randomized controlled trials. |
| Country of origin of studies | USA, Japan, Sweden, India, Korea, Israel, Czech Republic and Hong Kong. |
| Outcome assessed | Hb, Hct, QoL. |
| Funding | None |
| Last search date | 29^th^ June 2015 |
| Language and publication restriction | Only published data was used. Language restriction was not reported. |
| Appraisal instruments | Cochrane risk of bias tool |

**Collister, D. 2016**

| Type of participants | Patients with Chronic kidney disease who were receiving or not receiving dialysis. |
| --- | --- |
| Control group | Placebo or a low-hemoglobin target. |
| Number of participants | 10,049 |
| Description of interventions | Patients receiving erythropoietin alfa/darbepoetin. |
| Number of studies | 17 |
| Types of studies | Randomized controlled trials. |
| Country of origin of studies | Not reported |
| Outcome assessed | QoL, SF-36, KDQ and Hb-levels. |
| Funding | Funded by KRESCENT and the Manitoba Health Research Council Establishment award. |
| Last search date | 1^st^ November 2015 |
| Language and publication restriction | Not reported |
| Appraisal instruments | Cochrane Collaboration’s tool for assessing risk of bias. |

**Estcourt, L. 2017**

| Type of participants | People diagnosed with hematological malignancies treated with intensive chemotherapy or radiotherapy, or both, with or without hematopoietic stem cell transplantation (HSCT). |
| --- | --- |
| Control group | Liberal/traditional threshold (80-120 g/L) |
| Number of participants | 155 |
| Description of interventions | Restrictive or liberal threshold (70/80 g/L vs. 80-120 g/L) |
| Number of studies | 7 |
| Types of studies | Randomized controlled trials and one NRT. |
| Country of origin of studies | Canada, Holland, USA |
| Outcome assessed | QoL |
| Funding | NHS Blood and Transplant, Research and Development, UK. |
| Last search date | 15^th^ June 2016 |
| Language and publication restriction | No restrictions on language and publication status. |
| Appraisal instruments | Cochrane risk of bias tool. |

**Gandra, S.R. 2010**

| Type of participants | Non-dialysis chronic kidney disease patients |
| --- | --- |
| Control group | Treated vs untreated, placebo, low target or single arm |
| Number of participants | 5010 |
| Description of interventions | ESA treatment |
| Number of studies | 14 |
| Types of studies | Randomized controlled trials and single-arm trials |
| Country of origin of studies | Not reported |
| Outcome assessed | QOL: SF36 questionnaire |
| Funding | AMGEN |
| Last search date | 2008 |
| Language and publication restriction | Only published studies. Language restriction was not reported. |
| Appraisal instruments | None |

**Gu, Y. 2015**

| Type of participants | Patients, irrespective of age, with long-term bone marrow failure disorders that require allogeneic blood transfusion, who are not being actively treated with a hematopoietic stem cell transplant, or intensive chemotherapy. |
| --- | --- |
| Control group | Liberal versus restrictive treatment. |
| Number of participants | 13 |
| Description of interventions | Red blood cell transfusion strategies defined as ’restrictive’ and ’liberal’. |
| Number of studies | 1 |
| Types of studies | Randomized controlled trials. |
| Country of origin of studies | Netherlands |
| Outcome assessed | QoL and effect on activity. |
| Funding | Funded by NIHR Cochrane Program Grant - Safe and Appropriate Use of Blood Components. |
| Last search date | 26^th^ May 2015 |
| Language and publication restriction | No restrictions. |
| Appraisal instruments | The Cochrane Collaboration’s “Risk of bias” tool. |

**Guedes, M. 2020**

| Type of participants | Patients with chronic kidney disease with anaemia and adult patients treated for anaemia with ESA exposed to different Hb targets. |
| --- | --- |
| Control group | Maintaining Hb-levels between 10-11.5. |
| Number of participants | 4,157 |
| Description of interventions | Treatment with ESA with different achieved Hb levels among groups. |
| Number of studies | 20 |
| Types of studies | Randomized controlled trials and observational studies. |
| Country of origin of studies | Not reported |
| Outcome assessed | Fatigue, physical role and physical function. |
| Funding | None |
| Last search date | May 2020 |
| Language and publication restriction | Only published studies in English, Spanish or Portuguese. |
| Appraisal instruments | Cochrane Tool for Bias Assessment. |

**Gurusamy, K. 2015**

| Type of participants | Patients who are non-pregnant and non-lactating, without chronic kidney disease, irrespective of the setting and the degree of anaemia. The following broad categories of participants were included: blood loss, cancer, preoperative anaemia, chronic heart failure, autoimmune disorders |
| --- | --- |
| Control group | Placebo, no treatment, different iron dose or intravenous versus oral iron. |
| Number of participants | 4745 |
| Description of interventions | Intravenous iron, oral iron or various doses |
| Number of studies | 21 |
| Types of studies | Randomized controlled trials |
| Country of origin of studies | Norway, Brazil, UK, Sweden, Australia, Canada, Italy, Lebanon, USA and Mexico. |
| Outcome assessed | Hb and QoL. |
| Funding | Funded by internal sources, UCL Department of Surgery and other. |
| Last search date | July 2013 |
| Language and publication restriction | No language or publication status restrictions. |
| Appraisal instruments | Cochrane Tool for Bias Assessment |

**Heuberger, J.A. 2013**

| Type of participants | Untrained and trained cyclists. |
| --- | --- |
| Control group | Cyclists receiving placebo or no treatment. |
| Number of participants | 156 |
| Description of interventions | RHuEPO subcutaneous injections, most in a similar range of 150 IU kg-1. |
| Number of studies | 18 |
| Types of studies | Not reported |
| Country of origin of studies | Not reported |
| Outcome assessed | Hb, Hct, VO_2_max |
| Funding | None |
| Last search date | Not reported |
| Language and publication restriction | Not reported |
| Appraisal instruments | None reported |

**Houston, B.L. 2018**

| Type of participants | Iron deficient non anaemic adults. |
| --- | --- |
| Control group | Placebo, saline or zinc gluconate |
| Number of participants | 1170 |
| Description of interventions | Oral, intramuscular or intravenous iron supplementation, all therapy doses, frequencies and durations were included. |
| Number of studies | 20 |
| Types of studies | Randomized controlled trials |
| Country of origin of studies | North-America, Europe, Australia & Asia. |
| Outcome assessed | Patient reported fatigue, 15km time trial, time to exhaustion & VO_2_max. |
| Funding | None |
| Last search date | 31 October 2015 |
| Language and publication restriction | Only published trials were included. Language restriction was not reported |
| Appraisal instruments | Cochrane Tool for Bias Assessment |

**Johansen, K.L. 2010**

| Type of participants | Dialysis adult patients with chronic kidney disease |
| --- | --- |
| Control group | Placebo treatment |
| Number of participants | 762 |
| Description of interventions | Treatment with ESA’s |
| Number of studies | 28 |
| Types of studies | Randomized controlled trials and observational studies |
| Country of origin of studies | Not reported |
| Outcome assessed | VO_2_ and physical function |
| Funding | AMGEN |
| Last search date | 2008 |
| Language and publication restriction | Only English published studies. |
| Appraisal instruments | None reported |

**Johansen, K.L. 2012**

| Type of participants | Patients with end stage renal disease anaemia receiving dialysis. |
| --- | --- |
| Control group | Patients receiving placebo. |
| Number of participants | 3082 |
| Description of interventions | ESA’s high target and low target, there was a large variation. |
| Number of studies | 15 |
| Types of studies | Randomized controlled trials and Cohort studies. |
| Country of origin of studies | Not reported |
| Outcome assessed | Hb baseline >10g/dl with 1g/dL increase in HB, <10g/dl with >1g/dl increase & <1g/dl increase. |
| Funding | Funded by Amgen |
| Last search date | 30^th^ June 2010 |
| Language and publication restriction | Only published studies in English language. |
| Appraisal instruments | None reported |

**Jones, M. 2004**

| Type of participants | Patients with chronic renal failure |
| --- | --- |
| Control group | No control |
| Number of participants | 25080 |
| Description of interventions | ESA treatment |
| Number of studies | 16 |
| Types of studies | Randomized controlled trials and observational studies |
| Country of origin of studies | Not reported |
| Outcome assessed | SIP and KDQ |
| Funding | Unknown |
| Last search date | 2000 |
| Language and publication restriction | No language restriction; only published articles. |
| Appraisal instruments | None reported |

**Johnson, D.M. 2019**

| Type of participants | Blood donors > 66 years and < 17 years in age. |
| --- | --- |
| Control group | Sham bleed |
| Number of participants | 101 |
| Description of interventions | 450-500ml blood donation. |
| Number of studies | 8 |
| Types of studies | Case-crossover studies |
| Country of origin of studies | Not reported |
| Outcome assessed | Max Hr, Time till exhaustion, VO_2_max, Hb, HCT, RBCs. |
| Funding | None |
| Last search date | January 2018 |
| Language and publication restriction | Only English publications were used. |
| Appraisal instruments | Consensus based Standards for the selection of health Measurements  Instruments (COSMIN) |

**Kang, J. 2016**

| Type of participants | Patients with chronic heart failure with anaemia. |
| --- | --- |
| Control group | Patients receiving placebo treatment. |
| Number of participants | 3172 |
| Description of interventions | EPO (various doses) 3 to 28 months. |
| Number of studies | 13 |
| Types of studies | Randomized controlled trials |
| Country of origin of studies | Not reported |
| Outcome assessed | Hb, NYHA grade for dyspnea, KOOQ scale and the MLHFQ scale. |
| Funding | Funded by Seoul National University Bundang Hospital Research Fund (grant no 14-2015-029). |
| Last search date | August 2015 |
| Language and publication restriction | No language restriction and published and unpublished articles were included. |
| Appraisal instruments | Cochrane Collaboration’s tool for assessing the risk of bias. |

**Lancaster, K. 2012**

| Type of participants | Elite and sub-elite athletes. |
| --- | --- |
| Control group | Athletes, normoxic groups. |
| Number of participants | 160 |
| Description of interventions | Natural and artificial LHTL altitude training studies. |
| Number of studies | 9 |
| Types of studies | Randomized controlled trials |
| Country of origin of studies | Australia, Austria, Finland, France, Japan & the United States. |
| Outcome assessed | VO_2_max, Hb, max Hr, Hematocrit and peak exercise blood lactate. |
| Funding | Not reported |
| Last search date | July 2010 |
| Language and publication restriction | No language restriction; only published studies. |
| Appraisal instruments | Own risk of bias score |

**Martí-Carjaval, A.J. 2013**

| Type of participants | Patients aged 16 years and older, with a diagnosis of rheumatoid arthritis and affected by anemia. |
| --- | --- |
| Control group | Patients receiving a placebo treatment |
| Number of participants | 133 |
| Description of interventions | Erythropoiesis-stimulating agents (epoetin (alpha or beta) or darbepoetin alpha) alone or in combination with oral or parenteral iron supplementation. |
| Number of studies | 3 |
| Types of studies | Randomized controlled trials |
| Country of origin of studies | Not reported |
| Outcome assessed | Hb and QoL |
| Funding | Funded by Iberoamerican Cochrane Centre, Spain and Cochrane Musculoskeletal Group, Canada. |
| Last search date | 7^th^ August 2012 |
| Language and publication restriction | No language and publication status restriction. |
| Appraisal instruments | Cochrane Collaboration’s tool for assessing the risk of bias. |

**Ngo, K. 2010**

| Type of participants | Heart failure patients |
| --- | --- |
| Control group | Placebo treatment |
| Number of participants | 794 |
| Description of interventions | ESA treatment |
| Number of studies | 11 |
| Types of studies | Randomized controlled trials |
| Country of origin of studies | Not reported |
| Outcome assessed | Exercise duration, 6-min walk distance, exercise tolerance, peak VO_2_, VO_2_ anaerobic threshold, change in Hb, NYHA functional class, Kansas Cardiomyopathy questionnaire and LVEF. |
| Funding | Clinical Trials and Evaluation Unit, Royal Brompton Hospital, UK. |
| Last search date | October 2008 |
| Language and publication restriction | No language restrictions. Only published studies were included. |
| Appraisal instruments | Cochrane risk of bias tool. |

**Nielsen, N.D. 2017**

| Type of participants | Critical ill patients in the Intensive care unit. |
| --- | --- |
| Control group | Patients not receiving transfusion or pre-transfusion. |
| Number of participants | 933 |
| Description of interventions | Red blood cell transfusion |
| Number of studies | 17 |
| Types of studies | Retrospective, prospective observational and randomized controlled studies. |
| Country of origin of studies | Switzerland, Brazil, Scotland, Belgium, France, USA, UK, Greece, Kenya, Austria, Italy and Uganda. |
| Outcome assessed | Tissue oxygenation pre and post transfusion. |
| Funding | None |
| Last search date | February 2017 |
| Language and publication restriction | No language or publication status restriction. |
| Appraisal instruments | Not reported |

**Palmer, S.C. 2014**

| Type of participants | Adults with chronic kidney disease. |
| --- | --- |
| Control group | Patients receiving placebo, epoetin alfa or beta, methoxy polyethylene glycol-epoetin beta. Other controls were made when comparing more frequent with less frequent darbepoetin alfa or intravenous with subcutaneous darbepoetin alfa. |
| Number of participants | 9414 |
| Description of interventions | Any darbepoetin alfa treatment of at least three months duration. |
| Number of studies | 32 |
| Types of studies | Randomized controlled trials |
| Country of origin of studies | Denmark, Japan, USA, Germany, Australia, France, Korea, China, Taiwan, Europa, Italy, UK and Spain. |
| Outcome assessed | Mean change in fact fatigue score, FS36, physical functioning score. |
| Funding | Not reported |
| Last search date | 13^th^ January 2014 |
| Language and publication restriction | No language restriction; Publication status restriction is not reported. |
| Appraisal instruments | Cochrane risk of bias tool |

**Park, S. 2016**

| Type of participants | Patients with myelodysplastic syndrome. |
| --- | --- |
| Control group | Patients with MS receiving less EPO. |
| Number of participants | 647 |
| Description of interventions | 60-500mcg EPO |
| Number of studies | 10 |
| Types of studies | Phase II studies and one randomized controlled trial. |
| Country of origin of studies | Not reported |
| Outcome assessed | QoL and Hb |
| Funding | Funded by Amgen. |
| Last search date | August 2015 |
| Language and publication restriction | No language restriction; only published articles were included. |
| Appraisal instruments | Not reported |

**Pasricha, S.R. 2014**

| Type of participants | Woman of reproductive age. |
| --- | --- |
| Control group | Patients receiving no iron. |
| Number of participants | 911 |
| Description of interventions | Oral iron supplementation daily (at least 5 days a week). |
| Number of studies | 22 |
| Types of studies | Randomized controlled trials |
| Country of origin of studies | Mexico, USA, Finland, Germany, China, Israel, Serbia, Canada, Japan, Switzerland and Australia. |
| Outcome assessed | *Max exercise*: VO_2_ max, Heart rate, Time to exhaustion; *submaximal exercise*: Heart rate, %VO_2_max, time to exhaustion, energy consumption. |
| Funding | a Victoria Fellowship by the Government of Victoria, a CRB Blackburn Scholarship by the Royal Australasian College of Physicians, an Overseas Research Experience Scholarship by the University of Melbourne, and a National Health and Medical Research Council CJ Martin Early Career Fellowship; unrestricted research grant from Vifor Pharma. |
| Last search date | 28^th^ July 2013 |
| Language and publication restriction | No language restrictions; publication status restrictions are not reported. |
| Appraisal instruments | Cochrane Collaboration’s tool for assessing the risk of bias |

**Pergola, P.E. 2019**

| Type of participants | Non dialysis dependent chronic kidney disease patients with anaemia. |
| --- | --- |
| Control group | Placebo or lower Hb-target. |
| Number of participants | 37105 |
| Description of interventions | Various amounts of erythropoiesis stimulating agents. |
| Number of studies | 16 |
| Types of studies | Retrospective, prospective, simulation study, randomized controlled trials and cross-sectional studies. |
| Country of origin of studies | North-America, Europe and elsewhere. |
| Outcome assessed | QoL |
| Funding | Funded by Akebia Therapeutics. |
| Last search date | 17^th^ March 2017 |
| Language and publication restriction | :anguage restrictions were not reported; only published studies were included. |
| Appraisal instruments | Not reported |

**Pinchon, D.J. 2009**

| Type of participants | Patients with myelodysplastic syndromes |
| --- | --- |
| Control group | Supportive care or none |
| Number of participants | 1234 |
| Description of interventions | Red blood cell transfusion |
| Number of studies | 17 |
| Types of studies | Observational studies, surveys and randomized controlled studies. |
| Country of origin of studies | Not reported |
| Outcome assessed | Quality of life |
| Funding | Not reported |
| Last search date | February 2009 |
| Language and publication restriction | No language or publication restrictions. |
| Appraisal instruments | None |

**Ross, S.D. 2003**

| Type of participants | Patients with renal insufficiency- or cancer-related anaemia. |
| --- | --- |
| Control group | No EPO treatment |
| Number of participants | 12948 |
| Description of interventions | EPO treatment |
| Number of studies | 32 |
| Types of studies | Prospective interventional studies |
| Country of origin of studies | Not reported |
| Outcome assessed | Overall, fatigue/energy, physical, activity, performance score. |
| Funding | Not reported |
| Last search date | 31 December 2001 |
| Language and publication restriction | Language unknown. Only published studies are included. |
| Appraisal instruments | None |

**Saunders, P.U. 2013**

| Type of participants | Elite endurance athletes. |
| --- | --- |
| Control group | The data from control participants and from altitude protocols that would not normally be expected to induce an increase in Hb-mass (IHE and IHT alone). |
| Number of participants | 145 |
| Description of interventions | Altitude training. |
| Number of studies | 10 |
| Types of studies | Not reported |
| Country of origin of studies | Not reported |
| Outcome assessed | Hb-mass and VO_2_max. |
| Funding | Not reported |
| Last search date | Not reported |
| Language and publication restriction | Not reported |
| Appraisal instruments | None |

**Sgrò, P. 2018**

| Type of participants | Endurance athletes receiving EPO. |
| --- | --- |
| Control group | No EPO. |
| Number of participants | 165 |
| Description of interventions | Different types of EPO. |
| Number of studies | 11 |
| Types of studies | Non-randomized trials and randomized controlled trials. |
| Country of origin of studies | Not reported |
| Outcome assessed | Hb-mass, HCT, VO_2_max and max power. |
| Funding | None |
| Last search date | 14^th^ February 2016 |
| Language and publication restriction | Only English, French and Italian published papers were included. |
| Appraisal instruments | JADA-5 points |

**Solheim, S.A. 2019**

| Type of participants | Adults, untrained and well-trained. |
| --- | --- |
| Control group | No control or receiving no transfusion. |
| Number of participants | 255 |
| Description of interventions | Various amounts of autologous red cell transfusions. |
| Number of studies | 28 |
| Types of studies | Randomized and non-randomized controlled trials. |
| Country of origin of studies | Not reported |
| Outcome assessed | VO_2_max and change in performance test. |
| Funding | Funded by the anti-doping Denmark, Partnership for Clean Competition and World anti-doping agency. |
| Last search date | January 2019 |
| Language and publication restriction | Only full-text publications; Language restrictions not reported. |
| Appraisal instruments | None |

**Staibano, P. 2020**

| Type of participants | Patients with renal disease of any age. |
| --- | --- |
| Control group | Oral iron, no intervention, placebo, rHuEPO. |
| Number of participants | 11,637 |
| Description of interventions | Iron intravenous, Darbepoetin alpha, epoetin alfa, rHuEPO in various doses. |
| Number of studies | 21 |
| Types of studies | Randomized controlled trials, prospective cohorts and single arm studies. |
| Country of origin of studies | Not reported |
| Outcome assessed | QoL: SF-36 and KDQ. |
| Funding | Funded by the Hematology Opportunities for the Next Generation of Research Scientists (HONORS) award offered by the American Society of Hematology. |
| Last search date | March 2018 |
| Language and publication restriction | Only published studies written in English; publication status not reported. |
| Appraisal instruments | Cochrane risk of bias tool |

**Van Remoortel, H. 2017**

| Type of participants | Healthy adults performing aerobic exercise at a competitive or non-competitive level were included. |
| --- | --- |
| Control group | No blood donation, followed by exercise of light, moderate, vigorous, or maximal intensity. |
| Number of participants | 187 |
| Description of interventions | A standard whole blood donation (i.e., 400-500 mL), followed by aerobic exercise of light, moderate, vigorous, or maximal intensity was included. We excluded interventions with blood loss or blood donations of more than 500 ml. Blood reinfusion interventions or other interventions aimed at replacing the blood volume were also excluded. |
| Number of studies | 18 |
| Types of studies | Case-crossover studies. |
| Country of origin of studies | USA, UK, Sweden, Germany, Poland, Canada, Spain and Norway. |
| Outcome assessed | VO_2_max, Time till exhaustion, maximal power output and Hb. |
| Funding | Funded by Belgian Red Cross– Flanders. |
| Last search date | November 2013 |
| Language and publication restriction | Published articles written in English, German, French and Dutch. |
| Appraisal instruments | GRADE |

# **Supplementary material 2:** Excluded studies with reason for exclusion

| **Study** | **Reason for exclusion** |
| --- | --- |
| Aapro 2012^1^ | No physiological or QoL outcomes |
| Anand 2018^2^ | Not a systematic review and/or meta-analysis |
| Bailey 1997^3^ | Not a systematic review and/or meta-analysis |
| Balsalobre 2014^4^ | Not a systematic review and/or meta-analysis |
| Baranauskas 2021^5^ | Not a systematic review and/or meta-analysis |
| Bazeley 2014^6^ | Not a systematic review and/or meta-analysis |
| Bennett 2005^7^ | Not a systematic review and/or meta-analysis |
| Bukmir 2016^8^ | Not a systematic review and/or meta-analysis |
| Cacic 2013^9^ | Not a systematic review and/or meta-analysis |
| Carson 2021^10^ | No physiological or QoL outcomes |
| Castelli 2018^11^ | Not a systematic review and/or meta-analysis |
| Chapman 2014^12^ | Not a systematic review and/or meta-analysis |
| Chen 2013^13^ | Trial, not a review |
| Cingam^14^ | In review process: too much uncertainty over relation of outcomes to Hb |
| De Franceschi 2017^15^ | No physiological or QoL outcomes and no Hb-levels reported |
| De las Cuevas Allende 2021^16^ | No physiological or QoL outcomes |
| De Paula 2010^17^ | Manuscript of a presentation at a conference |
| Dempsey 2015^18^ | No Hb-levels reported |
| Eichner 2012^19^ | Not a systematic review and/or meta-analysis |
| Ershler 2019^20^ | No physiological or QoL outcomes and no Hb-levels reported |
| Faiss 2013^21^ | No Hb-levels reported |
| Ferguson 2015^22^ | Outcomes: Cost/QALY |
| Flaherty 2016^23^ | Not a systematic review and/or meta-analysis |
| Germing 2019^24^ | Not a systematic review and/or meta-analysis |
| Gomez Ramirez 2017^25^ | Focusses on treatment choice; no physiological or QoL outcomes |
| Guin 2020^26^ | Outcomes: Mortality/Time to Death; no physiological or QoL outcomes |
| Hanna 2021^27^ | Outcomes: Burden of disease; not effect of intervention |
| Hebert 1997^28^ | Combines animal and human studies |
| Hellstrom 2013^29^ | Not a systematic review and/or meta-analysis |
| Jacobs 2012^30^ | Critically low score in the JBI appraisal tool |
| Jing 2012^31^ | Outcomes: Mortality; Myocardial infarction; no physiological or QoL outcomes |
| Jones 2004 (2)^32^ | No hematological outcomes reported |
| Kiladjian 2015^33^ | No physiological or QoL outcomes and no Hb-levels reported |
| Kiliçgedik 2011^34^ | Not a systematic review and/or meta-analysis |
| Kimel 2008^35^ | Not a systematic review and/or meta-analysis |
| Kotecha 2011^36^ | Article based on Cochrane review by Ngo et al. |
| Krantz 1995^37^ | Not a systematic review and/or meta-analysis |
| Krishnan 2022^38^ | Not a systematic review and/or meta-analysis |
| Leaf 2009^39^ | Not a systematic review and/or meta-analysis |
| Mies 2013^40^ | Not a systematic review and/or meta-analysis |
| Montero 2018^41^ | Not a systematic review and/or meta-analysis |
| Oliva 2012^42^ | Not a systematic review and/or meta-analysis |
| Otto 2013^43^ | Not a systematic review and/or meta-analysis |
| Palmer 2014-2^44^ | Reported no relevant outcomes due to insufficient evidence |
| Pinchon 2009^45^ | Reported no relevant outcomes due to insufficient evidence |
| Ploszczyca 2018^46^ | Hematological response reported; no physiological or QoL outcomes |
| Roubinian 2016^47^ | Outcomes were morbidity and mortality; no physiological or QoL outcomes |
| Seastone 2016^48^ | Hematological response and toxicity, no physiological or QoL outcomes |
| Shah 2018^49^ | Not a systematic review and/or meta-analysis |
| Simon 2019^50^ | Model; not a systematic review and/or meta-analysis |
| Silverberg 2010^51^ | Not a systematic review and/or meta-analysis |
| Staibano 2018^52^ | Scoping review |
| Stein 2014^53^ | Reports on disease burden; not on intervention |
| Stickel 2012^54^ | Not a systematic review and/or meta-analysis |
| Strauss 2018^55^ | Not a systematic review and/or meta-analysis |
| Strzała 2011^56^ | Not a systematic review and/or meta-analysis |
| Wagner 2015^57^ | Cross-sectional measures; no change in Hb reported |
| Wehrlin 2016^58^ | Not a systematic review and/or meta-analysis |
| Wilde 2019^59^ | No physiological or QoL outcomes |
| Zelenkova 2019^60^ | Prospective study; not a review |

# **Supplementary material 3:** Outcomes per comparison

##
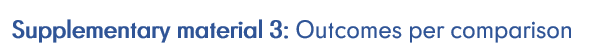
**Comparison 1.** ESAs vs placebo, lower target, or no ESAs

|  | **Patient group** | **Outcome** | | **Study** | **No. of participants** | **Statistical method** | **Effect size** | **Hematological outcome** | **Significance direction** | **Quality of evidence** (GRADE) |
| --- | --- | --- | --- | --- | --- | --- | --- | --- | --- | --- |
| **Anaemia** | **Rheumatoid arthritis** | QoL | | Martí-Carvajal 2013 | 133 | Qualitative synthesis | *‘’They* [the eligible RCTs] *provide evidence of benefit in improving health-related quality of life and in increasing hemoglobin levels.’’* | No pooled data; 2/3 studies found significant increase in Hb in EPO compared to placebo | Favours ESAs^#^ | ⴲⵔⵔⵔ |
|  | **CKD** – Dialysis | Fatigue | | Johansen 2012 | 3 082 | Qualitative synthesis;  % Increase in PRO-scores | 2.3-68.2% PRO increase | No pooled data; strongest increase in PRO outcome in Hb baseline <10 g/dL treated to Hb≥10 g/dL (≥1 g/dL change) | Favours ESAs^#^ | ⴲⴲⵔⵔ |
|  |  | VO_2_max | | Johansen 2010 | 762 | % difference (IV, Random, 95% CI) | 23.8% [18.56, 28.97] | Average increase in Hb was 58% | Favours ESAs | ⴲⴲⵔⵔ |
|  |  | QoL - KPS | |  | 629 | % Change (IV, Random, 95% CI) | 10.5% [4.74, 16.32] | Average increase in Hb was 58% | Favours ESAs | ⴲⴲⵔⵔ |
|  | **CKD** – Predialysis | QoL | | Pergola 2019 | 37 105 | Qualitative synthesis | *“In general, HRQoL benefits associated with higher hemoglobin*  *targets appeared modest and of uncertain clinical significance”* | No pooled data; Variable definitions of high and low Hb targets (or placebo) used | Favours high target^#^ | ⴲⵔⵔⵔ |
|  |  |  |  | Cody 2016 | 14 | Mean Difference (IV, Random, 95% CI) | -35 [-57.53, -12.47] | MD: Hb -1.9 g/dL [-2.34, -1.47]  MD: Hct -9.85% [-11.34, -8.35] | Favours ESAs* | ⴲⵔⵔⵔ |
|  |  |  |  | Gandra 2010 | 5010 | Qualitative synthesis | *‘’RCTs and single-arm studies indicate that treatment of anemia with ESAs improves energy and physical function in nondialysis CKD patients.’’* | Starting Hb: 8.8-11.9 g/dL  Study end Hb: 8.6-13.9 g/dL | Favours ESAs | ⴲⴲⵔⵔ |
|  |  | Exercise capacity | | Cody 2016 | 22 | Mean Difference (IV, Random, 95% CI) | -46 [-90.73, -1.27] | MD: Hb -1.9 g/dL [-2.34, -1.47]  MD: Hct -9.85% [-11.34, -8.35] | Favours ESAs* | ⴲⵔⵔⵔ |
|  | **CKD** – unselected | QoL |  | Staibano 2020 | 11 637 | Qualitative synthesis | *“We found no significant association*  *between improvement in hematological outcomes*  *and improvement in any of the 5 QOL domains”* | Improvement in hematological outcome *yes/no* | No significant difference^#^ | ⴲⵔⵔⵔ |
|  |  |  | SF - Physical function | Collister 2016 | 8292 | Mean Difference (IV, Random, 95% CI) | −2.6 (−5.6, 0.4) | Low target: 7.4 to 12 g/L  High target: 10.2 to 13.6 g/L | No significant difference | ⴲⵔⵔⵔ |
|  |  |  | SF - Physical role |  | 8292 |  | −0.7 (−5.1, 3.7) |  |  |  |
|  |  |  | SF - General health |  | 7229 |  | −1.9 (−4.1, 0.4) |  |  |  |
|  |  |  | SF - Vitality |  | 8792 |  | −2.0 (−4.4, 0.4) |  |  |  |
|  |  |  | SF - Social functioning |  | 8209 |  | −0.8 (−3.7,2.0) |  |  |  |
|  |  |  | SF - Emotional role |  | 7606 |  | 0.3 (−3.2, 3.8) |  |  |  |
|  |  |  | SF - Mental role |  | 7613 |  | −0.8 (−2.5, 0.9) |  |  |  |
|  |  |  | KDQ - Physical |  | 638 | Mean Difference (IV, Random, 95% CI) | 0.5 (-2.2, 1.2) |  |  |  |
|  |  |  | SF - Physical functioning | Guedes 2020 | 7322 | Std. Mean Difference (IV, Random, 95% CI) | 0.08 (−0.03,0.19) | Low target: Hb 10–11.5 g/dL | No significant difference  𐤏 | ⴲⵔⵔⵔ |
|  |  |  | SF - Physical role |  | 7322 |  | 0.09 (0.00, 0.18) | High target: Hb > 11.5 g/dL |  |  |
|  |  |  | SF - Physical functioning | Palmer 2014-1 | 3531 | Mean Difference (IV, Random, 95% CI) | 0.2 [-0.39, 0.79] | Starting Hb: <11.0 g/dL; Target: 13.0 g/dL | No significant difference* | ⴲⴲⵔⵔ |
|  |  |  | SF - energy: |  |  |  | 0.5 [-0.15, 1.15] |  |  |  |
|  |  |  | KPS | Jones 2004 | 201 | Mean change (IV, Random 95% CI) | 8.46 (2.01, 14.92) | Hb change: 3.37 g/dL (2.82, 3.92) | Favours ESAs | ⴲⵔⵔⵔ |
|  |  |  | KDQ - Physical |  | 164 |  | 1.20 [0.88, 1.52) |  |  | ⴲⴲⴲⵔ |
|  |  |  | KDQ -Relationships |  | 164 |  | 0.45 (0.19, 0.71) |  |  | ⴲⴲⴲⵔ |
|  |  |  | KDQ Depression |  | 164 |  | 0.40 (0.10, 0.70) |  |  | ⴲⴲⴲⵔ |
|  |  |  | KDQ Frustration |  | 164 |  | 0.19 (-0.12, 0.49) |  |  | ⴲⴲⴲⵔ |
|  |  |  | KDQ Global physical |  | 81 |  | 0.65 (0.31, 0.99) |  |  | ⴲⴲⴲⵔ |
|  |  |  | KDQ Global emotional |  | 81 |  | 0.29 (-0.04, 0.63) |  |  | ⴲⴲⴲⵔ |
|  |  |  | SIP Global |  | 239 |  | −6.55  (-4.10, -5.24) |  |  | ⴲⴲⴲⵔ |
|  |  |  | SIP Physical |  | 239 |  | −3.85  (-4.24, -3.46) |  |  | ⴲⴲⴲⵔ |
|  |  |  | SIP Psychological |  | 239 |  | −8.85 (-9.93, -7.77) |  |  | ⴲⴲⴲⵔ |
|  |  | QoL | SF - Physical functioning | Clement 2009 | 3027 | Mean Difference (IV, Random, 95% CI) | 2.9 [1.3 to 4.5] | High target: 11.5-16.0 g/dL  Low target: 9.0-12.0 g/dL | Favours ESAs – targeting >12 g/dL leads to small and not clinically meaningful improvements in HQOL | ⴲⴲⴲⵔ |
|  |  |  | SF - physical role |  | 3027 |  | 0.3 (−3.2 to 3.8) |  |  | ⴲⵔⵔⵔ |
|  |  |  | SF - General health |  | 3197 |  | 2.7 (1.3 to 4.2) |  |  | ⴲⴲⴲⵔ |
|  |  |  | SF – Vitality |  | 3614 |  | 3.2 (1.9 to 4.4) |  |  | ⴲⴲⴲⴲ |
|  |  |  | SF - Social function |  | 3027 |  | 1.3 (−0.8 to 3.4) |  |  | ⴲⴲⵔⵔ |
|  |  |  | SF - Emotional role |  | 2424 |  | −3.0 (−7.4 to 1.4) |  |  | ⴲⴲⵔⵔ |
|  |  |  | SF - Mental health |  | 3027 |  | 0.4 (0.1 to 0.8) |  |  | ⴲⴲⵔⵔ |
|  |  | Fatigue | FACT - Fatigue | Palmer 2014-1 | 3531 | Mean Difference (IV, Random, 95% CI) | 1.4 [0.71, 2.09] | Starting Hb: <11.0 g/dL; Target: 13.0 g/dL | Favours high target* | ⴲⴲⴲⵔ |
|  |  |  | KDQ | Collister 2016^61^ | 638 | Mean Difference (IV, Random, 95% CI) | 0.5 (-1.6,0.5) | Low target: 7.4 to 12 g/L  High target: 10.2 to 13.6 g/L | No significant difference | ⴲⵔⵔⵔ |
|  |  |  |  | Guedes 2020 | 8 310 | Std. Mean Difference (IV, Random, 95% CI) | 0.16 (0.09-0.24) | Low target: Hb 10–11.5 g/dL  High target: Hb > 11.5 g/dL | Favours high target | ⴲⴲⵔⵔ |
|  |  |  |  | Jones 2004 | 164 | Mean change (IV, Random 95% CI) | 0.79 (0.49–1.09) | Hb change: 3.56 g/dL (2.94, 4.17) | Favours ESAs | ⴲⴲⴲⵔ |
|  | **CHF** | QoL | NYHA functional class improvement | Ngo 2010 | 657 | Mean Difference (IV, Random, 95% CI) | -0.73 [-1.11, -0.36] | Change in Hb level 1.98 g/dL [1.62, 2.35] | Favours ESAs | ⴲⴲⵔⵔ |
|  |  |  | Kansas City cardiomyopathy questionnaire |  | 247 | Mean Difference (IV, Fixed, 95% CI) | 4.60 [0.46, 8.75] |  |  | ⴲⴲⵔⵔ |
|  |  |  | Minnesota living with Heart failure questionnaire |  | 462 | Mean Difference (IV, Random, 95% CI) | -2.02 [-5.78, 1.73] |  |  | ⴲⴲⵔⵔ |
|  |  |  | Patient’s global assessment |  | 548 | Risk Ratio (M-H, Fixed, 95% CI) | 1.16 [1.02, 1.32] |  |  | ⴲⴲⵔⵔ |
|  |  |  | Kansas City cardiomyopathy questionnaire | Kang 2016 | 2488 | Std. Mean Difference (IV, Random, 95% CI) | 1.70(-0.01, 3.41) | Hb increase 2.13 mg/dL [2.60,1.67) | Not significant | ⴲⴲⵔⵔ |
|  |  |  | Minnesota living with Heart failure questionnaire |  | 266 |  | -0.82 [-1.47, -0.18] |  | Favours ESAs | ⴲⴲⴲⵔ |
|  |  | NYHA grade for dyspnea | |  | 427 | Std. Mean Difference (IV, Random, 95% CI) | -1.63 (-2.62, -0.65) |  | Favours ESAs | ⴲⴲⴲⵔ |
|  |  | VO_2_max | | Ngo 2010 | 102 | Mean difference (IV, fixed, 95% CI) | 2.29 [0.62, 3.95] | Change in Hb level 1.98 g/dL [1.62, 2.35] | Favours ESAs | ⴲⴲⵔⵔ |
|  |  | VO_2_ anaerobic threshold | | Ngo 2010 | 61 | Mean difference (IV, fixed, 95% CI) | 2.92 [0.09, 5.75] | Change in Hb level 1.98 g/dL [1.62, 2.35] | Favours ESAs | ⴲⴲⵔⵔ |
|  |  | Exercise capacity | Exercise duration | Ngo 2010 | 362 | Mean difference (IV, Random, 95% CI) | 96.82 [5.22, 188.42] | Change in Hb level 1.98 g/dL [1.62, 2.35] | Favours ESAs | ⴲⴲⴲⵔ |
|  |  |  | 6-min walk test |  | 261 |  | 69.33 [16.99, 121.  67] |  |  | ⴲⴲⴲⵔ |
|  |  | LVEF% | | Ngo 2010 | 321 | Mean Difference (IV, Random, 95% CI) | 5.77 [2.43, 9.11] | Change in Hb level 1.98 g/dL [1.62, 2.35] | Favours ESAs | ⴲⴲⴲⵔ |
|  | **Cancer** | QoL | FACT-F | Bohlius 2014 | 6108 | Mean Difference (IV, Random, 95% CI) | 0.22 (0.13, 0.32) | Baseline Hb: median 10.1  Target Hb: ≤13-15 g/dL | Favours ESAs | ⴲⴲⵔⵔ |
|  |  |  | FACT-An |  | 2765 |  | 0.30 (0.17, 0.42) |  |  |  |
|  |  |  | FACT-G |  | unclear |  | 1.45 (0.02, 2.88) |  |  |  |
|  | **CKD/Cancer** | QoL | Overall | Ross 2003 | 5636 | Mean correlation with change in Hct (Random, 95% CI) | 0.23 (0.18 to 0.28) | Correlation with Hct | Favours ESA | ⴲⵔⵔⵔ |
|  |  |  | Energy/fatigue |  | 1716 |  | 0.30 (0.25 to 0.35) |  |  |  |
|  |  |  | Physical |  | 134 |  | 0.33 (0.16 to 0.47) |  |  |  |
|  |  |  | Activity |  | 1582 |  | 0.28 (0.23 to 0.33) |  |  |  |
|  |  |  | Performance score |  | 912 |  | 0.21 (0.12 to 0.29) |  |  |  |
|  | **MDS** | QoL | | Park 2016 | 647 | Qualitative synthesis | *‘’…most studies reporting that responders showed significant improvement in QoL’’* | Responder to ESAs *yes/no* | Favours higher Hb | ⴲⵔⵔⵔ |
|  |  |  |  | Pinchon 2009 | 1234 | Qualitative synthesis | *‘’Relevant QoL improvement from baseline to final evaluation (..) in erythroid responders, no change in non-responders ‘’* | Responder to ESAs *yes/no* | Favours ESAs | ⴲⵔⵔⵔ |
| **Non anaemia** | **Untrained and trained adults** | VO_2_max | | Heuberger 2013^62^ | 165 | Qualitative synthesis;  % Increase in parameters | *‘’this parameter (VO_2_max) is increased in the*  *rHuEPO-treated subjects, with a relatively constant value*  *for all studies, independent of the training status of the*  *subjects, between 7 and 9.7%’’* | Hb: 4.6-17.4% increase;  Hct: 8.3-16.4% increase | Favours ESAs | ⴲⵔⵔⵔ |
|  | **Athletes** | VO_2_max | | Sgrò 2018^63^ | 148 | Qualitative synthesis | *‘’Evidence coming empirical trials showed a consistent and positive*  *effects of rHuEpo on VO_2_max of about 6-8% or 300-400 ml O_2_/min,*  *if expressed in absolute terms, when moderate doses of rHuEpo are*  *administered.’’* | No pooled data;  Hb increase:  14.0-15.8 to  15.9-17.4 g/dl;  Hct increase:  42.7%-45.7% to 48.1%-51.0%;  Hb-mass:  12.7 to 15.2 g/kg | Favours ESAs | ⴲⵔⵔⵔ |
|  |  | Exercise capacity | |  | 72 |  | *‘’In cycling, this improvement may be translated in an*  *increase of 25-35 watts expressed at maximum aerobic power. Also in*  *running the improvement of about 6 % in 3k time trial has been*  *observed’’* |  | Favours ESAs | ⴲⵔⵔⵔ |

*One study only
^#^ In case of a qualitative synthesis, when no meta-analysis was performed, the significance direction represents the authors conclusion.

## **Comparison 2.** Iron vs placebo, Iron IV vs oral, or no iron

|  | **Patient group** | **Outcome** | | **Study** | **No. of participants** | **Statistical method** | **Effect size** | **Hematological outcome** | **Significance direction** | **Quality of evidence** (GRADE) |
| --- | --- | --- | --- | --- | --- | --- | --- | --- | --- | --- |
| **Anaemia** | **Adults without CKD** | QoL | | Gurusamy 2013 | Oral: 851 | Std. Mean Difference [IV, Random, 95% CI] | 0.13  [-0.10,0.37] | Hb increase 0.91 g/dL [0.48,1.35] | No significant difference | ⴲⴲⵔⵔ |
|  |  |  |  |  | IV: 1629 | No pooled data | The point estimate of hemoglobin in intervention groups in  the individual studies was between  - 0.04 SD and 0.44 SD | Hb increase: 2.5 g/dL [2.33,2.67] | No significant difference |  |
|  |  |  |  | Clevenger 2016 | Oral: 851 | Mean Difference [IV, Random, 95% CI] | 0.13  [-0.10,0.37] | Hb increase 0.91 g/dL [0.48,1.35] | No significant difference | ⴲⴲⵔⵔ |
|  |  |  |  |  | IV: 1744 | Mean Difference [IV, Random, 95% CI] | -0.22  [-0.45,0.00] | Hb increase MD 1.04 g/dL [0.52,1.57] | Favours parental iron | ⴲⵔⵔⵔ |
| **Anaemia and non-anaemia** | **Woman of reproductive age** | VO_2_max | | Pasricha 2014 | Maximal exercise: 620 | Mean Difference [IV, Random, 95% CI] | 2.35 [0.82, 3.88] | Hb increase:  6.79g/L [4.29, 9.30] | Favours iron | ⴲⴲⵔⵔ |
|  |  |  |  |  | Submaximal exercise: 316 |  | -2.68 [-4.94, -0.41] |  |  |  |
|  |  | Exercise capacity  (Time to exhaustion) | |  | Max: 49 |  | 1.09 [-0.48, 2.66] |  | No significant difference | ⴲⴲⵔⵔ |
|  |  |  |  |  | Submax: 52 |  | 1.23 [-0.41, 2.87] |  |  |  |
|  |  | Max heart rate | |  | Max: 152 |  | 1.27 [-1.08, 3.61] |  | Favours iron | ⴲⴲⴲⵔ |
|  |  |  |  |  | Submax: 172 |  | -4.05 [-7.25,  -0.85] |  |  |  |
| **Non-anaemia** | **IDNA athletes** | VO_2_max | | Burden 2015 | 443 | Hedges G [IV, Fixed, 95% CI] | 0.610 [0.399,0.821] | Hb increase: 0.695 [0.534-0.856] (Hedges G) | Favours iron | ⴲⴲⵔⵔ |
|  | **IDNA general** | VO_2_max | | Houston 2018 | 235 | Std. Mean Difference [IV, Random, 95% CI] | 0.11 [-0.15, 0.37] | Hb increase: 4.01 g/L [1.22 to 6.81] | No significant effect | ⴲⴲⵔⵔ |
|  |  | Exercise capacity | 15km time trial |  | 79 | Std. Mean Difference [IV, Random, 95% CI] | -0.09 [-0.53,0.35] |  | No significant effect | ⴲⵔⵔⵔ |
|  |  |  | Time to exhaustion |  | 69 |  | 0.25 [-0.22, 0.73] |  | No significant effect | ⴲⴲⵔⵔ |
|  |  | Fatigue | |  | 714 | Std. Mean Difference [IV, Random, 95% CI] | -0.38[-0.52, -0.23] |  | Favours iron | ⴲⴲⴲⵔ |

## **Comparison 3.** Pre-transfusion vs. post-transfusion

|  | **Patient group** | **Outcome** | **Study** | **No. of participants** | **Statistical method** | **Effect size** | **Hematological outcome** | **Significance direction** | **Quality of evidence** (GRADE) |
| --- | --- | --- | --- | --- | --- | --- | --- | --- | --- |
| **Anaemia** | **Critically ill ICU patients** | Tissue oxygenation | Nielsen 2017 | 933 | Qualitative synthesis | *‘’RBC transfusion fails to result in significant improvements in either tissue oxygenation or microcirculatory flow in ICU patients’’* | No pooled data; Various pre-transfusion Hb’s per study reported | No significant effect^#^ | ⴲⵔⵔⵔ |
| **Non-anaemia** | **Autologous transfusion in untrained and trained adults** | VO_2_max | Solheim 2019 | 255 | Qualitative synthesis | *‘’…reinfusion with as little as 135 mL packed*  *RBCs increases time-trial performance. Red blood cell*  *reinfusion increases endurance performance by elevating*  *CaO_2_.’’* | No pooled data; Per study, an increase in Hb/Hct is reported, depending on the amount of red blood cells transfused. | Favours transfusion | ⴲⵔⵔⵔ |
|  |  | Exercise capacity |  |  |  | *‘’Apparently, the magnitude of change in hemoglobin*  *concentration explains the increase in VO_2_peak associated*  *with ABT...’’* |  | Favours transfusion | ⴲⵔⵔⵔ |

## **Comparison 4.** Restrictive transfusion threshold vs. liberal threshold

|  | **Patient group** | **Outcome** | **Study** | **No. of participants** | **Statistical method** | **Effect size** | **Hematological outcome** | **Significance direction** | **Quality of evidence** (GRADE) |
| --- | --- | --- | --- | --- | --- | --- | --- | --- | --- |
| **Anaemia** | **Hematological malignancies under treatment** | Fatigue score | Estcourt 2017 | 89 | Qualitative synthesis  Median (IQR) | Restrictive: 4.8 [4,5.2];  Liberal:  4.5 [3.6,5] * | Restrictive Hb: 70-90 g/L  Liberal Hb: 100-120 g/L | No significant effect^#^ | ⴲⵔⵔⵔ |
|  | **Chronic bone marrow failure** | Activity | Gu 2015 | 13 | Qualitative synthesis | No reported effect on activity (no statistics provided) * | Restrictive trigger: < 72 g/L,  Liberal trigger: < 96 g/L, | No significant effect^#^ | ⴲⵔⵔⵔ |

## **Comparison 5.** Altitude training vs. no altitude training

|  | **Patient group** | **Outcome** | **Study** | **No. of participants** | **Statistical method** | **Effect size** | **Hematological outcome** | **Significance direction** | **Quality of evidence** (GRADE) |
| --- | --- | --- | --- | --- | --- | --- | --- | --- | --- |
| **Non-anaemia** | **Elite athletes** | Maximal heart rate | Lancaster 2012 | 114 | Mean Difference [IV, Random, 95% CI] | -1.77 [-3.03, -0.50] | Hb increase:  0.57 [0.38, 0.75] | Favours hypoxia | ⴲⴲⵔⵔ |
|  |  | VO_2_max |  | 162 | Mean Difference [IV, Random, 95% CI] | 1.51 [0.44, 2.58] | Hct MD: 0.60 {-0.69,1.88] | Favours hypoxia | ⴲⴲⵔⵔ |
|  | **Elite and sub-elite athletes** |  | Saunders 2013 | 145 | Linear regression: Hb-mass and VO_2max_ | Slope: 0.48 [0.30, 0.67] | Hb increase: ∼3% | Favours hypoxia | ⴲⴲⵔⵔ |

## **Comparison 6.** Before blood donation vs. post-donation

|  | **Patient group** | **Outcome** | | **Study** | **No. of participants** | **Statistical method** | **Effect size** | **Hematological outcome** | **Significance direction** | **Quality of evidence** (GRADE) |
| --- | --- | --- | --- | --- | --- | --- | --- | --- | --- | --- |
| **Non-anaemia** | **Healthy adults** | VO_2_max | | Van Remoortel 2017 | 83 | Mean Difference [IV, Random, 95% CI] | -3.5 [-5.3, -1.7] | Hb increase: --7% [-10%, -4%] | Favours pre-donation | ⴲⴲⵔⵔ |
|  |  |  |  | Johnson 2019 | 47 | Cohen's d [95% CI] | -0.26 [-1.46, 0.94] | Hb increase: -0.75 [-2.04, 0.54] | No significant effect | ⴲⵔⵔⵔ |
|  |  | Exercise capacity | Time To Exhaustion | Van Remoortel 2017 | 37 | Mean Difference [IV, Random, 95% CI] | -51 [-91, -11] | Hb increase: ---7% [-10%, -4%] | Favours pre-donation | ⴲⵔⵔⵔ |
|  |  |  | Max power |  |  |  | -15[-34, 4] |  |  | ⴲⵔⵔⵔ |
|  |  |  | Time To Exhaustion | Johnson 2019 | 71 | Cohen's d [95% CI] | -0.88 [-37.29, 35.52] | Hb increase: -0.75[-2.04, 0.54]  HCT:  -1.16 [-4.59, 2.28]  RBCs:  -4.23[-4.37, -4.10] | No significant effect | ⴲⵔⵔⵔ |
|  |  | Maximal heart rate | |  | 63 | Cohen's d [95% CI] | -0.08 [-16.73, 16.56] |  | No significant effect | ⴲⵔⵔⵔ |

# **
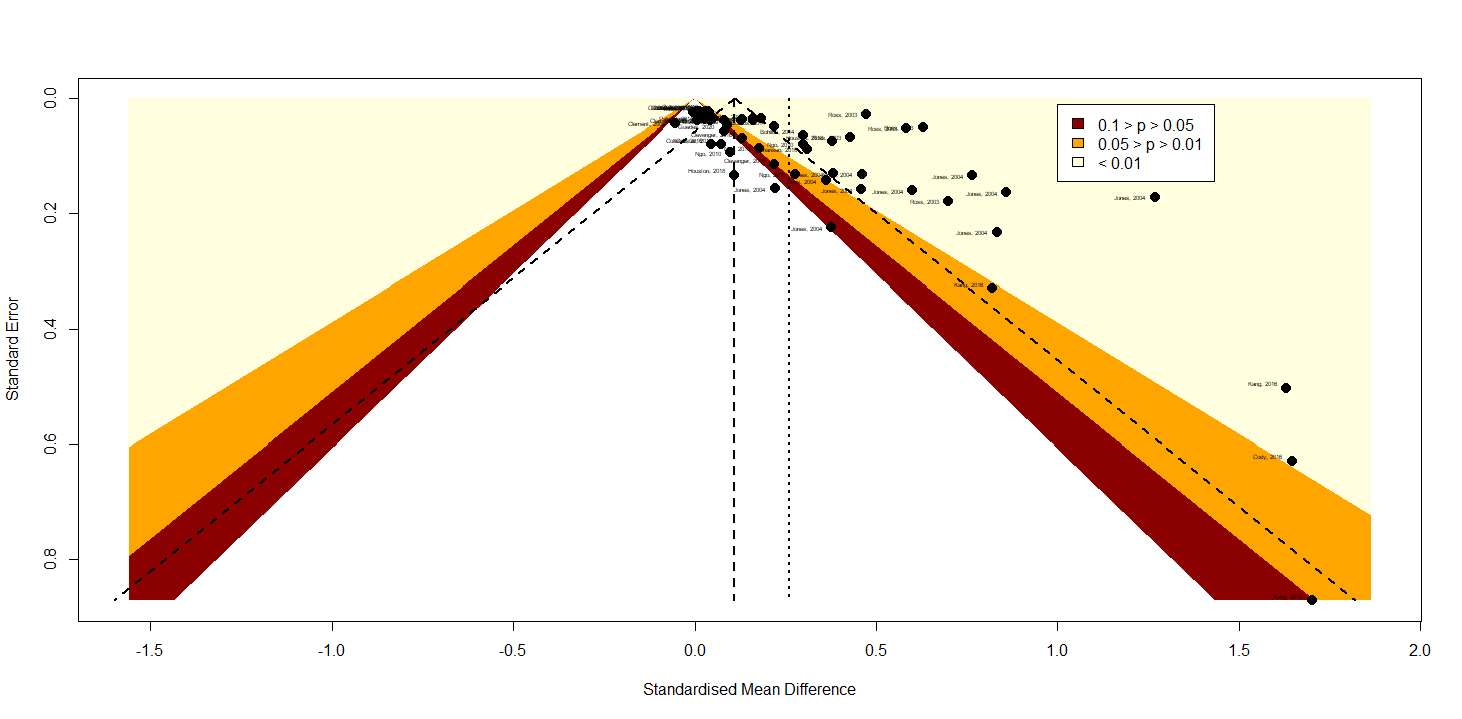
Supplementary material 4.** Funnel plots


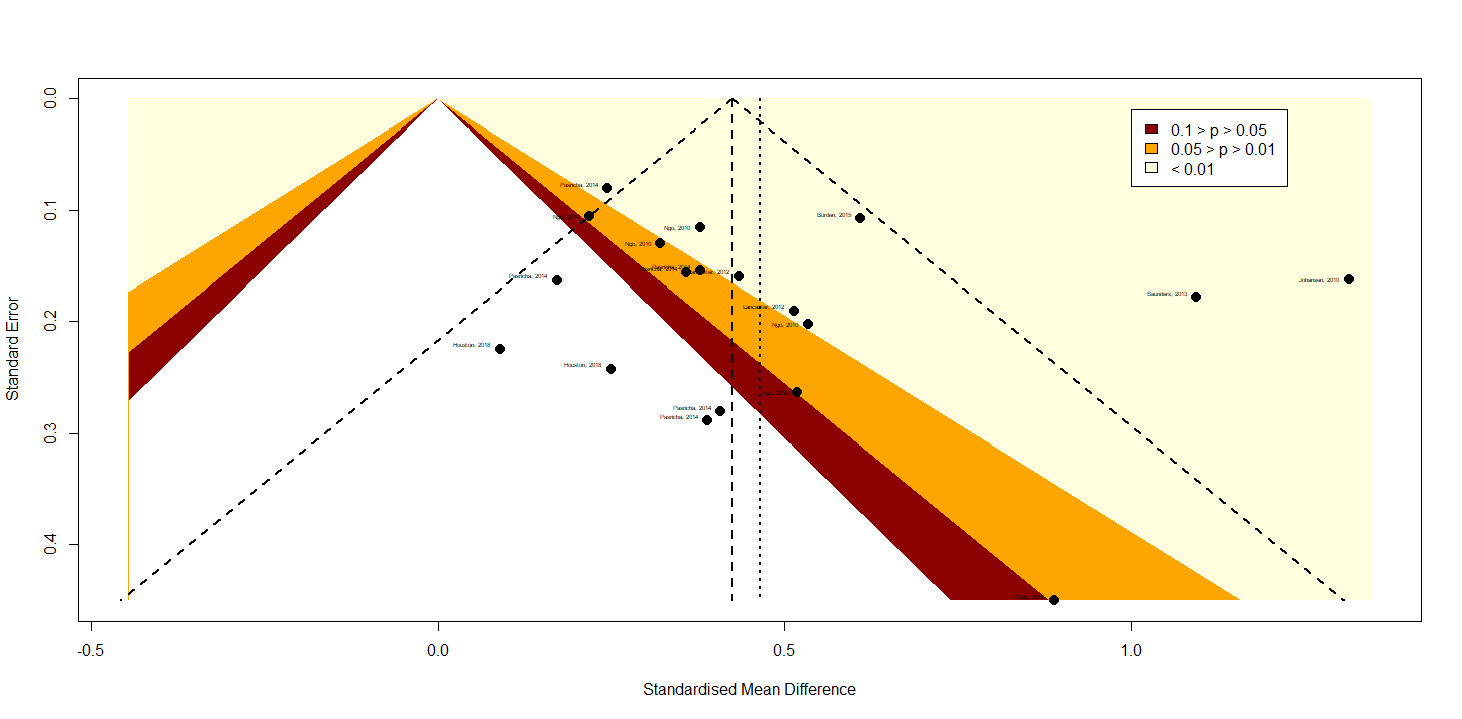


**Supplementary figure 1**. Funnel plot of systematic reviews assessing patient reported outcome measures. Egger’s test: P<0.001.

**Supplementary figure** **2**. Funnel plot of systematic reviews assessing physiological outcomes.
Egger’s test: P=0.277

#
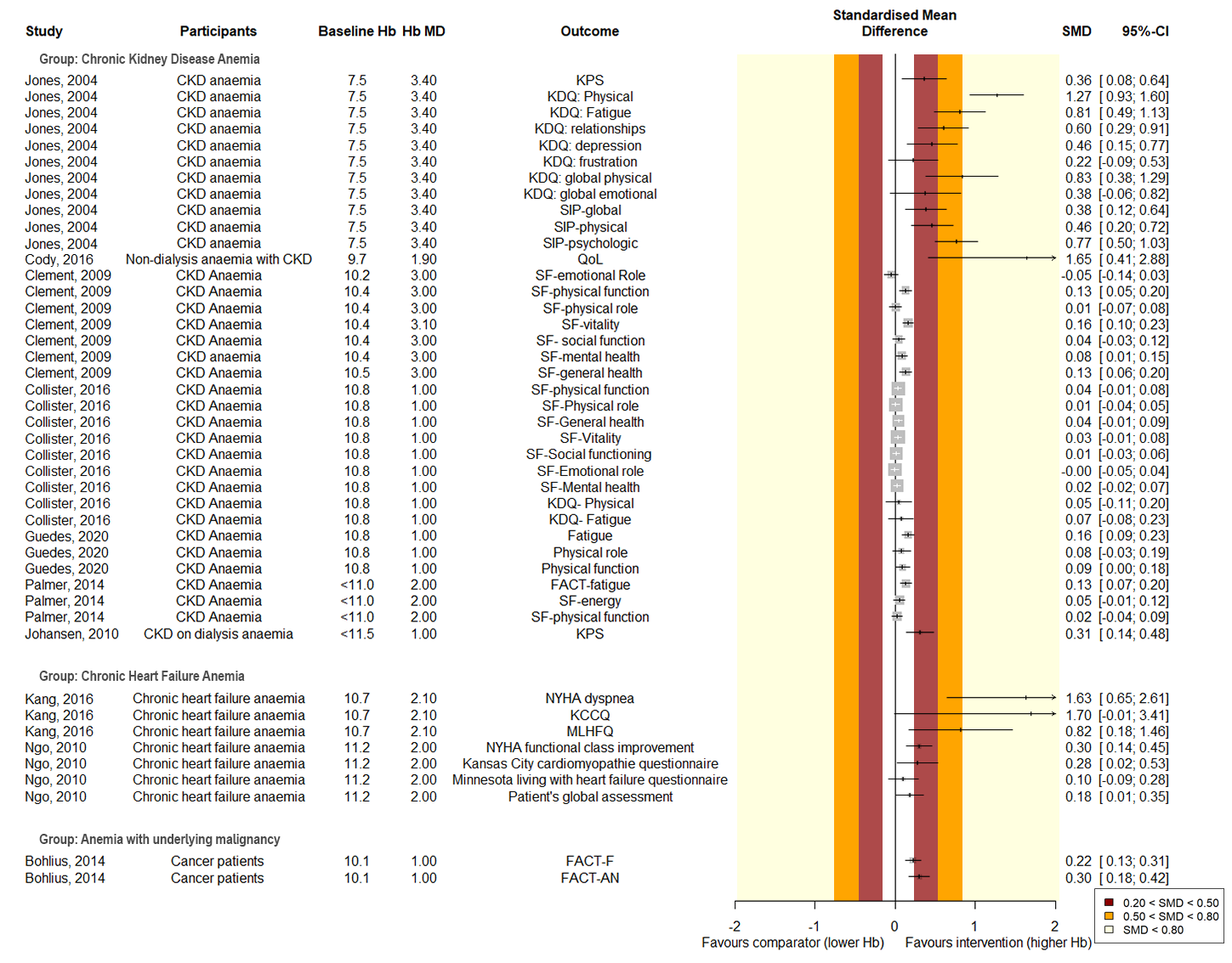
**Supplementary material 5**. ESA outcomes per patient group

**Supplementary figure** 3 and 4. Forest plots of systematic reviews assessing patient reported (3) and physiological (4) outcomes for ESA treatment, grouped per patient category, listed from lowest to highest baseline hemoglobin level. Standardized mean differences were computed by transforming the reported effect sizes using R “MBESS” or “esc” packages, depending on the available data. SMD values of 0.2-0.5 are considered small, 0.5-0.8 medium, and values > 0.8 are considered large. For overview purposes, if a Hb mean difference was not reported, we used the target instead in this figure. Also, not all baseline values were reported, in which case we used inclusion criteria cut-offs.


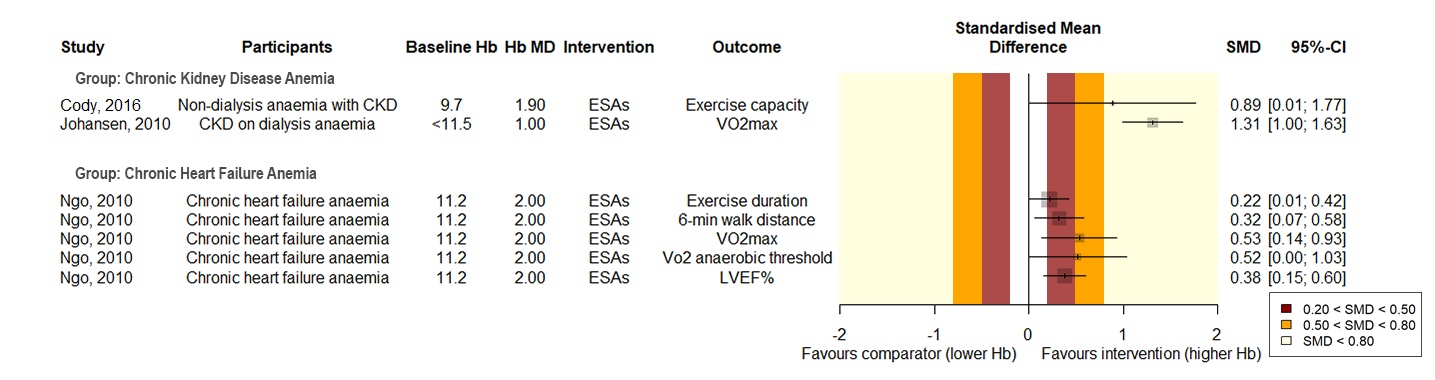


# **Supplementary material 6.** Sensitivity analysis figures

## **Sensitivity analysis 1:** Outcomes without reviews assessing ESAs


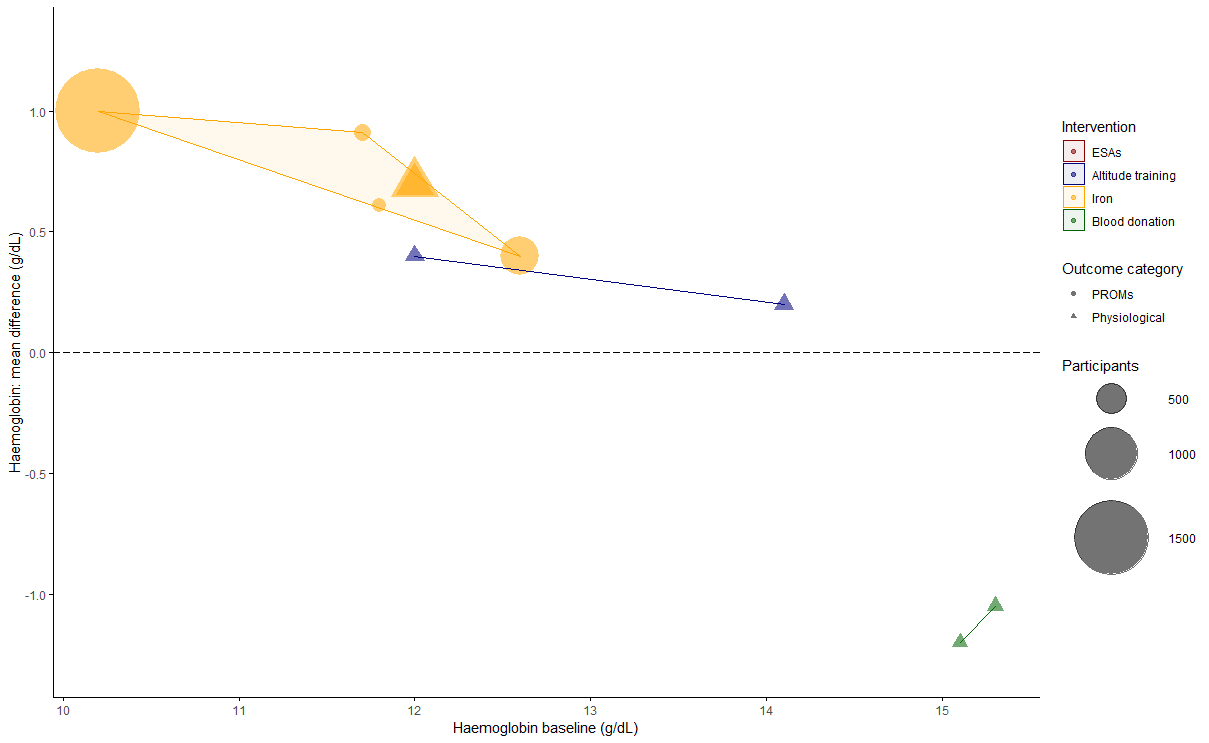


**Supplementary figure 5**. Bubble plot of included quantitative studies assessing Hb-change without ESAs. PROMs = patient reported outcome measures


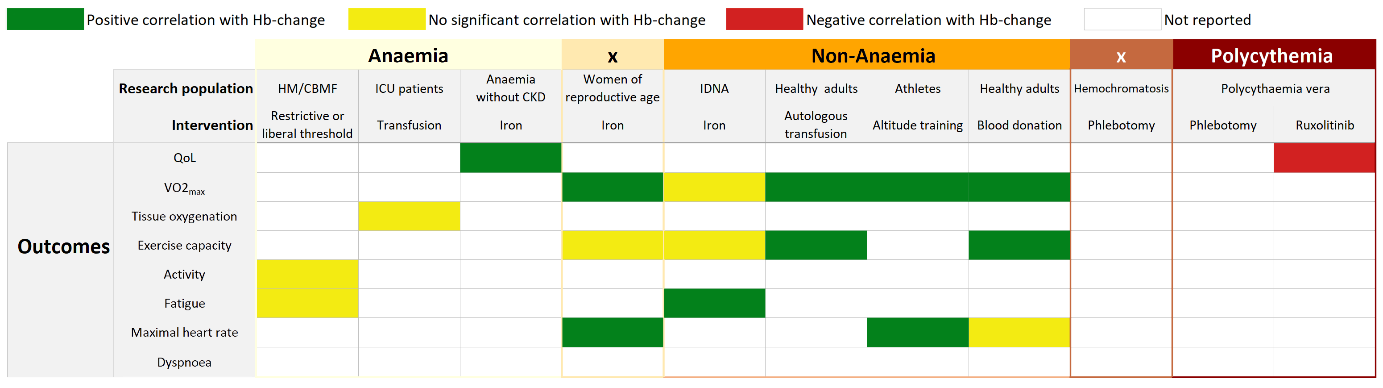


**Supplementary figure 6**. Overview of results of this umbrella review without ESAs. There is sufficient data for non-anaemic subjects. For anaemic subjects, however, very little systematic reviews remain.


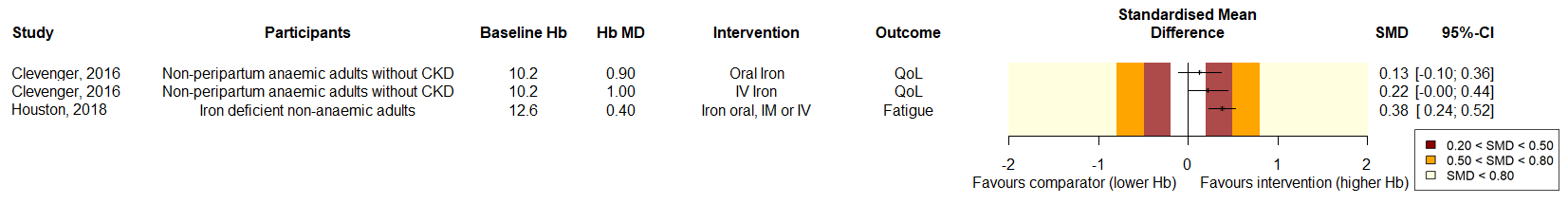


**Supplementary figure 7**. Forest plot of the effect of hemoglobin changes on patient reported outcome measures. Reviews assessing ESAs excluded as sensitivity analysis. Standardized mean differences were computed by transforming the in the primary reviews reported effect sizes using R “MBESS” or “esc” packages, depending on the available data. SMD values of 0.2-0.5 are considered small, 0.5-0.8 medium, and values > 0.8 are considered large. IM = intramuscular; IV = intravenous; TTE = Time to exhaustion;


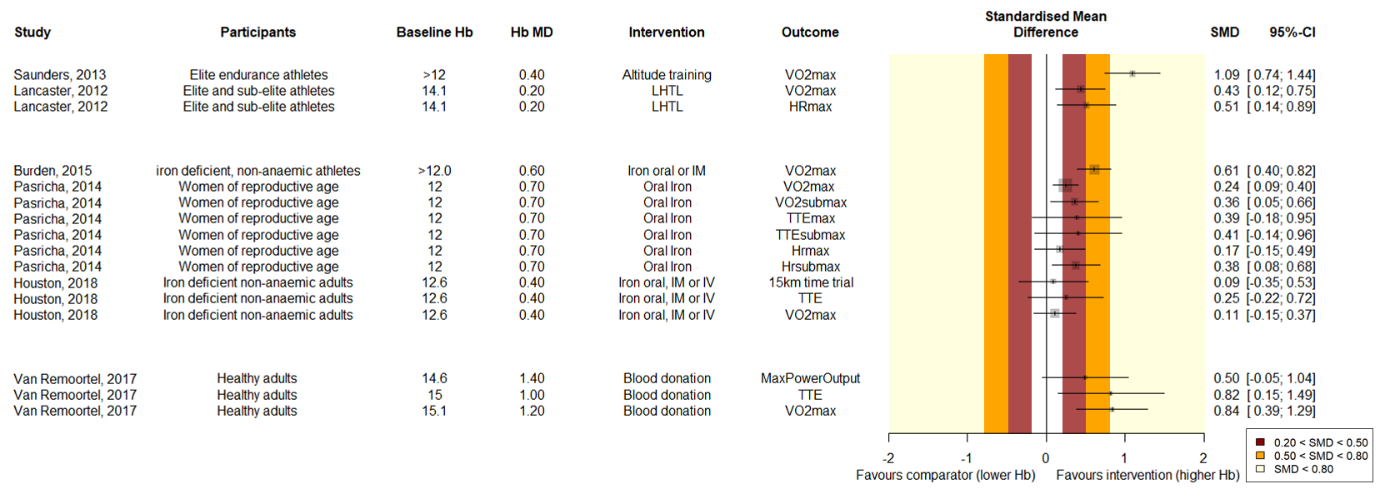


**Supplementary figure 8**. Forest plot of the effect of hemoglobin changes on physiological outcomes, transformed into standardized mean differences. Reviews assessing ESAs excluded as sensitivity analysis. SMD values of 0.2-0.5 are considered small, 0.5-0.8 medium, and values > 0.8 are considered large. LHTL = live high train low; IM = intramuscular; IV = intravenous; TTE = Time to exhaustion;

## **Sensitivity analysis 2**: Selection of high quality systematic reviews, as scored by the AMSTAR-2 tool.


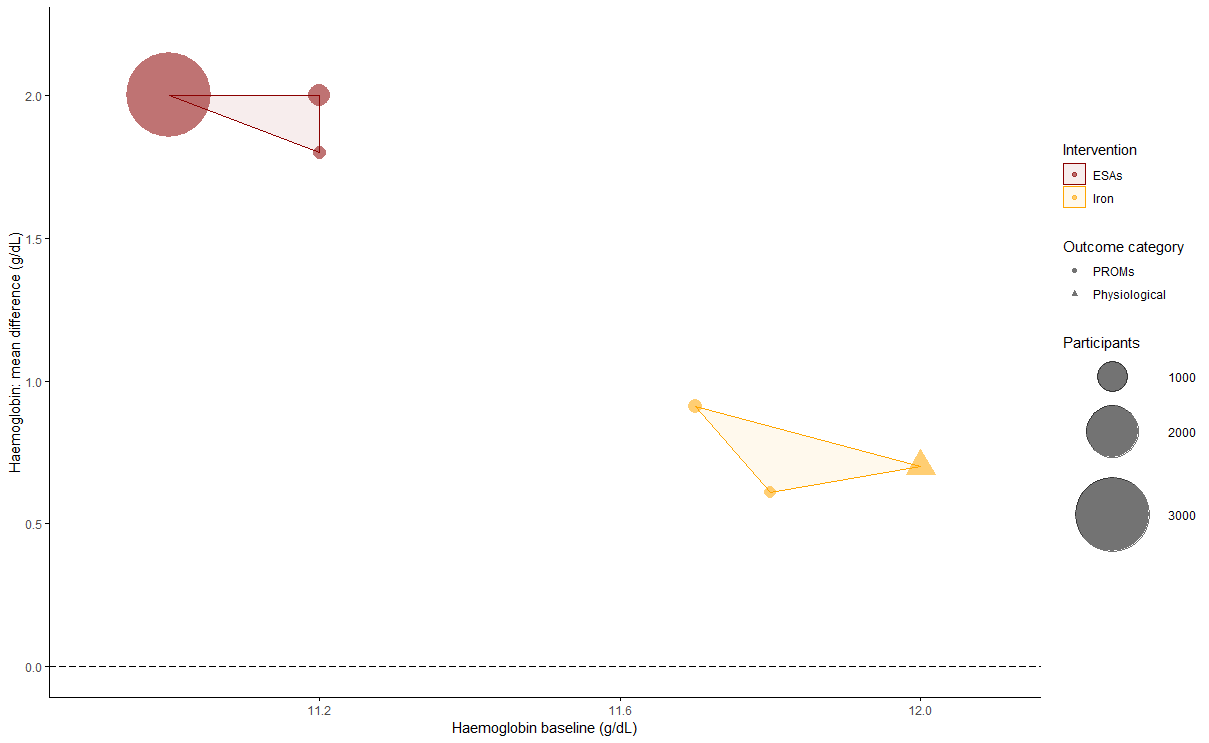


**Supplementary figure 9**. Bubble plot of included high quality systematic quantitative reviews assessing Hb-change. PROMs = patient reported outcome measures


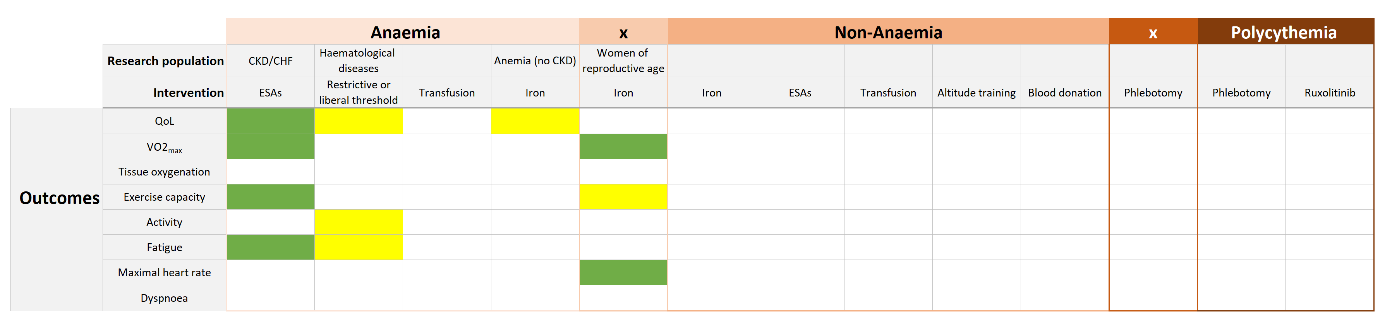


**Supplementary figure 10**. Overview of results of high quality systematic reviews of the present umbrella review. High quality evidence is only available for anaemic patient groups. No high quality reviews were included evaluating non-anaemic a polycythemic patient groups.


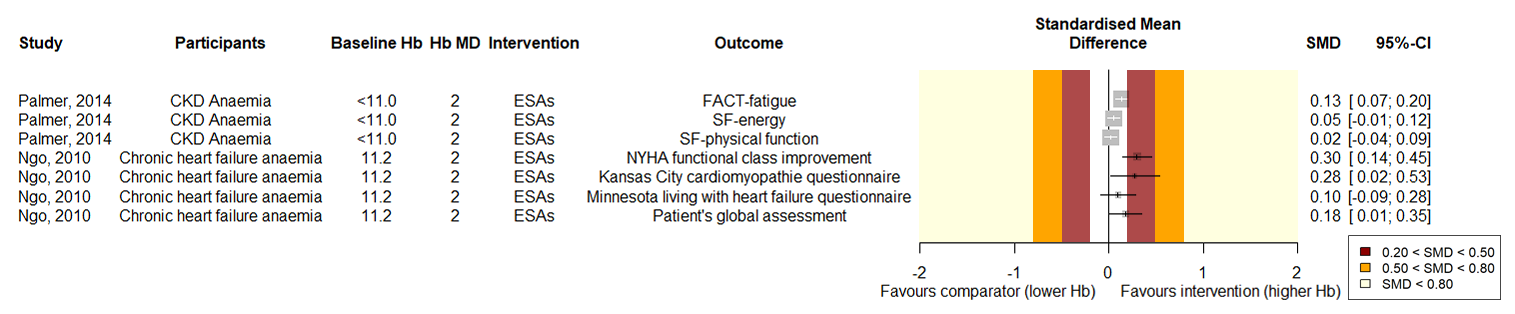


**Supplementary figure 11**. Forest plot of the effect of hemoglobin changes on patient reported outcome measures, transformed into standardized mean differences. A selection of high quality systematic reviews as sensitivity analysis. CKD = chronic kidney disease. Standardized mean differences were computed by transforming the in the primary reviews reported effect sizes using R “MBESS” or “esc” packages, depending on the available data. SMD values of 0.2-0.5 are considered small, 0.5-0.8 medium, and values > 0.8 are considered large.


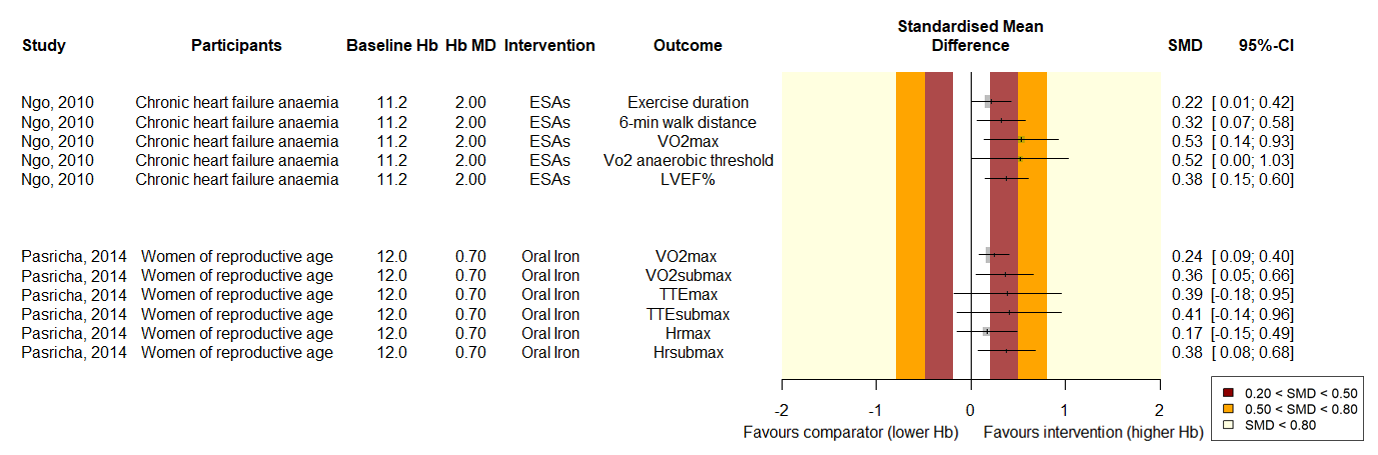


**Supplementary figure 12.** Forest plot of the effect of hemoglobin changes on physiological outcomes, transformed into standardized mean differences. A selection of high quality systematic reviews as sensitivity analysis. Standardized mean differences were computed by transforming the in the primary reviews reported effect sizes using R “MBESS” or “esc” packages, depending on the available data. SMD values of 0.2-0.5 are considered small, 0.5-0.8 medium, and values > 0.8 are considered large.
ESAs = Erythropoietin stimulating agents; LVEF% = % Left ventricle ejection fraction; TTE = Time to exhaustion; HR = heart rate.

# **Supplementary material 7. Extraction form**

**Modified JBI Extraction form**

| **Study details** |  |
| --- | --- |
| Author/year |  |
| Objectives |  |
| Type of participants |  |
| Control group |  |
| Number of participants |  |
| Setting/context |  |
| Description of Interventions |  |
| **Search details** |  |
| Sources searched |  |
| Range (years) included |  |
| Number of studies |  |
| Types of studies |  |
| Country of origin included studies |  |
| **Appraisal** |  |
| Appraisal instruments |  |
| Appraisal rating |  |
| **Analysis** |  |
| Method of analysis |  |
| Outcomes assessed |  |
| Results/findings |  |
| Significance/direction |  |
| Heterogeneity |  |
| **Comments** |  |
| Funding sources |  |

# **Supplementary material 8.** Search strategy

**Known refs:**

29113535 Sgro

24280034 Otto

24282203 Saunders

16516566 Calbet

20133033 Johansen

29141943 Stauder

Databases:

**PubMed**

<http://www.ncbi.nlm.nih.gov/pubmed?otool=leiden>

Basisvraag: 164 referenties, incl. Calbet, Johansen, Otto, Saunders, maar niet Sgro en Stauder

(("Hemoglobins"[Mesh] OR "Hemoglobins"[tw] OR "Haemoglobins"[tw] OR "Hemoglobin"[tw] OR "Haemoglobin"[tw] OR "Hemoglobin*"[tw] OR "Haemoglobin*"[tw] OR "alpha-Globin"[tw] OR "alpha-Globin*"[tw] OR "alpha-Globins"[tw] OR "beta-Globin"[tw] OR "beta-Globin*"[tw] OR "beta-Globins"[tw] OR "Carboxyhemoglobin"[tw] OR "Carboxyhemoglobin*"[tw] OR "Carboxyhemoglobins"[tw] OR "delta-Globin"[tw] OR "delta-Globin*"[tw] OR "delta-Globins"[tw] OR "epsilon-Globin"[tw] OR "epsilon-Globin*"[tw] OR "epsilon-Globins"[tw] OR "Erythrocruorin"[tw] OR "Erythrocruorin*"[tw] OR "Erythrocruorins"[tw] OR "gamma-Globin"[tw] OR "gamma-Globin*"[tw] OR "gamma-Globins"[tw] OR "Methemoglobin"[tw] OR "Methemoglobin*"[tw] OR "Methemoglobins"[tw] OR "Oxyhemoglobin"[tw] OR "Oxyhemoglobin*"[tw] OR "Oxyhemoglobins"[tw] OR "Sulfhemoglobin"[tw] OR "Sulfhemoglobin*"[tw] OR "Sulfhemoglobins"[tw] OR "zeta-Globin"[tw] OR "zeta-Globin*"[tw] OR "zeta-Globins"[tw]) **AND** ("mass"[ti] OR "concentration"[ti] OR "concentrations"[ti] OR "higher hemoglobin*"[tw] OR "higher haemoglobin*"[tw] OR "lower hemoglobin*"[tw] OR "lower haemoglobin*"[tw] OR "hemoglobin increas*"[tw] OR "haemoglobin increas*"[tw] OR "hemoglobin decreas*"[tw] OR "haemoglobin decreas*"[tw] OR "hemoglobin chang*"[tw] OR "haemoglobin chang*"[tw] OR "hemoglobin volume"[tw] OR "haemoglobin volume"[tw]) AND ("VO2max"[tw] OR "VO2 max"[tw] OR "VO2max*"[tw] OR "VO2 max*"[tw] OR "maximal oxygen uptake"[tw] OR "maximal oxygen consumption"[tw] OR "Oxygen Consumption"[Mesh] OR "Cardiac Output"[Mesh] OR "Cardiac output"[tw] OR "stroke volume"[tw] OR "Heart Rate"[Mesh] OR "Heart rate"[tw] OR "Heart Rates"[tw] OR "Cardiac Rate"[tw] OR "Cardiac Rates"[tw] OR "Pulse Rate"[tw] OR "Pulse Rates"[tw] OR "Heartbeat"[tw] OR "Heartbeats"[tw] OR "Cardiac Chronotropy"[tw] OR "Cardiac Chronotropism"[tw] OR "Respiratory Rate"[Mesh] OR "Respiratory rate"[tw] OR "Respiratory Rates"[tw] OR "Respiration Rate"[tw] OR "Respiration Rates"[tw] OR "physical capacity"[tw] OR "Physical Endurance"[Mesh] OR "Physical Endurance"[tw] OR "endurance"[tw] OR "stamina"[tw] OR "Anaerobic Threshold"[tw] OR "Exercise Tolerance"[tw] OR "Hand Strength"[Mesh] OR "Hand Strength"[tw] OR "grip strength"[tw] OR "pinch strength"[tw] OR "pinch strengths"[tw] OR "Hand Strengths"[tw] OR "Grip"[tw] OR "Grips"[tw] OR "Grasp"[tw] OR "Grasps"[tw] OR "muscle mass"[tw] OR "Muscle Strength"[Mesh] OR "Muscle Strength"[tw] OR "Cognition"[Mesh] OR "Cognition"[tw] OR "Cognit*"[tw] OR "Awareness"[tw] OR "Cognitive Dissonance"[tw] OR "Cognitive Reserve"[tw] OR "Comprehension"[tw] OR "Consciousness"[tw] OR "Imagination"[tw] OR "Dreams"[tw] OR "Fantasy"[tw] OR "Intuition"[tw] OR "Metacognition"[tw] OR "Cognitive Rumination"[tw] OR "cerebral function"[tw] OR "cerebral functions"[tw] OR "Mental Processes"[Mesh] OR "Fatigue"[Mesh] OR "Muscle Fatigue"[Mesh] OR "Fatigue"[tw] OR "Fatigue*"[tw] OR "Quality of Life"[mesh] OR "quality of life"[tw] OR "QoL"[tw] OR "life quality"[tw] OR "HRQoL"[tw] OR "PROM"[tw] OR "PROMs"[tw] OR "Patient Reported Outcome Measures"[Mesh] OR "Patient Reported Outcome"[tw] OR "Patient Reported Outcomes"[tw]) AND ("**review"[pt] OR "review"[tw] OR "overview"[tw] OR "review*"[tw] OR "overview*"[tw])** NOT ("acute anemia"[ti] OR "trauma"[ti] OR "trauma*"[ti] OR "Wounds and Injuries"[majr] OR "major surgery"[ti] OR "gastro-intestinal bleeding"[ti] OR "gastrointestinal bleeding"[ti] OR "Gastrointestinal Hemorrhage"[majr] OR "Gastrointestinal Hemorrhage"[ti] OR "Gastrointestinal Haemorrhage"[ti] OR "Gastro intestinal Hemorrhage"[ti] OR "Gastro intestinal Haemorrhage"[ti] OR "glycosylated"[ti] OR "Shock, Hemorrhagic"[majr] OR "hemorrhagic shock"[ti] OR "Transgender Persons"[majr] OR "transsexual*"[ti] OR "trans sexual*"[ti] OR "transgender*"[ti] OR "trans gender*"[ti] OR "acute bleeding"[ti] OR "acute hemorrhag*"[ti] OR "acute haemorrhag*"[ti]))

Sub-vraag 1: 598 referenties, incl. Johansen en Stauder, maar niet Calbet, Otto, Saunders, en Sgro.

(("chronic anaemia"[tw] OR "chronic anaemi*"[tw] OR "chronic anemia"[tw] OR "chronic anemi*"[tw] OR (("Anemia"[Mesh] OR "Anemia"[tw] OR "Anaemia"[tw] OR "Anemi*"[tw] OR "Anaemi*"[tw]) AND ("Chronic Disease"[Mesh] OR "Chronic"[tw] OR "Chronic*"[tw])) OR (("Anemia"[Mesh] OR "Anemia"[tw] OR "Anaemia"[tw] OR "Anemi*"[tw] OR "Anaemi*"[tw]) AND ("Renal Dialysis"[Mesh] OR "Renal Dialysis"[tw] OR "Hemodialysis"[tw] OR "Haemodialysis"[tw])) OR (("Anemia"[Mesh] OR "Anemia"[tw] OR "Anaemia"[tw] OR "Anemi*"[tw] OR "Anaemi*"[tw]) AND ("Myelodysplastic Syndromes"[Mesh] OR "Myelodysplastic Syndromes"[tw] OR "Myelodysplastic Syndromes"[tw] OR "MDS"[tw])) OR (("Anemia"[Mesh] OR "Anemia"[tw] OR "Anaemia"[tw] OR "Anemi*"[tw] OR "Anaemi*"[tw]) AND ("myeloproliferative neoplasms"[tw] OR "myeloproliferative neoplasms"[tw] OR "myeloproliferative neoplasia"[tw] OR "MPN"[tw] OR "MPNs"[tw])) OR "Anemia, Sickle Cell"[Mesh] OR "sickle cell anemia"[tw] OR "sickle cell anemi*"[tw] OR "sickle cell anaemia"[tw] OR "sickle cell anaemi*"[tw] OR "sickle cell disease"[tw] OR "Acute Chest Syndrome"[tw] OR "Haemoglobin SC Disease"[tw] OR "Hemoglobin SC Disease"[tw] OR "Sickle Cell Trait"[tw] OR "Thalassemia"[Mesh] OR "Thalassemia"[tw] OR "Thalassemi*"[tw] OR "Anemia, Aplastic"[Mesh] OR "aplastic anemia"[tw] OR "aplastic anemi*"[tw] OR "aplastic anaemia"[tw] OR "aplastic anaemi*"[tw] OR "fanconi anemia"[tw] OR "fanconi anemi*"[tw] OR "fanconi anaemia"[tw] OR "fanconi anaemi*"[tw] OR "diamond-blackfan anemia"[tw] OR "diamond-blackfan anemi*"[tw] OR "diamond-blackfan anaemia"[tw] OR "diamond-blackfan anaemi*"[tw] OR "congenital hypoplastic anemia"[tw] OR "congenital hypoplastic anemi*"[tw] OR "congenital hypoplastic anaemia"[tw] OR "congenital hypoplastic anaemi*"[tw]) AND ("Erythropoietin"[Mesh] OR "Erythropoietin"[tw] OR "Erythropoietin*"[tw] OR "EPO"[tw] OR "epoetin"[tw] OR "liberal transfusion strategy"[tw] OR "liberal transfusion"[tw] OR "higher nadir"[tw] OR "higher nadir*"[tw] OR "higher threshold"[tw] OR "higher threshold*"[tw] OR "iron therapy"[tw] OR "iron therap*"[tw] OR "iron treatment"[tw] OR "iron treat*"[tw] OR "Iron/therapeutic use"[mesh] OR "altitude training"[tw] OR (("Altitude"[mesh] OR "altitude"[tw] OR "altitude*"[tw]) AND ("training"[tw] OR "Exercise"[mesh] OR "exercise"[tw]))) AND ("VO2max"[tw] OR "VO2 max"[tw] OR "VO2max*"[tw] OR "VO2 max*"[tw] OR "maximal oxygen uptake"[tw] OR "maximal oxygen consumption"[tw] OR "Oxygen Consumption"[Mesh] OR "Cardiac Output"[Mesh] OR "Cardiac output"[tw] OR "stroke volume"[tw] OR "Heart Rate"[Mesh] OR "Heart rate"[tw] OR "Heart Rates"[tw] OR "Cardiac Rate"[tw] OR "Cardiac Rates"[tw] OR "Pulse Rate"[tw] OR "Pulse Rates"[tw] OR "Heartbeat"[tw] OR "Heartbeats"[tw] OR "Cardiac Chronotropy"[tw] OR "Cardiac Chronotropism"[tw] OR "Respiratory Rate"[Mesh] OR "Respiratory rate"[tw] OR "Respiratory Rates"[tw] OR "Respiration Rate"[tw] OR "Respiration Rates"[tw] OR "physical capacity"[tw] OR "Physical Endurance"[Mesh] OR "Physical Endurance"[tw] OR "endurance"[tw] OR "stamina"[tw] OR "Anaerobic Threshold"[tw] OR "Exercise Tolerance"[tw] OR "Hand Strength"[Mesh] OR "Hand Strength"[tw] OR "grip strength"[tw] OR "pinch strength"[tw] OR "pinch strengths"[tw] OR "Hand Strengths"[tw] OR "Grip"[tw] OR "Grips"[tw] OR "Grasp"[tw] OR "Grasps"[tw] OR "muscle mass"[tw] OR "Muscle Strength"[Mesh] OR "Muscle Strength"[tw] OR "Cognition"[Mesh] OR "Cognition"[tw] OR "Cognit*"[tw] OR "Awareness"[tw] OR "Cognitive Dissonance"[tw] OR "Cognitive Reserve"[tw] OR "Comprehension"[tw] OR "Consciousness"[tw] OR "Imagination"[tw] OR "Dreams"[tw] OR "Fantasy"[tw] OR "Intuition"[tw] OR "Metacognition"[tw] OR "Cognitive Rumination"[tw] OR "cerebral function"[tw] OR "cerebral functions"[tw] OR "Mental Processes"[Mesh] OR "Fatigue"[Mesh] OR "Muscle Fatigue"[Mesh] OR "Fatigue"[tw] OR "Fatigue*"[tw] OR "Quality of Life"[mesh] OR "quality of life"[tw] OR "QoL"[tw] OR "life quality"[tw] OR "HRQoL"[tw] OR "PROM"[tw] OR "PROMs"[tw] OR "Patient Reported Outcome Measures"[Mesh] OR "Patient Reported Outcome"[tw] OR "Patient Reported Outcomes"[tw]) AND ("**review"[pt] OR "review"[tw] OR "overview"[tw] OR "review*"[tw] OR "overview*"[tw])** NOT ("acute anemia"[ti] OR "trauma"[ti] OR "trauma*"[ti] OR "Wounds and Injuries"[majr] OR "major surgery"[ti] OR "gastro-intestinal bleeding"[ti] OR "gastrointestinal bleeding"[ti] OR "Gastrointestinal Hemorrhage"[majr] OR "Gastrointestinal Hemorrhage"[ti] OR "Gastrointestinal Haemorrhage"[ti] OR "Gastro intestinal Hemorrhage"[ti] OR "Gastro intestinal Haemorrhage"[ti] OR ''glycosylated''[ti] OR "glycosylated"[ti] OR "Shock, Hemorrhagic"[majr] OR "hemorrhagic shock"[ti] OR "Transgender Persons"[majr] OR "transsexual*"[ti] OR "trans sexual*"[ti] OR "transgender*"[ti] OR "trans gender*"[ti] OR "acute bleeding"[ti] OR "acute hemorrhag*"[ti] OR "acute haemorrhag*"[ti]))

Sub-vraag 2: 179 referenties, incl. Saunders en Sgro, maar niet, Calbet, Johansen, Otto en Stauder.

(("Healthy Volunteers"[Mesh] OR "Healthy Participant"[tw] OR "Healthy Participants"[tw] OR "Healthy Subject"[tw] OR "Healthy Subjects"[tw] OR "Healthy Volunteer"[tw] OR "Human Volunteer"[tw] OR "Human Volunteers"[tw] OR "Normal Volunteer"[tw] OR "Normal Volunteers"[tw] OR "Athletes"[Mesh] OR "Athletes"[tw] OR "Athlete"[tw] OR "fit patients"[tw] OR "fit patient"[tw]) AND ("Erythropoietin"[Mesh] OR "Erythropoietin"[tw] OR "Erythropoietin*"[tw] OR "EPO"[tw] OR "epoetin"[tw] OR "liberal transfusion strategy"[tw] OR "liberal transfusion"[tw] OR "higher nadir"[tw] OR "higher nadir*"[tw] OR "higher threshold"[tw] OR "higher threshold*"[tw] OR "iron therapy"[tw] OR "iron therap*"[tw] OR "iron treatment"[tw] OR "iron treat*"[tw] OR "Iron/therapeutic use"[mesh] OR "altitude training"[tw] OR (("Altitude"[mesh] OR "altitude"[tw] OR "altitude*"[tw]) AND ("training"[tw] OR "Exercise"[mesh] OR "exercise"[tw]))) AND ("VO2max"[tw] OR "VO2 max"[tw] OR "VO2max*"[tw] OR "VO2 max*"[tw] OR "maximal oxygen uptake"[tw] OR "maximal oxygen consumption"[tw] OR "Oxygen Consumption"[Mesh] OR "Cardiac Output"[Mesh] OR "Cardiac output"[tw] OR "stroke volume"[tw] OR "Heart Rate"[Mesh] OR "Heart rate"[tw] OR "Heart Rates"[tw] OR "Cardiac Rate"[tw] OR "Cardiac Rates"[tw] OR "Pulse Rate"[tw] OR "Pulse Rates"[tw] OR "Heartbeat"[tw] OR "Heartbeats"[tw] OR "Cardiac Chronotropy"[tw] OR "Cardiac Chronotropism"[tw] OR "Respiratory Rate"[Mesh] OR "Respiratory rate"[tw] OR "Respiratory Rates"[tw] OR "Respiration Rate"[tw] OR "Respiration Rates"[tw] OR "physical capacity"[tw] OR "Physical Endurance"[Mesh] OR "Physical Endurance"[tw] OR "endurance"[tw] OR "stamina"[tw] OR "Anaerobic Threshold"[tw] OR "Exercise Tolerance"[tw] OR "Hand Strength"[Mesh] OR "Hand Strength"[tw] OR "grip strength"[tw] OR "pinch strength"[tw] OR "pinch strengths"[tw] OR "Hand Strengths"[tw] OR "Grip"[tw] OR "Grips"[tw] OR "Grasp"[tw] OR "Grasps"[tw] OR "muscle mass"[tw] OR "Muscle Strength"[Mesh] OR "Muscle Strength"[tw] OR "Cognition"[Mesh] OR "Cognition"[tw] OR "Cognit*"[tw] OR "Awareness"[tw] OR "Cognitive Dissonance"[tw] OR "Cognitive Reserve"[tw] OR "Comprehension"[tw] OR "Consciousness"[tw] OR "Imagination"[tw] OR "Dreams"[tw] OR "Fantasy"[tw] OR "Intuition"[tw] OR "Metacognition"[tw] OR "Cognitive Rumination"[tw] OR "cerebral function"[tw] OR "cerebral functions"[tw] OR "Mental Processes"[Mesh] OR "Fatigue"[Mesh] OR "Muscle Fatigue"[Mesh] OR "Fatigue"[tw] OR "Fatigue*"[tw] OR "Quality of Life"[mesh] OR "quality of life"[tw] OR "QoL"[tw] OR "life quality"[tw] OR "HRQoL"[tw] OR "PROM"[tw] OR "PROMs"[tw] OR "Patient Reported Outcome Measures"[Mesh] OR "Patient Reported Outcome"[tw] OR "Patient Reported Outcomes"[tw]) AND ("**review"[pt] OR "review"[tw] OR "overview"[tw] OR "review*"[tw] OR "overview*"[tw])** NOT ("acute anemia"[ti] OR "trauma"[ti] OR "trauma*"[ti] OR "Wounds and Injuries"[majr] OR "major surgery"[ti] OR "gastro-intestinal bleeding"[ti] OR "gastrointestinal bleeding"[ti] OR "Gastrointestinal Hemorrhage"[majr] OR "Gastrointestinal Hemorrhage"[ti] OR "Gastrointestinal Haemorrhage"[ti] OR "Gastro intestinal Hemorrhage"[ti] OR "Gastro intestinal Haemorrhage"[ti] OR ''glycosylated''[ti] OR "glycosylated"[ti] OR "Shock, Hemorrhagic"[majr] OR "hemorrhagic shock"[ti] OR "Transgender Persons"[majr] OR "transsexual*"[ti] OR "trans sexual*"[ti] OR "transgender*"[ti] OR "trans gender*"[ti] OR "acute bleeding"[ti] OR "acute hemorrhag*"[ti] OR "acute haemorrhag*"[ti]))

Sub-vraag 3: 44 referenties, excl. de zes bekende referenties.

(("Erythrocythemia"[tw] OR "Erythrocythem*"[tw] OR "Erythrocythaemia"[tw] OR "Erythrocythaem*"[tw] OR "Erythrocytemia"[tw] OR "Erythrocytem*"[tw] OR "Erythrocytaemia"[tw] OR "Erythrocytaem*"[tw] OR "Polycythemia Vera"[Mesh] OR "Polycythemia"[Mesh] OR "Polycythemia"[tw] OR "Polycythemi*"[tw] OR "Polycythaemia"[tw] OR "Polycythaemi*"[tw] OR "erythrocytosis"[tw] OR "erytrocytosis"[tw]) AND ("Phlebotomy"[Mesh] OR "Phlebotomy"[tw] OR "Phlebotom*"[tw] OR "Venesection*"[tw] OR "Venipuncture*"[tw] OR "Hydroxyurea"[Mesh] OR "Hydroxyurea"[tw] OR "Hydroxyurea*"[tw] OR "hydrea"[tw] OR "Hydroxycarbamid*"[tw] OR "Oncocarbide*"[tw] OR "Busulfan"[Mesh] OR "Busulfan"[tw] OR "Busulfan*"[tw] OR "Busulphan"[tw] OR "Busulfex"[tw] OR "Myleran"[tw] OR "Myelosan"[tw] OR "Mylecytan"[tw] OR "Glyzophrol"[tw] OR "Interferons"[Mesh] OR "Interferons"[tw] OR "Interferon"[tw] OR "Interferon*"[tw]) AND ("VO2max"[tw] OR "VO2 max"[tw] OR "VO2max*"[tw] OR "VO2 max*"[tw] OR "maximal oxygen uptake"[tw] OR "maximal oxygen consumption"[tw] OR "Oxygen Consumption"[Mesh] OR "Cardiac Output"[Mesh] OR "Cardiac output"[tw] OR "stroke volume"[tw] OR "Heart Rate"[Mesh] OR "Heart rate"[tw] OR "Heart Rates"[tw] OR "Cardiac Rate"[tw] OR "Cardiac Rates"[tw] OR "Pulse Rate"[tw] OR "Pulse Rates"[tw] OR "Heartbeat"[tw] OR "Heartbeats"[tw] OR "Cardiac Chronotropy"[tw] OR "Cardiac Chronotropism"[tw] OR "Respiratory Rate"[Mesh] OR "Respiratory rate"[tw] OR "Respiratory Rates"[tw] OR "Respiration Rate"[tw] OR "Respiration Rates"[tw] OR "physical capacity"[tw] OR "Physical Endurance"[Mesh] OR "Physical Endurance"[tw] OR "endurance"[tw] OR "stamina"[tw] OR "Anaerobic Threshold"[tw] OR "Exercise Tolerance"[tw] OR "Hand Strength"[Mesh] OR "Hand Strength"[tw] OR "grip strength"[tw] OR "pinch strength"[tw] OR "pinch strengths"[tw] OR "Hand Strengths"[tw] OR "Grip"[tw] OR "Grips"[tw] OR "Grasp"[tw] OR "Grasps"[tw] OR "muscle mass"[tw] OR "Muscle Strength"[Mesh] OR "Muscle Strength"[tw] OR "Cognition"[Mesh] OR "Cognition"[tw] OR "Cognit*"[tw] OR "Awareness"[tw] OR "Cognitive Dissonance"[tw] OR "Cognitive Reserve"[tw] OR "Comprehension"[tw] OR "Consciousness"[tw] OR "Imagination"[tw] OR "Dreams"[tw] OR "Fantasy"[tw] OR "Intuition"[tw] OR "Metacognition"[tw] OR "Cognitive Rumination"[tw] OR "cerebral function"[tw] OR "cerebral functions"[tw] OR "Mental Processes"[Mesh] OR "Fatigue"[Mesh] OR "Muscle Fatigue"[Mesh] OR "Fatigue"[tw] OR "Fatigue*"[tw] OR "Quality of Life"[mesh] OR "quality of life"[tw] OR "QoL"[tw] OR "life quality"[tw] OR "HRQoL"[tw] OR "PROM"[tw] OR "PROMs"[tw] OR "Patient Reported Outcome Measures"[Mesh] OR "Patient Reported Outcome"[tw] OR "Patient Reported Outcomes"[tw]) AND ("**review"[pt] OR "review"[tw] OR "overview"[tw] OR "review*"[tw] OR "overview*"[tw])** NOT ("acute anemia"[ti] OR "trauma"[ti] OR "trauma*"[ti] OR "Wounds and Injuries"[majr] OR "major surgery"[ti] OR "gastro-intestinal bleeding"[ti] OR "gastrointestinal bleeding"[ti] OR "Gastrointestinal Hemorrhage"[majr] OR "Gastrointestinal Hemorrhage"[ti] OR "Gastrointestinal Haemorrhage"[ti] OR "Gastro intestinal Hemorrhage"[ti] OR "Gastro intestinal Haemorrhage"[ti] OR ''glycosylated''[ti] OR "glycosylated"[ti] OR "Shock, Hemorrhagic"[majr] OR "hemorrhagic shock"[ti] OR "Transgender Persons"[majr] OR "transsexual*"[ti] OR "trans sexual*"[ti] OR "transgender*"[ti] OR "trans gender*"[ti] OR "acute bleeding"[ti] OR "acute hemorrhag*"[ti] OR "acute haemorrhag*"[ti]))

**MEDLINE via OVID**

<http://gateway.ovid.com/ovidweb.cgi?T=JS&MODE=ovid&NEWS=n&PAGE=main&D=medall>

(29113535 OR 24280034 OR 24282203 OR 16516566 OR 20133033 OR 29141943).ui

Basisvraag: 438 referenties

- met nabijheidsoperator Adjacency - vangst neemt met ca. 50 af, zonder veel aan relevantie in te boeten (van 843 naar 438)

(zonder de mesh exp "Hemoglobins"/)

(("Hemoglobins".mp OR "Haemoglobins".mp OR "Hemoglobin".mp OR "Haemoglobin".mp OR "Hemoglobin*".mp OR "Haemoglobin*".mp OR "alpha-Globin".mp OR "alpha-Globin*".mp OR "alpha-Globins".mp OR "beta-Globin".mp OR "beta-Globin*".mp OR "beta-Globins".mp OR "Carboxyhemoglobin".mp OR "Carboxyhemoglobin*".mp OR "Carboxyhemoglobins".mp OR "delta-Globin".mp OR "delta-Globin*".mp OR "delta-Globins".mp OR "epsilon-Globin".mp OR "epsilon-Globin*".mp OR "epsilon-Globins".mp OR "Erythrocruorin".mp OR "Erythrocruorin*".mp OR "Erythrocruorins".mp OR "gamma-Globin".mp OR "gamma-Globin*".mp OR "gamma-Globins".mp OR "Methemoglobin".mp OR "Methemoglobin*".mp OR "Methemoglobins".mp OR "Oxyhemoglobin".mp OR "Oxyhemoglobin*".mp OR "Oxyhemoglobins".mp OR "Sulfhemoglobin".mp OR "Sulfhemoglobin*".mp OR "Sulfhemoglobins".mp OR "zeta-Globin".mp OR "zeta-Globin*".mp OR "zeta-Globins".mp) **ADJ4** ("mass".mp OR "concentration".mp OR "concentrations".mp OR "higher hemoglobin*".mp OR "higher haemoglobin*".mp OR "lower hemoglobin*".mp OR "lower haemoglobin*".mp OR "hemoglobin increas*".mp OR "haemoglobin increas*".mp OR "hemoglobin decreas*".mp OR "haemoglobin decreas*".mp OR "hemoglobin chang*".mp OR "haemoglobin chang*".mp OR "hemoglobin volume".mp OR "haemoglobin volume".mp) AND ("VO2max".mp OR "VO2 max".mp OR "VO2max*".mp OR "VO2 max*".mp OR "maximal oxygen uptake".mp OR "maximal oxygen consumption".mp OR exp "Oxygen Consumption"/ OR exp "Cardiac Output"/ OR "Cardiac output".mp OR "stroke volume".mp OR exp "Heart Rate"/ OR "Heart rate".mp OR "Heart Rates".mp OR "Cardiac Rate".mp OR "Cardiac Rates".mp OR "Pulse Rate".mp OR "Pulse Rates".mp OR "Heartbeat".mp OR "Heartbeats".mp OR "Cardiac Chronotropy".mp OR "Cardiac Chronotropism".mp OR exp "Respiratory Rate"/ OR "Respiratory rate".mp OR "Respiratory Rates".mp OR "Respiration Rate".mp OR "Respiration Rates".mp OR "physical capacity".mp OR exp "Physical Endurance"/ OR "Physical Endurance".mp OR "endurance".mp OR "stamina".mp OR "Anaerobic Threshold".mp OR "Exercise Tolerance".mp OR exp "Hand Strength"/ OR "Hand Strength".mp OR "grip strength".mp OR "pinch strength".mp OR "pinch strengths".mp OR "Hand Strengths".mp OR "Grip".mp OR "Grips".mp OR "Grasp".mp OR "Grasps".mp OR "muscle mass".mp OR exp "Muscle Strength"/ OR "Muscle Strength".mp OR exp "Cognition"/ OR "Cognition".mp OR "Cognit*".mp OR "Awareness".mp OR "Cognitive Dissonance".mp OR "Cognitive Reserve".mp OR "Comprehension".mp OR "Consciousness".mp OR "Imagination".mp OR "Dreams".mp OR "Fantasy".mp OR "Intuition".mp OR "Metacognition".mp OR "Cognitive Rumination".mp OR "cerebral function".mp OR "cerebral functions".mp OR exp "Mental Processes"/ OR exp "Fatigue"/ OR exp "Muscle Fatigue"/ OR "Fatigue".mp OR "Fatigue*".mp OR exp "Quality of Life"/ OR "quality of life".mp OR "QoL".mp OR "life quality".mp OR "HRQoL".mp OR "PROM".mp OR "PROMs".mp OR exp "Patient Reported Outcome Measures"/ OR "Patient Reported Outcome".mp OR "Patient Reported Outcomes".mp) AND **(exp** "**review"/ OR "review".mp OR "overview".mp OR "review*".mp OR "overview*".mp)** NOT ("acute anemia".ti OR "trauma".ti OR "trauma*".ti OR exp *"Wounds and Injuries"/ OR "major surgery".ti OR "gastro-intestinal bleeding".ti OR "gastrointestinal bleeding".ti OR exp *"Gastrointestinal Hemorrhage"/ OR "Gastrointestinal Hemorrhage".ti OR "Gastrointestinal Haemorrhage".ti OR "Gastro intestinal Hemorrhage".ti OR "Gastro intestinal Haemorrhage".ti OR "glycosylated".ti OR exp *"Shock, Hemorrhagic"/ OR "hemorrhagic shock".ti OR exp *"Transgender Persons"/ OR "transsexual*".ti OR "trans sexual*".ti OR "transgender*".ti OR "trans gender*".ti OR "acute bleeding".ti OR "acute hemorrhag*".ti OR "acute haemorrhag*".ti))

**Embase**

<http://ovidsp.ovid.com/ovidweb.cgi?T=JS&PAGE=main&MODE=ovid&D=oemezd>

Basisvraag:

- met nabijheidsoperator Adjacency (zonder de mesh exp "Hemoglobin"/)

(("Hemoglobins".ti,ab OR "Haemoglobins".ti,ab OR "Hemoglobin".ti,ab OR "Haemoglobin".ti,ab OR "Hemoglobin*".ti,ab OR "Haemoglobin*".ti,ab OR "alpha-Globin".ti,ab OR "alpha-Globin*".ti,ab OR "alpha-Globins".ti,ab OR "beta-Globin".ti,ab OR "beta-Globin*".ti,ab OR "beta-Globins".ti,ab OR "Carboxyhemoglobin".ti,ab OR "Carboxyhemoglobin*".ti,ab OR "Carboxyhemoglobins".ti,ab OR "delta-Globin".ti,ab OR "delta-Globin*".ti,ab OR "delta-Globins".ti,ab OR "epsilon-Globin".ti,ab OR "epsilon-Globin*".ti,ab OR "epsilon-Globins".ti,ab OR "Erythrocruorin".ti,ab OR "Erythrocruorin*".ti,ab OR "Erythrocruorins".ti,ab OR "gamma-Globin".ti,ab OR "gamma-Globin*".ti,ab OR "gamma-Globins".ti,ab OR "Methemoglobin".ti,ab OR "Methemoglobin*".ti,ab OR "Methemoglobins".ti,ab OR "Oxyhemoglobin".ti,ab OR "Oxyhemoglobin*".ti,ab OR "Oxyhemoglobins".ti,ab OR "Sulfhemoglobin".ti,ab OR "Sulfhemoglobin*".ti,ab OR "Sulfhemoglobins".ti,ab OR "zeta-Globin".ti,ab OR "zeta-Globin*".ti,ab OR "zeta-Globins".ti,ab) **ADJ4** ("mass".ti,ab OR "concentration".ti,ab OR "concentrations".ti,ab OR "higher hemoglobin*".ti,ab OR "higher haemoglobin*".ti,ab OR "lower hemoglobin*".ti,ab OR "lower haemoglobin*".ti,ab OR "hemoglobin increas*".ti,ab OR "haemoglobin increas*".ti,ab OR "hemoglobin decreas*".ti,ab OR "haemoglobin decreas*".ti,ab OR "hemoglobin chang*".ti,ab OR "haemoglobin chang*".ti,ab OR "hemoglobin volume".ti,ab OR "haemoglobin volume".ti,ab) AND ("VO2max".ti,ab OR "VO2 max".ti,ab OR "VO2max*".ti,ab OR "VO2 max*".ti,ab OR "maximal oxygen uptake".ti,ab OR "maximal oxygen consumption".ti,ab OR exp *"Maximal Oxygen Uptake"/ OR exp *"Heart Output"/ OR "Cardiac output".ti,ab OR "stroke volume".ti,ab OR exp *"Heart Rate"/ OR "Heart rate".ti,ab OR "Heart Rates".ti,ab OR "Cardiac Rate".ti,ab OR "Cardiac Rates".ti,ab OR "Pulse Rate".ti,ab OR "Pulse Rates".ti,ab OR "Heartbeat".ti,ab OR "Heartbeats".ti,ab OR "Cardiac Chronotropy".ti,ab OR "Cardiac Chronotropism".ti,ab OR exp *"Breathing Rate"/ OR "Respiratory rate".ti,ab OR "Respiratory Rates".ti,ab OR "Respiration Rate".ti,ab OR "Respiration Rates".ti,ab OR exp *"physical capacity"/ OR "physical capacity".ti,ab OR exp *"Endurance"/ OR "Physical Endurance".ti,ab OR "endurance".ti,ab OR "stamina".ti,ab OR "Anaerobic Threshold".ti,ab OR "Exercise Tolerance".ti,ab OR exp *"Hand Strength"/ OR "Hand Strength".ti,ab OR "grip strength".ti,ab OR "pinch strength".ti,ab OR "pinch strengths".ti,ab OR "Hand Strengths".ti,ab OR "Grip".ti,ab OR "Grips".ti,ab OR "Grasp".ti,ab OR "Grasps".ti,ab OR exp *"muscle mass"/ OR "muscle mass".ti,ab OR exp *"Muscle Strength"/ OR "Muscle Strength".ti,ab OR exp *"Cognition"/ OR "Cognition".ti,ab OR "Cognit*".ti,ab OR "Awareness".ti,ab OR "Cognitive Dissonance".ti,ab OR "Cognitive Reserve".ti,ab OR "Comprehension".ti,ab OR "Consciousness".ti,ab OR "Imagination".ti,ab OR "Dreams".ti,ab OR "Fantasy".ti,ab OR "Intuition".ti,ab OR "Metacognition".ti,ab OR "Cognitive Rumination".ti,ab OR "cerebral function".ti,ab OR "cerebral functions".ti,ab OR *"Mental Function"/ OR exp *"Fatigue"/ OR exp *"Muscle Fatigue"/ OR "Fatigue".ti,ab OR "Fatigue*".ti,ab OR exp *"Quality of Life"/ OR "quality of life".ti,ab OR "QoL".ti,ab OR "life quality".ti,ab OR "HRQoL".ti,ab OR "PROM".ti,ab OR "PROMs".ti,ab OR *"Patient Reported-Outcome"/ OR "Patient Reported Outcome".ti,ab OR "Patient Reported Outcomes".ti,ab) AND **(exp** "**review"/ OR "review".mp OR "overview".mp OR "review*".mp OR "overview*".mp)** NOT ("acute anemia".ti OR "trauma".ti OR "trauma*".ti OR exp *"Injury"/ OR *"major surgery"/ OR "major surgery".ti OR "gastro-intestinal bleeding".ti OR "gastrointestinal bleeding".ti OR exp *"Gastrointestinal Hemorrhage"/ OR "Gastrointestinal Hemorrhage".ti OR "Gastrointestinal Haemorrhage".ti OR "Gastro intestinal Hemorrhage".ti OR "Gastro intestinal Haemorrhage".ti OR "glycosylated".ti OR exp *"Hemorrhagic Shock"/ OR "hemorrhagic shock".ti OR exp *"Transgender"/ OR "transsexual*".ti OR "trans sexual*".ti OR "transgender*".ti OR "trans gender*".ti OR "acute bleeding".ti OR "acute hemorrhag*".ti OR "acute haemorrhag*".ti) NOT (conference review or conference abstract).pt)

Sub-vraag 1:

(("chronic anaemia".ti,ab OR "chronic anaemi*".ti,ab OR "chronic anemia".ti,ab OR "chronic anemi*".ti,ab OR ((exp *"Anemia"/ OR "Anemia".ti,ab OR "Anaemia".ti,ab OR "Anemi*".ti,ab OR "Anaemi*".ti,ab) AND (exp *"Chronic Disease"/ OR "Chronic".ti,ab OR "Chronic*".ti,ab)) OR ((exp *"Anemia"/ OR "Anemia".ti,ab OR "Anaemia".ti,ab OR "Anemi*".ti,ab OR "Anaemi*".ti,ab) AND (exp *"Hemodialysis"/ OR "Renal Dialysis".ti,ab OR "Hemodialysis".ti,ab OR "Haemodialysis".ti,ab)) OR ((exp *"Anemia"/ OR "Anemia".ti,ab OR "Anaemia".ti,ab OR "Anemi*".ti,ab OR "Anaemi*".ti,ab) AND (exp *"Myelodysplastic Syndrome"/ OR "Myelodysplastic Syndromes".ti,ab OR "Myelodysplastic Syndromes".ti,ab OR "MDS".ti,ab)) OR ((exp *"Anemia"/ OR "Anemia".ti,ab OR "Anaemia".ti,ab OR "Anemi*".ti,ab OR "Anaemi*".ti,ab) AND (exp *"myeloproliferative neoplasm"/ OR "myeloproliferative neoplasms".ti,ab OR "myeloproliferative neoplasms".ti,ab OR "myeloproliferative neoplasia".ti,ab OR "MPN".ti,ab OR "MPNs".ti,ab)) OR exp *"Sickle Cell Anemia"/ OR "sickle cell anemia".ti,ab OR "sickle cell anemi*".ti,ab OR "sickle cell anaemia".ti,ab OR "sickle cell anaemi*".ti,ab OR "sickle cell disease".ti,ab OR "Acute Chest Syndrome".ti,ab OR "Haemoglobin SC Disease".ti,ab OR "Hemoglobin SC Disease".ti,ab OR "Sickle Cell Trait".ti,ab OR exp *"Thalassemia"/ OR "Thalassemia".ti,ab OR "Thalassemi*".ti,ab OR exp *"Aplastic Anemia"/ OR "aplastic anemia".ti,ab OR "aplastic anemi*".ti,ab OR "aplastic anaemia".ti,ab OR "aplastic anaemi*".ti,ab OR "fanconi anemia".ti,ab OR "fanconi anemi*".ti,ab OR "fanconi anaemia".ti,ab OR "fanconi anaemi*".ti,ab OR "diamond-blackfan anemia".ti,ab OR "diamond-blackfan anemi*".ti,ab OR "diamond-blackfan anaemia".ti,ab OR "diamond-blackfan anaemi*".ti,ab OR "congenital hypoplastic anemia".ti,ab OR "congenital hypoplastic anemi*".ti,ab OR "congenital hypoplastic anaemia".ti,ab OR "congenital hypoplastic anaemi*".ti,ab) AND (exp *"Erythropoietin"/ OR "Erythropoietin".ti,ab OR "Erythropoietin*".ti,ab OR "EPO".ti,ab OR "epoetin".ti,ab OR "liberal transfusion strategy".ti,ab OR "liberal transfusion".ti,ab OR "higher nadir".ti,ab OR "higher nadir*".ti,ab OR "higher threshold".ti,ab OR "higher threshold*".ti,ab OR "iron therapy".ti,ab OR "iron therap*".ti,ab OR "iron treatment".ti,ab OR "iron treat*".ti,ab OR exp *"Iron therapy"/ OR "altitude training".ti,ab OR ((exp *"Altitude"/ OR "altitude".ti,ab OR "altitude*".ti,ab) AND ("training".ti,ab OR exp *"Exercise"/ OR exp *"Training"/ OR "exercise".ti,ab))) AND ("VO2max".ti,ab OR "VO2 max".ti,ab OR "VO2max*".ti,ab OR "VO2 max*".ti,ab OR "maximal oxygen uptake".ti,ab OR "maximal oxygen consumption".ti,ab OR exp *"Maximal Oxygen Uptake"/ OR exp *"Heart Output"/ OR "Cardiac output".ti,ab OR "stroke volume".ti,ab OR exp *"Heart Rate"/ OR "Heart rate".ti,ab OR "Heart Rates".ti,ab OR "Cardiac Rate".ti,ab OR "Cardiac Rates".ti,ab OR "Pulse Rate".ti,ab OR "Pulse Rates".ti,ab OR "Heartbeat".ti,ab OR "Heartbeats".ti,ab OR "Cardiac Chronotropy".ti,ab OR "Cardiac Chronotropism".ti,ab OR exp *"Breathing Rate"/ OR "Respiratory rate".ti,ab OR "Respiratory Rates".ti,ab OR "Respiration Rate".ti,ab OR "Respiration Rates".ti,ab OR exp *"physical capacity"/ OR "physical capacity".ti,ab OR exp *"Endurance"/ OR "Physical Endurance".ti,ab OR "endurance".ti,ab OR "stamina".ti,ab OR "Anaerobic Threshold".ti,ab OR "Exercise Tolerance".ti,ab OR exp *"Hand Strength"/ OR "Hand Strength".ti,ab OR "grip strength".ti,ab OR "pinch strength".ti,ab OR "pinch strengths".ti,ab OR "Hand Strengths".ti,ab OR "Grip".ti,ab OR "Grips".ti,ab OR "Grasp".ti,ab OR "Grasps".ti,ab OR exp *"muscle mass"/ OR "muscle mass".ti,ab OR exp *"Muscle Strength"/ OR "Muscle Strength".ti,ab OR exp *"Cognition"/ OR "Cognition".ti,ab OR "Cognit*".ti,ab OR "Awareness".ti,ab OR "Cognitive Dissonance".ti,ab OR "Cognitive Reserve".ti,ab OR "Comprehension".ti,ab OR "Consciousness".ti,ab OR "Imagination".ti,ab OR "Dreams".ti,ab OR "Fantasy".ti,ab OR "Intuition".ti,ab OR "Metacognition".ti,ab OR "Cognitive Rumination".ti,ab OR "cerebral function".ti,ab OR "cerebral functions".ti,ab OR *"Mental Function"/ OR exp *"Fatigue"/ OR exp *"Muscle Fatigue"/ OR "Fatigue".ti,ab OR "Fatigue*".ti,ab OR exp *"Quality of Life"/ OR "quality of life".ti,ab OR "QoL".ti,ab OR "life quality".ti,ab OR "HRQoL".ti,ab OR "PROM".ti,ab OR "PROMs".ti,ab OR *"Patient Reported-Outcome"/ OR "Patient Reported Outcome".ti,ab OR "Patient Reported Outcomes".ti,ab) AND **(exp** "**review"/ OR "review".mp OR "overview".mp OR "review*".mp OR "overview*".mp)** NOT ("acute anemia".ti OR "trauma".ti OR "trauma*".ti OR exp *"Injury"/ OR *"major surgery"/ OR "major surgery".ti OR "gastro-intestinal bleeding".ti OR "gastrointestinal bleeding".ti OR exp *"Gastrointestinal Hemorrhage"/ OR "Gastrointestinal Hemorrhage".ti OR "Gastrointestinal Haemorrhage".ti OR "Gastro intestinal Hemorrhage".ti OR "Gastro intestinal Haemorrhage".ti OR "glycosylated".ti OR exp *"Hemorrhagic Shock"/ OR "hemorrhagic shock".ti OR exp *"Transgender"/ OR "transsexual*".ti OR "trans sexual*".ti OR "transgender*".ti OR "trans gender*".ti OR "acute bleeding".ti OR "acute hemorrhag*".ti OR "acute haemorrhag*".ti) NOT (conference review or conference abstract).pt)

Sub-vraag 2:

((*"Normal Human"/ OR "Healthy Participant".ti,ab OR "Healthy Participants".ti,ab OR "Healthy Subject".ti,ab OR "Healthy Subjects".ti,ab OR "Healthy Volunteer".ti,ab OR "Human Volunteer".ti,ab OR "Human Volunteers".ti,ab OR "Normal Volunteer".ti,ab OR "Normal Volunteers".ti,ab OR exp *"Athlete"/ OR "Athletes".ti,ab OR "Athlete".ti,ab OR "fit patients".ti,ab OR "fit patient".ti,ab) AND (exp *"Erythropoietin"/ OR "Erythropoietin".ti,ab OR "Erythropoietin*".ti,ab OR "EPO".ti,ab OR "epoetin".ti,ab OR "liberal transfusion strategy".ti,ab OR "liberal transfusion".ti,ab OR "higher nadir".ti,ab OR "higher nadir*".ti,ab OR "higher threshold".ti,ab OR "higher threshold*".ti,ab OR "iron therapy".ti,ab OR "iron therap*".ti,ab OR "iron treatment".ti,ab OR "iron treat*".ti,ab OR exp *"Iron therapy"/ OR "altitude training".ti,ab OR ((exp *"Altitude"/ OR "altitude".ti,ab OR "altitude*".ti,ab) AND ("training".ti,ab OR exp *"Exercise"/ OR exp *"Training"/ OR "exercise".ti,ab))) AND ("VO2max".ti,ab OR "VO2 max".ti,ab OR "VO2max*".ti,ab OR "VO2 max*".ti,ab OR "maximal oxygen uptake".ti,ab OR "maximal oxygen consumption".ti,ab OR exp *"Maximal Oxygen Uptake"/ OR exp *"Heart Output"/ OR "Cardiac output".ti,ab OR "stroke volume".ti,ab OR exp *"Heart Rate"/ OR "Heart rate".ti,ab OR "Heart Rates".ti,ab OR "Cardiac Rate".ti,ab OR "Cardiac Rates".ti,ab OR "Pulse Rate".ti,ab OR "Pulse Rates".ti,ab OR "Heartbeat".ti,ab OR "Heartbeats".ti,ab OR "Cardiac Chronotropy".ti,ab OR "Cardiac Chronotropism".ti,ab OR exp *"Breathing Rate"/ OR "Respiratory rate".ti,ab OR "Respiratory Rates".ti,ab OR "Respiration Rate".ti,ab OR "Respiration Rates".ti,ab OR exp *"physical capacity"/ OR "physical capacity".ti,ab OR exp *"Endurance"/ OR "Physical Endurance".ti,ab OR "endurance".ti,ab OR "stamina".ti,ab OR "Anaerobic Threshold".ti,ab OR "Exercise Tolerance".ti,ab OR exp *"Hand Strength"/ OR "Hand Strength".ti,ab OR "grip strength".ti,ab OR "pinch strength".ti,ab OR "pinch strengths".ti,ab OR "Hand Strengths".ti,ab OR "Grip".ti,ab OR "Grips".ti,ab OR "Grasp".ti,ab OR "Grasps".ti,ab OR exp *"muscle mass"/ OR "muscle mass".ti,ab OR exp *"Muscle Strength"/ OR "Muscle Strength".ti,ab OR exp *"Cognition"/ OR "Cognition".ti,ab OR "Cognit*".ti,ab OR "Awareness".ti,ab OR "Cognitive Dissonance".ti,ab OR "Cognitive Reserve".ti,ab OR "Comprehension".ti,ab OR "Consciousness".ti,ab OR "Imagination".ti,ab OR "Dreams".ti,ab OR "Fantasy".ti,ab OR "Intuition".ti,ab OR "Metacognition".ti,ab OR "Cognitive Rumination".ti,ab OR "cerebral function".ti,ab OR "cerebral functions".ti,ab OR *"Mental Function"/ OR exp *"Fatigue"/ OR exp *"Muscle Fatigue"/ OR "Fatigue".ti,ab OR "Fatigue*".ti,ab OR exp *"Quality of Life"/ OR "quality of life".ti,ab OR "QoL".ti,ab OR "life quality".ti,ab OR "HRQoL".ti,ab OR "PROM".ti,ab OR "PROMs".ti,ab OR *"Patient Reported-Outcome"/ OR "Patient Reported Outcome".ti,ab OR "Patient Reported Outcomes".ti,ab) AND **(exp** "**review"/ OR "review".mp OR "overview".mp OR "review*".mp OR "overview*".mp)** NOT ("acute anemia".ti OR "trauma".ti OR "trauma*".ti OR exp *"Injury"/ OR *"major surgery"/ OR "major surgery".ti OR "gastro-intestinal bleeding".ti OR "gastrointestinal bleeding".ti OR exp *"Gastrointestinal Hemorrhage"/ OR "Gastrointestinal Hemorrhage".ti OR "Gastrointestinal Haemorrhage".ti OR "Gastro intestinal Hemorrhage".ti OR "Gastro intestinal Haemorrhage".ti OR "glycosylated".ti OR exp *"Hemorrhagic Shock"/ OR "hemorrhagic shock".ti OR exp *"Transgender"/ OR "transsexual*".ti OR "trans sexual*".ti OR "transgender*".ti OR "trans gender*".ti OR "acute bleeding".ti OR "acute hemorrhag*".ti OR "acute haemorrhag*".ti) NOT (conference review or conference abstract).pt)

Sub-vraag 3:

(("Erythrocythemia".ti,ab OR "Erythrocythem*".ti,ab OR "Erythrocythaemia".ti,ab OR "Erythrocythaem*".ti,ab OR "Erythrocytemia".ti,ab OR "Erythrocytem*".ti,ab OR "Erythrocytaemia".ti,ab OR "Erythrocytaem*".ti,ab OR "Polycythemia Vera"/ OR exp *"Polycythemia"/ OR "Polycythemia".ti,ab OR "Polycythemi*".ti,ab OR "Polycythaemia".ti,ab OR "Polycythaemi*".ti,ab OR "erythrocytosis".ti,ab OR "erytrocytosis".ti,ab) AND (exp *"Phlebotomy"/ OR "Phlebotomy".ti,ab OR "Phlebotom*".ti,ab OR "Venesection*".ti,ab OR "Venipuncture*".ti,ab OR exp *"Hydroxyurea"/ OR "Hydroxyurea".ti,ab OR "Hydroxyurea*".ti,ab OR "hydrea".ti,ab OR "Hydroxycarbamid*".ti,ab OR "Oncocarbide*".ti,ab OR exp *"Busulfan"/ OR "Busulfan".ti,ab OR "Busulfan*".ti,ab OR "Busulphan".ti,ab OR "Busulfex".ti,ab OR "Myleran".ti,ab OR "Myelosan".ti,ab OR "Mylecytan".ti,ab OR "Glyzophrol".ti,ab OR exp *"Interferon"/ OR "Interferons".ti,ab OR "Interferon".ti,ab OR "Interferon*".ti,ab) AND ("VO2max".ti,ab OR "VO2 max".ti,ab OR "VO2max*".ti,ab OR "VO2 max*".ti,ab OR "maximal oxygen uptake".ti,ab OR "maximal oxygen consumption".ti,ab OR exp *"Maximal Oxygen Uptake"/ OR exp *"Heart Output"/ OR "Cardiac output".ti,ab OR "stroke volume".ti,ab OR exp *"Heart Rate"/ OR "Heart rate".ti,ab OR "Heart Rates".ti,ab OR "Cardiac Rate".ti,ab OR "Cardiac Rates".ti,ab OR "Pulse Rate".ti,ab OR "Pulse Rates".ti,ab OR "Heartbeat".ti,ab OR "Heartbeats".ti,ab OR "Cardiac Chronotropy".ti,ab OR "Cardiac Chronotropism".ti,ab OR exp *"Breathing Rate"/ OR "Respiratory rate".ti,ab OR "Respiratory Rates".ti,ab OR "Respiration Rate".ti,ab OR "Respiration Rates".ti,ab OR exp *"physical capacity"/ OR "physical capacity".ti,ab OR exp *"Endurance"/ OR "Physical Endurance".ti,ab OR "endurance".ti,ab OR "stamina".ti,ab OR "Anaerobic Threshold".ti,ab OR "Exercise Tolerance".ti,ab OR exp *"Hand Strength"/ OR "Hand Strength".ti,ab OR "grip strength".ti,ab OR "pinch strength".ti,ab OR "pinch strengths".ti,ab OR "Hand Strengths".ti,ab OR "Grip".ti,ab OR "Grips".ti,ab OR "Grasp".ti,ab OR "Grasps".ti,ab OR exp *"muscle mass"/ OR "muscle mass".ti,ab OR exp *"Muscle Strength"/ OR "Muscle Strength".ti,ab OR exp *"Cognition"/ OR "Cognition".ti,ab OR "Cognit*".ti,ab OR "Awareness".ti,ab OR "Cognitive Dissonance".ti,ab OR "Cognitive Reserve".ti,ab OR "Comprehension".ti,ab OR "Consciousness".ti,ab OR "Imagination".ti,ab OR "Dreams".ti,ab OR "Fantasy".ti,ab OR "Intuition".ti,ab OR "Metacognition".ti,ab OR "Cognitive Rumination".ti,ab OR "cerebral function".ti,ab OR "cerebral functions".ti,ab OR *"Mental Function"/ OR exp *"Fatigue"/ OR exp *"Muscle Fatigue"/ OR "Fatigue".ti,ab OR "Fatigue*".ti,ab OR exp *"Quality of Life"/ OR "quality of life".ti,ab OR "QoL".ti,ab OR "life quality".ti,ab OR "HRQoL".ti,ab OR "PROM".ti,ab OR "PROMs".ti,ab OR *"Patient Reported-Outcome"/ OR "Patient Reported Outcome".ti,ab OR "Patient Reported Outcomes".ti,ab) AND **(exp** "**review"/ OR "review".mp OR "overview".mp OR "review*".mp OR "overview*".mp)** NOT ("acute anemia".ti OR "trauma".ti OR "trauma*".ti OR exp *"Injury"/ OR *"major surgery"/ OR "major surgery".ti OR "gastro-intestinal bleeding".ti OR "gastrointestinal bleeding".ti OR exp *"Gastrointestinal Hemorrhage"/ OR "Gastrointestinal Hemorrhage".ti OR "Gastrointestinal Haemorrhage".ti OR "Gastro intestinal Hemorrhage".ti OR "Gastro intestinal Haemorrhage".ti OR "glycosylated".ti OR exp *"Hemorrhagic Shock"/ OR "hemorrhagic shock".ti OR exp *"Transgender"/ OR "transsexual*".ti OR "trans sexual*".ti OR "transgender*".ti OR "trans gender*".ti OR "acute bleeding".ti OR "acute hemorrhag*".ti OR "acute haemorrhag*".ti) NOT (conference review or conference abstract).pt)

**Web of Science**

<http://isiknowledge.com/wos>

Basisvraag:

- met nabijheidsoperator Adjacency

TS=(("Hemoglobins" OR "Haemoglobins" OR "Hemoglobin" OR "Haemoglobin" OR "Hemoglobin*" OR "Haemoglobin*" OR "alpha-Globin" OR "alpha-Globin*" OR "alpha-Globins" OR "beta-Globin" OR "beta-Globin*" OR "beta-Globins" OR "Carboxyhemoglobin" OR "Carboxyhemoglobin*" OR "Carboxyhemoglobins" OR "delta-Globin" OR "delta-Globin*" OR "delta-Globins" OR "epsilon-Globin" OR "epsilon-Globin*" OR "epsilon-Globins" OR "Erythrocruorin" OR "Erythrocruorin*" OR "Erythrocruorins" OR "gamma-Globin" OR "gamma-Globin*" OR "gamma-Globins" OR "Methemoglobin" OR "Methemoglobin*" OR "Methemoglobins" OR "Oxyhemoglobin" OR "Oxyhemoglobin*" OR "Oxyhemoglobins" OR "Sulfhemoglobin" OR "Sulfhemoglobin*" OR "Sulfhemoglobins" OR "zeta-Globin" OR "zeta-Globin*" OR "zeta-Globins") **NEAR/4** ("mass" OR "concentration" OR "concentrations" OR "higher hemoglobin*" OR "higher haemoglobin*" OR "lower hemoglobin*" OR "lower haemoglobin*" OR "hemoglobin increas*" OR "haemoglobin increas*" OR "hemoglobin decreas*" OR "haemoglobin decreas*" OR "hemoglobin chang*" OR "haemoglobin chang*" OR "hemoglobin volume" OR "haemoglobin volume") AND ("VO2max" OR "VO2 max" OR "VO2max*" OR "VO2 max*" OR "maximal oxygen uptake" OR "maximal oxygen consumption" OR "Maximal Oxygen Uptake" OR "Heart Output" OR "Cardiac output" OR "stroke volume" OR "Heart Rate" OR "Heart rate" OR "Heart Rates" OR "Cardiac Rate" OR "Cardiac Rates" OR "Pulse Rate" OR "Pulse Rates" OR "Heartbeat" OR "Heartbeats" OR "Cardiac Chronotropy" OR "Cardiac Chronotropism" OR "Breathing Rate" OR "Respiratory rate" OR "Respiratory Rates" OR "Respiration Rate" OR "Respiration Rates" OR "physical capacity" OR "physical capacity" OR "Endurance" OR "Physical Endurance" OR "endurance" OR "stamina" OR "Anaerobic Threshold" OR "Exercise Tolerance" OR "Hand Strength" OR "Hand Strength" OR "grip strength" OR "pinch strength" OR "pinch strengths" OR "Hand Strengths" OR "Grip" OR "Grips" OR "Grasp" OR "Grasps" OR "muscle mass" OR "muscle mass" OR "Muscle Strength" OR "Muscle Strength" OR "Cognition" OR "Cognition" OR "Cognit*" OR "Awareness" OR "Cognitive Dissonance" OR "Cognitive Reserve" OR "Comprehension" OR "Consciousness" OR "Imagination" OR "Dreams" OR "Fantasy" OR "Intuition" OR "Metacognition" OR "Cognitive Rumination" OR "cerebral function" OR "cerebral functions" OR "Mental Function" OR "Fatigue" OR "Muscle Fatigue" OR "Fatigue" OR "Fatigue*" OR "Quality of Life" OR "quality of life" OR "QoL" OR "life quality" OR "HRQoL" OR "PROM" OR "PROMs" OR "Patient Reported-Outcome" OR "Patient Reported Outcome" OR "Patient Reported Outcomes") AND **(**"**review" OR "review" OR "overview" OR "review*" OR "overview*"))** NOT TI=("acute anemia" OR "trauma" OR "trauma*" OR "Injury" OR "major surgery" OR "major surgery" OR "gastro-intestinal bleeding" OR "gastrointestinal bleeding" OR "Gastrointestinal Hemorrhage" OR "Gastrointestinal Hemorrhage" OR "Gastrointestinal Haemorrhage" OR "Gastro intestinal Hemorrhage" OR "Gastro intestinal Haemorrhage" OR "glycosylated" OR "Hemorrhagic Shock" OR "hemorrhagic shock" OR "Transgender" OR "transsexual*" OR "trans sexual*" OR "transgender*" OR "trans gender*" OR "acute bleeding" OR "acute hemorrhag*" OR "acute haemorrhag*") NOT DT=(meeting abstract)

Sub-vraag 1:

TS=(("chronic anaemia" OR "chronic anaemi*" OR "chronic anemia" OR "chronic anemi*" OR (("Anemia" OR "Anemia" OR "Anaemia" OR "Anemi*" OR "Anaemi*") AND ("Chronic Disease" OR "Chronic" OR "Chronic*")) OR (("Anemia" OR "Anemia" OR "Anaemia" OR "Anemi*" OR "Anaemi*") AND ("Hemodialysis" OR "Renal Dialysis" OR "Hemodialysis" OR "Haemodialysis")) OR (("Anemia" OR "Anemia" OR "Anaemia" OR "Anemi*" OR "Anaemi*") AND ("Myelodysplastic Syndrome" OR "Myelodysplastic Syndromes" OR "Myelodysplastic Syndromes" OR "MDS")) OR (("Anemia" OR "Anemia" OR "Anaemia" OR "Anemi*" OR "Anaemi*") AND ("myeloproliferative neoplasm" OR "myeloproliferative neoplasms" OR "myeloproliferative neoplasms" OR "myeloproliferative neoplasia" OR "MPN" OR "MPNs")) OR "Sickle Cell Anemia" OR "sickle cell anemia" OR "sickle cell anemi*" OR "sickle cell anaemia" OR "sickle cell anaemi*" OR "sickle cell disease" OR "Acute Chest Syndrome" OR "Haemoglobin SC Disease" OR "Hemoglobin SC Disease" OR "Sickle Cell Trait" OR "Thalassemia" OR "Thalassemia" OR "Thalassemi*" OR "Aplastic Anemia" OR "aplastic anemia" OR "aplastic anemi*" OR "aplastic anaemia" OR "aplastic anaemi*" OR "fanconi anemia" OR "fanconi anemi*" OR "fanconi anaemia" OR "fanconi anaemi*" OR "diamond-blackfan anemia" OR "diamond-blackfan anemi*" OR "diamond-blackfan anaemia" OR "diamond-blackfan anaemi*" OR "congenital hypoplastic anemia" OR "congenital hypoplastic anemi*" OR "congenital hypoplastic anaemia" OR "congenital hypoplastic anaemi*") AND ("Erythropoietin" OR "Erythropoietin" OR "Erythropoietin*" OR "EPO" OR "epoetin" OR "liberal transfusion strategy" OR "liberal transfusion" OR "higher nadir" OR "higher nadir*" OR "higher threshold" OR "higher threshold*" OR "iron therapy" OR "iron therap*" OR "iron treatment" OR "iron treat*" OR "Iron therapy" OR "altitude training" OR (("Altitude" OR "altitude" OR "altitude*") AND ("training" OR "Exercise" OR "Training" OR "exercise"))) AND ("VO2max" OR "VO2 max" OR "VO2max*" OR "VO2 max*" OR "maximal oxygen uptake" OR "maximal oxygen consumption" OR "Maximal Oxygen Uptake" OR "Heart Output" OR "Cardiac output" OR "stroke volume" OR "Heart Rate" OR "Heart rate" OR "Heart Rates" OR "Cardiac Rate" OR "Cardiac Rates" OR "Pulse Rate" OR "Pulse Rates" OR "Heartbeat" OR "Heartbeats" OR "Cardiac Chronotropy" OR "Cardiac Chronotropism" OR "Breathing Rate" OR "Respiratory rate" OR "Respiratory Rates" OR "Respiration Rate" OR "Respiration Rates" OR "physical capacity" OR "physical capacity" OR "Endurance" OR "Physical Endurance" OR "endurance" OR "stamina" OR "Anaerobic Threshold" OR "Exercise Tolerance" OR "Hand Strength" OR "Hand Strength" OR "grip strength" OR "pinch strength" OR "pinch strengths" OR "Hand Strengths" OR "Grip" OR "Grips" OR "Grasp" OR "Grasps" OR "muscle mass" OR "muscle mass" OR "Muscle Strength" OR "Muscle Strength" OR "Cognition" OR "Cognition" OR "Cognit*" OR "Awareness" OR "Cognitive Dissonance" OR "Cognitive Reserve" OR "Comprehension" OR "Consciousness" OR "Imagination" OR "Dreams" OR "Fantasy" OR "Intuition" OR "Metacognition" OR "Cognitive Rumination" OR "cerebral function" OR "cerebral functions" OR "Mental Function" OR "Fatigue" OR "Muscle Fatigue" OR "Fatigue" OR "Fatigue*" OR "Quality of Life" OR "quality of life" OR "QoL" OR "life quality" OR "HRQoL" OR "PROM" OR "PROMs" OR "Patient Reported-Outcome" OR "Patient Reported Outcome" OR "Patient Reported Outcomes") AND **(**"**review" OR "review" OR "overview" OR "review*" OR "overview*"))** NOT TI=("acute anemia" OR "trauma" OR "trauma*" OR "Injury" OR "major surgery" OR "major surgery" OR "gastro-intestinal bleeding" OR "gastrointestinal bleeding" OR "Gastrointestinal Hemorrhage" OR "Gastrointestinal Hemorrhage" OR "Gastrointestinal Haemorrhage" OR "Gastro intestinal Hemorrhage" OR "Gastro intestinal Haemorrhage" OR "glycosylated" OR "Hemorrhagic Shock" OR "hemorrhagic shock" OR "Transgender" OR "transsexual*" OR "trans sexual*" OR "transgender*" OR "trans gender*" OR "acute bleeding" OR "acute hemorrhag*" OR "acute haemorrhag*") NOT DT=(meeting abstract)

Sub-vraag 2:

TS=(("Normal Human" OR "Healthy Participant" OR "Healthy Participants" OR "Healthy Subject" OR "Healthy Subjects" OR "Healthy Volunteer" OR "Human Volunteer" OR "Human Volunteers" OR "Normal Volunteer" OR "Normal Volunteers" OR "Athlete" OR "Athletes" OR "Athlete" OR "fit patients" OR "fit patient") AND ("Erythropoietin" OR "Erythropoietin" OR "Erythropoietin*" OR "EPO" OR "epoetin" OR "liberal transfusion strategy" OR "liberal transfusion" OR "higher nadir" OR "higher nadir*" OR "higher threshold" OR "higher threshold*" OR "iron therapy" OR "iron therap*" OR "iron treatment" OR "iron treat*" OR "Iron therapy" OR "altitude training" OR (("Altitude" OR "altitude" OR "altitude*") AND ("training" OR "Exercise" OR "Training" OR "exercise"))) AND ("VO2max" OR "VO2 max" OR "VO2max*" OR "VO2 max*" OR "maximal oxygen uptake" OR "maximal oxygen consumption" OR "Maximal Oxygen Uptake" OR "Heart Output" OR "Cardiac output" OR "stroke volume" OR "Heart Rate" OR "Heart rate" OR "Heart Rates" OR "Cardiac Rate" OR "Cardiac Rates" OR "Pulse Rate" OR "Pulse Rates" OR "Heartbeat" OR "Heartbeats" OR "Cardiac Chronotropy" OR "Cardiac Chronotropism" OR "Breathing Rate" OR "Respiratory rate" OR "Respiratory Rates" OR "Respiration Rate" OR "Respiration Rates" OR "physical capacity" OR "physical capacity" OR "Endurance" OR "Physical Endurance" OR "endurance" OR "stamina" OR "Anaerobic Threshold" OR "Exercise Tolerance" OR "Hand Strength" OR "Hand Strength" OR "grip strength" OR "pinch strength" OR "pinch strengths" OR "Hand Strengths" OR "Grip" OR "Grips" OR "Grasp" OR "Grasps" OR "muscle mass" OR "muscle mass" OR "Muscle Strength" OR "Muscle Strength" OR "Cognition" OR "Cognition" OR "Cognit*" OR "Awareness" OR "Cognitive Dissonance" OR "Cognitive Reserve" OR "Comprehension" OR "Consciousness" OR "Imagination" OR "Dreams" OR "Fantasy" OR "Intuition" OR "Metacognition" OR "Cognitive Rumination" OR "cerebral function" OR "cerebral functions" OR "Mental Function" OR "Fatigue" OR "Muscle Fatigue" OR "Fatigue" OR "Fatigue*" OR "Quality of Life" OR "quality of life" OR "QoL" OR "life quality" OR "HRQoL" OR "PROM" OR "PROMs" OR "Patient Reported-Outcome" OR "Patient Reported Outcome" OR "Patient Reported Outcomes") AND **(**"**review" OR "review" OR "overview" OR "review*" OR "overview*"))** NOT TI=("acute anemia" OR "trauma" OR "trauma*" OR "Injury" OR "major surgery" OR "major surgery" OR "gastro-intestinal bleeding" OR "gastrointestinal bleeding" OR "Gastrointestinal Hemorrhage" OR "Gastrointestinal Hemorrhage" OR "Gastrointestinal Haemorrhage" OR "Gastro intestinal Hemorrhage" OR "Gastro intestinal Haemorrhage" OR "glycosylated" OR "Hemorrhagic Shock" OR "hemorrhagic shock" OR "Transgender" OR "transsexual*" OR "trans sexual*" OR "transgender*" OR "trans gender*" OR "acute bleeding" OR "acute hemorrhag*" OR "acute haemorrhag*") NOT DT=(meeting abstract)

Sub-vraag 3:

TS=(("Erythrocythemia" OR "Erythrocythem*" OR "Erythrocythaemia" OR "Erythrocythaem*" OR "Erythrocytemia" OR "Erythrocytem*" OR "Erythrocytaemia" OR "Erythrocytaem*" OR "Polycythemia Vera" OR "Polycythemia" OR "Polycythemia" OR "Polycythemi*" OR "Polycythaemia" OR "Polycythaemi*" OR "erythrocytosis" OR "erytrocytosis") AND ("Phlebotomy" OR "Phlebotomy" OR "Phlebotom*" OR "Venesection*" OR "Venipuncture*" OR "Hydroxyurea" OR "Hydroxyurea" OR "Hydroxyurea*" OR "hydrea" OR "Hydroxycarbamid*" OR "Oncocarbide*" OR "Busulfan" OR "Busulfan" OR "Busulfan*" OR "Busulphan" OR "Busulfex" OR "Myleran" OR "Myelosan" OR "Mylecytan" OR "Glyzophrol" OR "Interferon" OR "Interferons" OR "Interferon" OR "Interferon*") AND ("VO2max" OR "VO2 max" OR "VO2max*" OR "VO2 max*" OR "maximal oxygen uptake" OR "maximal oxygen consumption" OR "Maximal Oxygen Uptake" OR "Heart Output" OR "Cardiac output" OR "stroke volume" OR "Heart Rate" OR "Heart rate" OR "Heart Rates" OR "Cardiac Rate" OR "Cardiac Rates" OR "Pulse Rate" OR "Pulse Rates" OR "Heartbeat" OR "Heartbeats" OR "Cardiac Chronotropy" OR "Cardiac Chronotropism" OR "Breathing Rate" OR "Respiratory rate" OR "Respiratory Rates" OR "Respiration Rate" OR "Respiration Rates" OR "physical capacity" OR "physical capacity" OR "Endurance" OR "Physical Endurance" OR "endurance" OR "stamina" OR "Anaerobic Threshold" OR "Exercise Tolerance" OR "Hand Strength" OR "Hand Strength" OR "grip strength" OR "pinch strength" OR "pinch strengths" OR "Hand Strengths" OR "Grip" OR "Grips" OR "Grasp" OR "Grasps" OR "muscle mass" OR "muscle mass" OR "Muscle Strength" OR "Muscle Strength" OR "Cognition" OR "Cognition" OR "Cognit*" OR "Awareness" OR "Cognitive Dissonance" OR "Cognitive Reserve" OR "Comprehension" OR "Consciousness" OR "Imagination" OR "Dreams" OR "Fantasy" OR "Intuition" OR "Metacognition" OR "Cognitive Rumination" OR "cerebral function" OR "cerebral functions" OR "Mental Function" OR "Fatigue" OR "Muscle Fatigue" OR "Fatigue" OR "Fatigue*" OR "Quality of Life" OR "quality of life" OR "QoL" OR "life quality" OR "HRQoL" OR "PROM" OR "PROMs" OR "Patient Reported-Outcome" OR "Patient Reported Outcome" OR "Patient Reported Outcomes") AND **(**"**review" OR "review" OR "overview" OR "review*" OR "overview*"))** NOT TI=("acute anemia" OR "trauma" OR "trauma*" OR "Injury" OR "major surgery" OR "major surgery" OR "gastro-intestinal bleeding" OR "gastrointestinal bleeding" OR "Gastrointestinal Hemorrhage" OR "Gastrointestinal Hemorrhage" OR "Gastrointestinal Haemorrhage" OR "Gastro intestinal Hemorrhage" OR "Gastro intestinal Haemorrhage" OR "glycosylated" OR "Hemorrhagic Shock" OR "hemorrhagic shock" OR "Transgender" OR "transsexual*" OR "trans sexual*" OR "transgender*" OR "trans gender*" OR "acute bleeding" OR "acute hemorrhag*" OR "acute haemorrhag*") NOT DT=(meeting abstract)

**Cochrane**

<https://www.cochranelibrary.com/advanced-search/search-manager>

Alleen Cochrane Reviews

Basisvraag:

- met nabijheidsoperator Adjacency

(("Hemoglobins" OR "Haemoglobins" OR "Hemoglobin" OR "Haemoglobin" OR "Hemoglobin*" OR "Haemoglobin*" OR "alpha Globin" OR "alpha Globin*" OR "alpha Globins" OR "beta Globin" OR "beta Globin*" OR "beta Globins" OR "Carboxyhemoglobin" OR "Carboxyhemoglobin*" OR "Carboxyhemoglobins" OR "delta Globin" OR "delta Globin*" OR "delta Globins" OR "epsilon Globin" OR "epsilon Globin*" OR "epsilon Globins" OR "Erythrocruorin" OR "Erythrocruorin*" OR "Erythrocruorins" OR "gamma Globin" OR "gamma Globin*" OR "gamma Globins" OR "Methemoglobin" OR "Methemoglobin*" OR "Methemoglobins" OR "Oxyhemoglobin" OR "Oxyhemoglobin*" OR "Oxyhemoglobins" OR "Sulfhemoglobin" OR "Sulfhemoglobin*" OR "Sulfhemoglobins" OR "zeta Globin" OR "zeta Globin*" OR "zeta Globins") **NEAR/4** ("mass" OR "concentration" OR "concentrations" OR "higher hemoglobin*" OR "higher haemoglobin*" OR "lower hemoglobin*" OR "lower haemoglobin*" OR "hemoglobin increas*" OR "haemoglobin increas*" OR "hemoglobin decreas*" OR "haemoglobin decreas*" OR "hemoglobin chang*" OR "haemoglobin chang*" OR "hemoglobin volume" OR "haemoglobin volume") AND ("VO2max" OR "VO2 max" OR "VO2max*" OR "VO2 max*" OR "maximal oxygen uptake" OR "maximal oxygen consumption" OR "Maximal Oxygen Uptake" OR "Heart Output" OR "Cardiac output" OR "stroke volume" OR "Heart Rate" OR "Heart rate" OR "Heart Rates" OR "Cardiac Rate" OR "Cardiac Rates" OR "Pulse Rate" OR "Pulse Rates" OR "Heartbeat" OR "Heartbeats" OR "Cardiac Chronotropy" OR "Cardiac Chronotropism" OR "Breathing Rate" OR "Respiratory rate" OR "Respiratory Rates" OR "Respiration Rate" OR "Respiration Rates" OR "physical capacity" OR "physical capacity" OR "Endurance" OR "Physical Endurance" OR "endurance" OR "stamina" OR "Anaerobic Threshold" OR "Exercise Tolerance" OR "Hand Strength" OR "Hand Strength" OR "grip strength" OR "pinch strength" OR "pinch strengths" OR "Hand Strengths" OR "Grip" OR "Grips" OR "Grasp" OR "Grasps" OR "muscle mass" OR "muscle mass" OR "Muscle Strength" OR "Muscle Strength" OR "Cognition" OR "Cognition" OR "Cognit*" OR "Awareness" OR "Cognitive Dissonance" OR "Cognitive Reserve" OR "Comprehension" OR "Consciousness" OR "Imagination" OR "Dreams" OR "Fantasy" OR "Intuition" OR "Metacognition" OR "Cognitive Rumination" OR "cerebral function" OR "cerebral functions" OR "Mental Function" OR "Fatigue" OR "Muscle Fatigue" OR "Fatigue" OR "Fatigue*" OR "Quality of Life" OR "quality of life" OR "QoL" OR "life quality" OR "HRQoL" OR "PROM" OR "PROMs" OR "Patient Reported Outcome" OR "Patient Reported Outcome" OR "Patient Reported Outcomes")):ti,ab,kw NOT ("acute anemia" OR "trauma" OR "trauma*" OR "Injury" OR "major surgery" OR "major surgery" OR "gastro intestinal bleeding" OR "gastrointestinal bleeding" OR "Gastrointestinal Hemorrhage" OR "Gastrointestinal Hemorrhage" OR "Gastrointestinal Haemorrhage" OR "Gastro intestinal Hemorrhage" OR "Gastro intestinal Haemorrhage" OR "glycosylated" OR "Hemorrhagic Shock" OR "hemorrhagic shock" OR "Transgender" OR "transsexual*" OR "trans sexual*" OR "transgender*" OR "trans gender*" OR "acute bleeding" OR "acute hemorrhag*" OR "acute haemorrhag*"):ti

Sub-vraag 1:

(("chronic anaemia" OR "chronic anaemi*" OR "chronic anemia" OR "chronic anemi*" OR (("Anemia" OR "Anemia" OR "Anaemia" OR "Anemi*" OR "Anaemi*") AND ("Chronic Disease" OR "Chronic" OR "Chronic*")) OR (("Anemia" OR "Anemia" OR "Anaemia" OR "Anemi*" OR "Anaemi*") AND ("Hemodialysis" OR "Renal Dialysis" OR "Hemodialysis" OR "Haemodialysis")) OR (("Anemia" OR "Anemia" OR "Anaemia" OR "Anemi*" OR "Anaemi*") AND ("Myelodysplastic Syndrome" OR "Myelodysplastic Syndromes" OR "Myelodysplastic Syndromes" OR "MDS")) OR (("Anemia" OR "Anemia" OR "Anaemia" OR "Anemi*" OR "Anaemi*") AND ("myeloproliferative neoplasm" OR "myeloproliferative neoplasms" OR "myeloproliferative neoplasms" OR "myeloproliferative neoplasia" OR "MPN" OR "MPNs")) OR "Sickle Cell Anemia" OR "sickle cell anemia" OR "sickle cell anemi*" OR "sickle cell anaemia" OR "sickle cell anaemi*" OR "sickle cell disease" OR "Acute Chest Syndrome" OR "Haemoglobin SC Disease" OR "Hemoglobin SC Disease" OR "Sickle Cell Trait" OR "Thalassemia" OR "Thalassemia" OR "Thalassemi*" OR "Aplastic Anemia" OR "aplastic anemia" OR "aplastic anemi*" OR "aplastic anaemia" OR "aplastic anaemi*" OR "fanconi anemia" OR "fanconi anemi*" OR "fanconi anaemia" OR "fanconi anaemi*" OR "diamond blackfan anemia" OR "diamond blackfan anemi*" OR "diamond blackfan anaemia" OR "diamond blackfan anaemi*" OR "congenital hypoplastic anemia" OR "congenital hypoplastic anemi*" OR "congenital hypoplastic anaemia" OR "congenital hypoplastic anaemi*") AND ("Erythropoietin" OR "Erythropoietin" OR "Erythropoietin*" OR "EPO" OR "epoetin" OR "liberal transfusion strategy" OR "liberal transfusion" OR "higher nadir" OR "higher nadir*" OR "higher threshold" OR "higher threshold*" OR "iron therapy" OR "iron therap*" OR "iron treatment" OR "iron treat*" OR "Iron therapy" OR "altitude training" OR (("Altitude" OR "altitude" OR "altitude*") AND ("training" OR "Exercise" OR "Training" OR "exercise"))) AND ("VO2max" OR "VO2 max" OR "VO2max*" OR "VO2 max*" OR "maximal oxygen uptake" OR "maximal oxygen consumption" OR "Maximal Oxygen Uptake" OR "Heart Output" OR "Cardiac output" OR "stroke volume" OR "Heart Rate" OR "Heart rate" OR "Heart Rates" OR "Cardiac Rate" OR "Cardiac Rates" OR "Pulse Rate" OR "Pulse Rates" OR "Heartbeat" OR "Heartbeats" OR "Cardiac Chronotropy" OR "Cardiac Chronotropism" OR "Breathing Rate" OR "Respiratory rate" OR "Respiratory Rates" OR "Respiration Rate" OR "Respiration Rates" OR "physical capacity" OR "physical capacity" OR "Endurance" OR "Physical Endurance" OR "endurance" OR "stamina" OR "Anaerobic Threshold" OR "Exercise Tolerance" OR "Hand Strength" OR "Hand Strength" OR "grip strength" OR "pinch strength" OR "pinch strengths" OR "Hand Strengths" OR "Grip" OR "Grips" OR "Grasp" OR "Grasps" OR "muscle mass" OR "muscle mass" OR "Muscle Strength" OR "Muscle Strength" OR "Cognition" OR "Cognition" OR "Cognit*" OR "Awareness" OR "Cognitive Dissonance" OR "Cognitive Reserve" OR "Comprehension" OR "Consciousness" OR "Imagination" OR "Dreams" OR "Fantasy" OR "Intuition" OR "Metacognition" OR "Cognitive Rumination" OR "cerebral function" OR "cerebral functions" OR "Mental Function" OR "Fatigue" OR "Muscle Fatigue" OR "Fatigue" OR "Fatigue*" OR "Quality of Life" OR "quality of life" OR "QoL" OR "life quality" OR "HRQoL" OR "PROM" OR "PROMs" OR "Patient Reported Outcome" OR "Patient Reported Outcome" OR "Patient Reported Outcomes")):ti,ab,kw NOT ("acute anemia" OR "trauma" OR "trauma*" OR "Injury" OR "major surgery" OR "major surgery" OR "gastro intestinal bleeding" OR "gastrointestinal bleeding" OR "Gastrointestinal Hemorrhage" OR "Gastrointestinal Hemorrhage" OR "Gastrointestinal Haemorrhage" OR "Gastro intestinal Hemorrhage" OR "Gastro intestinal Haemorrhage" OR "glycosylated" OR "Hemorrhagic Shock" OR "hemorrhagic shock" OR "Transgender" OR "transsexual*" OR "trans sexual*" OR "transgender*" OR "trans gender*" OR "acute bleeding" OR "acute hemorrhag*" OR "acute haemorrhag*"):ti

Sub-vraag 2:

(("Normal Human" OR "Healthy Participant" OR "Healthy Participants" OR "Healthy Subject" OR "Healthy Subjects" OR "Healthy Volunteer" OR "Human Volunteer" OR "Human Volunteers" OR "Normal Volunteer" OR "Normal Volunteers" OR "Athlete" OR "Athletes" OR "Athlete" OR "fit patients" OR "fit patient") AND ("Erythropoietin" OR "Erythropoietin" OR "Erythropoietin*" OR "EPO" OR "epoetin" OR "liberal transfusion strategy" OR "liberal transfusion" OR "higher nadir" OR "higher nadir*" OR "higher threshold" OR "higher threshold*" OR "iron therapy" OR "iron therap*" OR "iron treatment" OR "iron treat*" OR "Iron therapy" OR "altitude training" OR (("Altitude" OR "altitude" OR "altitude*") AND ("training" OR "Exercise" OR "Training" OR "exercise"))) AND ("VO2max" OR "VO2 max" OR "VO2max*" OR "VO2 max*" OR "maximal oxygen uptake" OR "maximal oxygen consumption" OR "Maximal Oxygen Uptake" OR "Heart Output" OR "Cardiac output" OR "stroke volume" OR "Heart Rate" OR "Heart rate" OR "Heart Rates" OR "Cardiac Rate" OR "Cardiac Rates" OR "Pulse Rate" OR "Pulse Rates" OR "Heartbeat" OR "Heartbeats" OR "Cardiac Chronotropy" OR "Cardiac Chronotropism" OR "Breathing Rate" OR "Respiratory rate" OR "Respiratory Rates" OR "Respiration Rate" OR "Respiration Rates" OR "physical capacity" OR "physical capacity" OR "Endurance" OR "Physical Endurance" OR "endurance" OR "stamina" OR "Anaerobic Threshold" OR "Exercise Tolerance" OR "Hand Strength" OR "Hand Strength" OR "grip strength" OR "pinch strength" OR "pinch strengths" OR "Hand Strengths" OR "Grip" OR "Grips" OR "Grasp" OR "Grasps" OR "muscle mass" OR "muscle mass" OR "Muscle Strength" OR "Muscle Strength" OR "Cognition" OR "Cognition" OR "Cognit*" OR "Awareness" OR "Cognitive Dissonance" OR "Cognitive Reserve" OR "Comprehension" OR "Consciousness" OR "Imagination" OR "Dreams" OR "Fantasy" OR "Intuition" OR "Metacognition" OR "Cognitive Rumination" OR "cerebral function" OR "cerebral functions" OR "Mental Function" OR "Fatigue" OR "Muscle Fatigue" OR "Fatigue" OR "Fatigue*" OR "Quality of Life" OR "quality of life" OR "QoL" OR "life quality" OR "HRQoL" OR "PROM" OR "PROMs" OR "Patient Reported Outcome" OR "Patient Reported Outcome" OR "Patient Reported Outcomes")):ti,ab,kw NOT ("acute anemia" OR "trauma" OR "trauma*" OR "Injury" OR "major surgery" OR "major surgery" OR "gastro intestinal bleeding" OR "gastrointestinal bleeding" OR "Gastrointestinal Hemorrhage" OR "Gastrointestinal Hemorrhage" OR "Gastrointestinal Haemorrhage" OR "Gastro intestinal Hemorrhage" OR "Gastro intestinal Haemorrhage" OR "glycosylated" OR "Hemorrhagic Shock" OR "hemorrhagic shock" OR "Transgender" OR "transsexual*" OR "trans sexual*" OR "transgender*" OR "trans gender*" OR "acute bleeding" OR "acute hemorrhag*" OR "acute haemorrhag*"):ti

Sub-vraag 3:

(("Erythrocythemia" OR "Erythrocythem*" OR "Erythrocythaemia" OR "Erythrocythaem*" OR "Erythrocytemia" OR "Erythrocytem*" OR "Erythrocytaemia" OR "Erythrocytaem*" OR "Polycythemia Vera" OR "Polycythemia" OR "Polycythemia" OR "Polycythemi*" OR "Polycythaemia" OR "Polycythaemi*" OR "erythrocytosis" OR "erytrocytosis") AND ("Phlebotomy" OR "Phlebotomy" OR "Phlebotom*" OR "Venesection*" OR "Venipuncture*" OR "Hydroxyurea" OR "Hydroxyurea" OR "Hydroxyurea*" OR "hydrea" OR "Hydroxycarbamid*" OR "Oncocarbide*" OR "Busulfan" OR "Busulfan" OR "Busulfan*" OR "Busulphan" OR "Busulfex" OR "Myleran" OR "Myelosan" OR "Mylecytan" OR "Glyzophrol" OR "Interferon" OR "Interferons" OR "Interferon" OR "Interferon*") AND ("VO2max" OR "VO2 max" OR "VO2max*" OR "VO2 max*" OR "maximal oxygen uptake" OR "maximal oxygen consumption" OR "Maximal Oxygen Uptake" OR "Heart Output" OR "Cardiac output" OR "stroke volume" OR "Heart Rate" OR "Heart rate" OR "Heart Rates" OR "Cardiac Rate" OR "Cardiac Rates" OR "Pulse Rate" OR "Pulse Rates" OR "Heartbeat" OR "Heartbeats" OR "Cardiac Chronotropy" OR "Cardiac Chronotropism" OR "Breathing Rate" OR "Respiratory rate" OR "Respiratory Rates" OR "Respiration Rate" OR "Respiration Rates" OR "physical capacity" OR "physical capacity" OR "Endurance" OR "Physical Endurance" OR "endurance" OR "stamina" OR "Anaerobic Threshold" OR "Exercise Tolerance" OR "Hand Strength" OR "Hand Strength" OR "grip strength" OR "pinch strength" OR "pinch strengths" OR "Hand Strengths" OR "Grip" OR "Grips" OR "Grasp" OR "Grasps" OR "muscle mass" OR "muscle mass" OR "Muscle Strength" OR "Muscle Strength" OR "Cognition" OR "Cognition" OR "Cognit*" OR "Awareness" OR "Cognitive Dissonance" OR "Cognitive Reserve" OR "Comprehension" OR "Consciousness" OR "Imagination" OR "Dreams" OR "Fantasy" OR "Intuition" OR "Metacognition" OR "Cognitive Rumination" OR "cerebral function" OR "cerebral functions" OR "Mental Function" OR "Fatigue" OR "Muscle Fatigue" OR "Fatigue" OR "Fatigue*" OR "Quality of Life" OR "quality of life" OR "QoL" OR "life quality" OR "HRQoL" OR "PROM" OR "PROMs" OR "Patient Reported Outcome" OR "Patient Reported Outcome" OR "Patient Reported Outcomes")):ti,ab,kw NOT ("acute anemia" OR "trauma" OR "trauma*" OR "Injury" OR "major surgery" OR "major surgery" OR "gastro intestinal bleeding" OR "gastrointestinal bleeding" OR "Gastrointestinal Hemorrhage" OR "Gastrointestinal Hemorrhage" OR "Gastrointestinal Haemorrhage" OR "Gastro intestinal Hemorrhage" OR "Gastro intestinal Haemorrhage" OR "glycosylated" OR "Hemorrhagic Shock" OR "hemorrhagic shock" OR "Transgender" OR "transsexual*" OR "trans sexual*" OR "transgender*" OR "trans gender*" OR "acute bleeding" OR "acute hemorrhag*" OR "acute haemorrhag*"):ti

NOT DT=(meeting abstract)

**Emcare** <http://ovidsp.ovid.com/ovidweb.cgi?T=JS&NEWS=n&CSC=Y&PAGE=main&D=emcr>

Basisvraag:

- met nabijheidsoperator Adjacency (zonder de mesh exp "Hemoglobin"/)

(("Hemoglobins".ti,ab OR "Haemoglobins".ti,ab OR "Hemoglobin".ti,ab OR "Haemoglobin".ti,ab OR "Hemoglobin*".ti,ab OR "Haemoglobin*".ti,ab OR "alpha-Globin".ti,ab OR "alpha-Globin*".ti,ab OR "alpha-Globins".ti,ab OR "beta-Globin".ti,ab OR "beta-Globin*".ti,ab OR "beta-Globins".ti,ab OR "Carboxyhemoglobin".ti,ab OR "Carboxyhemoglobin*".ti,ab OR "Carboxyhemoglobins".ti,ab OR "delta-Globin".ti,ab OR "delta-Globin*".ti,ab OR "delta-Globins".ti,ab OR "epsilon-Globin".ti,ab OR "epsilon-Globin*".ti,ab OR "epsilon-Globins".ti,ab OR "Erythrocruorin".ti,ab OR "Erythrocruorin*".ti,ab OR "Erythrocruorins".ti,ab OR "gamma-Globin".ti,ab OR "gamma-Globin*".ti,ab OR "gamma-Globins".ti,ab OR "Methemoglobin".ti,ab OR "Methemoglobin*".ti,ab OR "Methemoglobins".ti,ab OR "Oxyhemoglobin".ti,ab OR "Oxyhemoglobin*".ti,ab OR "Oxyhemoglobins".ti,ab OR "Sulfhemoglobin".ti,ab OR "Sulfhemoglobin*".ti,ab OR "Sulfhemoglobins".ti,ab OR "zeta-Globin".ti,ab OR "zeta-Globin*".ti,ab OR "zeta-Globins".ti,ab) **ADJ4** ("mass".ti,ab OR "concentration".ti,ab OR "concentrations".ti,ab OR "higher hemoglobin*".ti,ab OR "higher haemoglobin*".ti,ab OR "lower hemoglobin*".ti,ab OR "lower haemoglobin*".ti,ab OR "hemoglobin increas*".ti,ab OR "haemoglobin increas*".ti,ab OR "hemoglobin decreas*".ti,ab OR "haemoglobin decreas*".ti,ab OR "hemoglobin chang*".ti,ab OR "haemoglobin chang*".ti,ab OR "hemoglobin volume".ti,ab OR "haemoglobin volume".ti,ab) AND ("VO2max".ti,ab OR "VO2 max".ti,ab OR "VO2max*".ti,ab OR "VO2 max*".ti,ab OR "maximal oxygen uptake".ti,ab OR "maximal oxygen consumption".ti,ab OR exp *"Maximal Oxygen Uptake"/ OR exp *"Heart Output"/ OR "Cardiac output".ti,ab OR "stroke volume".ti,ab OR exp *"Heart Rate"/ OR "Heart rate".ti,ab OR "Heart Rates".ti,ab OR "Cardiac Rate".ti,ab OR "Cardiac Rates".ti,ab OR "Pulse Rate".ti,ab OR "Pulse Rates".ti,ab OR "Heartbeat".ti,ab OR "Heartbeats".ti,ab OR "Cardiac Chronotropy".ti,ab OR "Cardiac Chronotropism".ti,ab OR exp *"Breathing Rate"/ OR "Respiratory rate".ti,ab OR "Respiratory Rates".ti,ab OR "Respiration Rate".ti,ab OR "Respiration Rates".ti,ab OR exp *"physical capacity"/ OR "physical capacity".ti,ab OR exp *"Endurance"/ OR "Physical Endurance".ti,ab OR "endurance".ti,ab OR "stamina".ti,ab OR "Anaerobic Threshold".ti,ab OR "Exercise Tolerance".ti,ab OR exp *"Hand Strength"/ OR "Hand Strength".ti,ab OR "grip strength".ti,ab OR "pinch strength".ti,ab OR "pinch strengths".ti,ab OR "Hand Strengths".ti,ab OR "Grip".ti,ab OR "Grips".ti,ab OR "Grasp".ti,ab OR "Grasps".ti,ab OR exp *"muscle mass"/ OR "muscle mass".ti,ab OR exp *"Muscle Strength"/ OR "Muscle Strength".ti,ab OR exp *"Cognition"/ OR "Cognition".ti,ab OR "Cognit*".ti,ab OR "Awareness".ti,ab OR "Cognitive Dissonance".ti,ab OR "Cognitive Reserve".ti,ab OR "Comprehension".ti,ab OR "Consciousness".ti,ab OR "Imagination".ti,ab OR "Dreams".ti,ab OR "Fantasy".ti,ab OR "Intuition".ti,ab OR "Metacognition".ti,ab OR "Cognitive Rumination".ti,ab OR "cerebral function".ti,ab OR "cerebral functions".ti,ab OR *"Mental Function"/ OR exp *"Fatigue"/ OR exp *"Muscle Fatigue"/ OR "Fatigue".ti,ab OR "Fatigue*".ti,ab OR exp *"Quality of Life"/ OR "quality of life".ti,ab OR "QoL".ti,ab OR "life quality".ti,ab OR "HRQoL".ti,ab OR "PROM".ti,ab OR "PROMs".ti,ab OR *"Patient Reported-Outcome"/ OR "Patient Reported Outcome".ti,ab OR "Patient Reported Outcomes".ti,ab) AND **(exp** "**review"/ OR "review".mp OR "overview".mp OR "review*".mp OR "overview*".mp)** NOT ("acute anemia".ti OR "trauma".ti OR "trauma*".ti OR exp *"Injury"/ OR *"major surgery"/ OR "major surgery".ti OR "gastro-intestinal bleeding".ti OR "gastrointestinal bleeding".ti OR exp *"Gastrointestinal Hemorrhage"/ OR "Gastrointestinal Hemorrhage".ti OR "Gastrointestinal Haemorrhage".ti OR "Gastro intestinal Hemorrhage".ti OR "Gastro intestinal Haemorrhage".ti OR "glycosylated".ti OR exp *"Hemorrhagic Shock"/ OR "hemorrhagic shock".ti OR exp *"Transgender"/ OR "transsexual*".ti OR "trans sexual*".ti OR "transgender*".ti OR "trans gender*".ti OR "acute bleeding".ti OR "acute hemorrhag*".ti OR "acute haemorrhag*".ti) NOT (conference review or conference abstract).pt)

Sub-vraag 1:

(("chronic anaemia".ti,ab OR "chronic anaemi*".ti,ab OR "chronic anemia".ti,ab OR "chronic anemi*".ti,ab OR ((exp *"Anemia"/ OR "Anemia".ti,ab OR "Anaemia".ti,ab OR "Anemi*".ti,ab OR "Anaemi*".ti,ab) AND (exp *"Chronic Disease"/ OR "Chronic".ti,ab OR "Chronic*".ti,ab)) OR ((exp *"Anemia"/ OR "Anemia".ti,ab OR "Anaemia".ti,ab OR "Anemi*".ti,ab OR "Anaemi*".ti,ab) AND (exp *"Hemodialysis"/ OR "Renal Dialysis".ti,ab OR "Hemodialysis".ti,ab OR "Haemodialysis".ti,ab)) OR ((exp *"Anemia"/ OR "Anemia".ti,ab OR "Anaemia".ti,ab OR "Anemi*".ti,ab OR "Anaemi*".ti,ab) AND (exp *"Myelodysplastic Syndrome"/ OR "Myelodysplastic Syndromes".ti,ab OR "Myelodysplastic Syndromes".ti,ab OR "MDS".ti,ab)) OR ((exp *"Anemia"/ OR "Anemia".ti,ab OR "Anaemia".ti,ab OR "Anemi*".ti,ab OR "Anaemi*".ti,ab) AND (exp *"myeloproliferative neoplasm"/ OR "myeloproliferative neoplasms".ti,ab OR "myeloproliferative neoplasms".ti,ab OR "myeloproliferative neoplasia".ti,ab OR "MPN".ti,ab OR "MPNs".ti,ab)) OR exp *"Sickle Cell Anemia"/ OR "sickle cell anemia".ti,ab OR "sickle cell anemi*".ti,ab OR "sickle cell anaemia".ti,ab OR "sickle cell anaemi*".ti,ab OR "sickle cell disease".ti,ab OR "Acute Chest Syndrome".ti,ab OR "Haemoglobin SC Disease".ti,ab OR "Hemoglobin SC Disease".ti,ab OR "Sickle Cell Trait".ti,ab OR exp *"Thalassemia"/ OR "Thalassemia".ti,ab OR "Thalassemi*".ti,ab OR exp *"Aplastic Anemia"/ OR "aplastic anemia".ti,ab OR "aplastic anemi*".ti,ab OR "aplastic anaemia".ti,ab OR "aplastic anaemi*".ti,ab OR "fanconi anemia".ti,ab OR "fanconi anemi*".ti,ab OR "fanconi anaemia".ti,ab OR "fanconi anaemi*".ti,ab OR "diamond-blackfan anemia".ti,ab OR "diamond-blackfan anemi*".ti,ab OR "diamond-blackfan anaemia".ti,ab OR "diamond-blackfan anaemi*".ti,ab OR "congenital hypoplastic anemia".ti,ab OR "congenital hypoplastic anemi*".ti,ab OR "congenital hypoplastic anaemia".ti,ab OR "congenital hypoplastic anaemi*".ti,ab) AND (exp *"Erythropoietin"/ OR "Erythropoietin".ti,ab OR "Erythropoietin*".ti,ab OR "EPO".ti,ab OR "epoetin".ti,ab OR "liberal transfusion strategy".ti,ab OR "liberal transfusion".ti,ab OR "higher nadir".ti,ab OR "higher nadir*".ti,ab OR "higher threshold".ti,ab OR "higher threshold*".ti,ab OR "iron therapy".ti,ab OR "iron therap*".ti,ab OR "iron treatment".ti,ab OR "iron treat*".ti,ab OR exp *"Iron therapy"/ OR "altitude training".ti,ab OR ((exp *"Altitude"/ OR "altitude".ti,ab OR "altitude*".ti,ab) AND ("training".ti,ab OR exp *"Exercise"/ OR exp *"Training"/ OR "exercise".ti,ab))) AND ("VO2max".ti,ab OR "VO2 max".ti,ab OR "VO2max*".ti,ab OR "VO2 max*".ti,ab OR "maximal oxygen uptake".ti,ab OR "maximal oxygen consumption".ti,ab OR exp *"Maximal Oxygen Uptake"/ OR exp *"Heart Output"/ OR "Cardiac output".ti,ab OR "stroke volume".ti,ab OR exp *"Heart Rate"/ OR "Heart rate".ti,ab OR "Heart Rates".ti,ab OR "Cardiac Rate".ti,ab OR "Cardiac Rates".ti,ab OR "Pulse Rate".ti,ab OR "Pulse Rates".ti,ab OR "Heartbeat".ti,ab OR "Heartbeats".ti,ab OR "Cardiac Chronotropy".ti,ab OR "Cardiac Chronotropism".ti,ab OR exp *"Breathing Rate"/ OR "Respiratory rate".ti,ab OR "Respiratory Rates".ti,ab OR "Respiration Rate".ti,ab OR "Respiration Rates".ti,ab OR exp *"physical capacity"/ OR "physical capacity".ti,ab OR exp *"Endurance"/ OR "Physical Endurance".ti,ab OR "endurance".ti,ab OR "stamina".ti,ab OR "Anaerobic Threshold".ti,ab OR "Exercise Tolerance".ti,ab OR exp *"Hand Strength"/ OR "Hand Strength".ti,ab OR "grip strength".ti,ab OR "pinch strength".ti,ab OR "pinch strengths".ti,ab OR "Hand Strengths".ti,ab OR "Grip".ti,ab OR "Grips".ti,ab OR "Grasp".ti,ab OR "Grasps".ti,ab OR exp *"muscle mass"/ OR "muscle mass".ti,ab OR exp *"Muscle Strength"/ OR "Muscle Strength".ti,ab OR exp *"Cognition"/ OR "Cognition".ti,ab OR "Cognit*".ti,ab OR "Awareness".ti,ab OR "Cognitive Dissonance".ti,ab OR "Cognitive Reserve".ti,ab OR "Comprehension".ti,ab OR "Consciousness".ti,ab OR "Imagination".ti,ab OR "Dreams".ti,ab OR "Fantasy".ti,ab OR "Intuition".ti,ab OR "Metacognition".ti,ab OR "Cognitive Rumination".ti,ab OR "cerebral function".ti,ab OR "cerebral functions".ti,ab OR *"Mental Function"/ OR exp *"Fatigue"/ OR exp *"Muscle Fatigue"/ OR "Fatigue".ti,ab OR "Fatigue*".ti,ab OR exp *"Quality of Life"/ OR "quality of life".ti,ab OR "QoL".ti,ab OR "life quality".ti,ab OR "HRQoL".ti,ab OR "PROM".ti,ab OR "PROMs".ti,ab OR *"Patient Reported-Outcome"/ OR "Patient Reported Outcome".ti,ab OR "Patient Reported Outcomes".ti,ab) AND **(exp** "**review"/ OR "review".mp OR "overview".mp OR "review*".mp OR "overview*".mp)** NOT ("acute anemia".ti OR "trauma".ti OR "trauma*".ti OR exp *"Injury"/ OR *"major surgery"/ OR "major surgery".ti OR "gastro-intestinal bleeding".ti OR "gastrointestinal bleeding".ti OR exp *"Gastrointestinal Hemorrhage"/ OR "Gastrointestinal Hemorrhage".ti OR "Gastrointestinal Haemorrhage".ti OR "Gastro intestinal Hemorrhage".ti OR "Gastro intestinal Haemorrhage".ti OR "glycosylated".ti OR exp *"Hemorrhagic Shock"/ OR "hemorrhagic shock".ti OR exp *"Transgender"/ OR "transsexual*".ti OR "trans sexual*".ti OR "transgender*".ti OR "trans gender*".ti OR "acute bleeding".ti OR "acute hemorrhag*".ti OR "acute haemorrhag*".ti) NOT (conference review or conference abstract).pt)

Sub-vraag 2:

((*"Normal Human"/ OR "Healthy Participant".ti,ab OR "Healthy Participants".ti,ab OR "Healthy Subject".ti,ab OR "Healthy Subjects".ti,ab OR "Healthy Volunteer".ti,ab OR "Human Volunteer".ti,ab OR "Human Volunteers".ti,ab OR "Normal Volunteer".ti,ab OR "Normal Volunteers".ti,ab OR exp *"Athlete"/ OR "Athletes".ti,ab OR "Athlete".ti,ab OR "fit patients".ti,ab OR "fit patient".ti,ab) AND (exp *"Erythropoietin"/ OR "Erythropoietin".ti,ab OR "Erythropoietin*".ti,ab OR "EPO".ti,ab OR "epoetin".ti,ab OR "liberal transfusion strategy".ti,ab OR "liberal transfusion".ti,ab OR "higher nadir".ti,ab OR "higher nadir*".ti,ab OR "higher threshold".ti,ab OR "higher threshold*".ti,ab OR "iron therapy".ti,ab OR "iron therap*".ti,ab OR "iron treatment".ti,ab OR "iron treat*".ti,ab OR exp *"Iron therapy"/ OR "altitude training".ti,ab OR ((exp *"Altitude"/ OR "altitude".ti,ab OR "altitude*".ti,ab) AND ("training".ti,ab OR exp *"Exercise"/ OR exp *"Training"/ OR "exercise".ti,ab))) AND ("VO2max".ti,ab OR "VO2 max".ti,ab OR "VO2max*".ti,ab OR "VO2 max*".ti,ab OR "maximal oxygen uptake".ti,ab OR "maximal oxygen consumption".ti,ab OR exp *"Maximal Oxygen Uptake"/ OR exp *"Heart Output"/ OR "Cardiac output".ti,ab OR "stroke volume".ti,ab OR exp *"Heart Rate"/ OR "Heart rate".ti,ab OR "Heart Rates".ti,ab OR "Cardiac Rate".ti,ab OR "Cardiac Rates".ti,ab OR "Pulse Rate".ti,ab OR "Pulse Rates".ti,ab OR "Heartbeat".ti,ab OR "Heartbeats".ti,ab OR "Cardiac Chronotropy".ti,ab OR "Cardiac Chronotropism".ti,ab OR exp *"Breathing Rate"/ OR "Respiratory rate".ti,ab OR "Respiratory Rates".ti,ab OR "Respiration Rate".ti,ab OR "Respiration Rates".ti,ab OR exp *"physical capacity"/ OR "physical capacity".ti,ab OR exp *"Endurance"/ OR "Physical Endurance".ti,ab OR "endurance".ti,ab OR "stamina".ti,ab OR "Anaerobic Threshold".ti,ab OR "Exercise Tolerance".ti,ab OR exp *"Hand Strength"/ OR "Hand Strength".ti,ab OR "grip strength".ti,ab OR "pinch strength".ti,ab OR "pinch strengths".ti,ab OR "Hand Strengths".ti,ab OR "Grip".ti,ab OR "Grips".ti,ab OR "Grasp".ti,ab OR "Grasps".ti,ab OR exp *"muscle mass"/ OR "muscle mass".ti,ab OR exp *"Muscle Strength"/ OR "Muscle Strength".ti,ab OR exp *"Cognition"/ OR "Cognition".ti,ab OR "Cognit*".ti,ab OR "Awareness".ti,ab OR "Cognitive Dissonance".ti,ab OR "Cognitive Reserve".ti,ab OR "Comprehension".ti,ab OR "Consciousness".ti,ab OR "Imagination".ti,ab OR "Dreams".ti,ab OR "Fantasy".ti,ab OR "Intuition".ti,ab OR "Metacognition".ti,ab OR "Cognitive Rumination".ti,ab OR "cerebral function".ti,ab OR "cerebral functions".ti,ab OR *"Mental Function"/ OR exp *"Fatigue"/ OR exp *"Muscle Fatigue"/ OR "Fatigue".ti,ab OR "Fatigue*".ti,ab OR exp *"Quality of Life"/ OR "quality of life".ti,ab OR "QoL".ti,ab OR "life quality".ti,ab OR "HRQoL".ti,ab OR "PROM".ti,ab OR "PROMs".ti,ab OR *"Patient Reported-Outcome"/ OR "Patient Reported Outcome".ti,ab OR "Patient Reported Outcomes".ti,ab) AND **(exp** "**review"/ OR "review".mp OR "overview".mp OR "review*".mp OR "overview*".mp)** NOT ("acute anemia".ti OR "trauma".ti OR "trauma*".ti OR exp *"Injury"/ OR *"major surgery"/ OR "major surgery".ti OR "gastro-intestinal bleeding".ti OR "gastrointestinal bleeding".ti OR exp *"Gastrointestinal Hemorrhage"/ OR "Gastrointestinal Hemorrhage".ti OR "Gastrointestinal Haemorrhage".ti OR "Gastro intestinal Hemorrhage".ti OR "Gastro intestinal Haemorrhage".ti OR "glycosylated".ti OR exp *"Hemorrhagic Shock"/ OR "hemorrhagic shock".ti OR exp *"Transgender"/ OR "transsexual*".ti OR "trans sexual*".ti OR "transgender*".ti OR "trans gender*".ti OR "acute bleeding".ti OR "acute hemorrhag*".ti OR "acute haemorrhag*".ti) NOT (conference review or conference abstract).pt)

Sub-vraag 3:

(("Erythrocythemia".ti,ab OR "Erythrocythem*".ti,ab OR "Erythrocythaemia".ti,ab OR "Erythrocythaem*".ti,ab OR "Erythrocytemia".ti,ab OR "Erythrocytem*".ti,ab OR "Erythrocytaemia".ti,ab OR "Erythrocytaem*".ti,ab OR "Polycythemia Vera"/ OR exp *"Polycythemia"/ OR "Polycythemia".ti,ab OR "Polycythemi*".ti,ab OR "Polycythaemia".ti,ab OR "Polycythaemi*".ti,ab OR "erythrocytosis".ti,ab OR "erytrocytosis".ti,ab) AND (exp *"Phlebotomy"/ OR "Phlebotomy".ti,ab OR "Phlebotom*".ti,ab OR "Venesection*".ti,ab OR "Venipuncture*".ti,ab OR exp *"Hydroxyurea"/ OR "Hydroxyurea".ti,ab OR "Hydroxyurea*".ti,ab OR "hydrea".ti,ab OR "Hydroxycarbamid*".ti,ab OR "Oncocarbide*".ti,ab OR exp *"Busulfan"/ OR "Busulfan".ti,ab OR "Busulfan*".ti,ab OR "Busulphan".ti,ab OR "Busulfex".ti,ab OR "Myleran".ti,ab OR "Myelosan".ti,ab OR "Mylecytan".ti,ab OR "Glyzophrol".ti,ab OR exp *"Interferon"/ OR "Interferons".ti,ab OR "Interferon".ti,ab OR "Interferon*".ti,ab) AND ("VO2max".ti,ab OR "VO2 max".ti,ab OR "VO2max*".ti,ab OR "VO2 max*".ti,ab OR "maximal oxygen uptake".ti,ab OR "maximal oxygen consumption".ti,ab OR exp *"Maximal Oxygen Uptake"/ OR exp *"Heart Output"/ OR "Cardiac output".ti,ab OR "stroke volume".ti,ab OR exp *"Heart Rate"/ OR "Heart rate".ti,ab OR "Heart Rates".ti,ab OR "Cardiac Rate".ti,ab OR "Cardiac Rates".ti,ab OR "Pulse Rate".ti,ab OR "Pulse Rates".ti,ab OR "Heartbeat".ti,ab OR "Heartbeats".ti,ab OR "Cardiac Chronotropy".ti,ab OR "Cardiac Chronotropism".ti,ab OR exp *"Breathing Rate"/ OR "Respiratory rate".ti,ab OR "Respiratory Rates".ti,ab OR "Respiration Rate".ti,ab OR "Respiration Rates".ti,ab OR exp *"physical capacity"/ OR "physical capacity".ti,ab OR exp *"Endurance"/ OR "Physical Endurance".ti,ab OR "endurance".ti,ab OR "stamina".ti,ab OR "Anaerobic Threshold".ti,ab OR "Exercise Tolerance".ti,ab OR exp *"Hand Strength"/ OR "Hand Strength".ti,ab OR "grip strength".ti,ab OR "pinch strength".ti,ab OR "pinch strengths".ti,ab OR "Hand Strengths".ti,ab OR "Grip".ti,ab OR "Grips".ti,ab OR "Grasp".ti,ab OR "Grasps".ti,ab OR exp *"muscle mass"/ OR "muscle mass".ti,ab OR exp *"Muscle Strength"/ OR "Muscle Strength".ti,ab OR exp *"Cognition"/ OR "Cognition".ti,ab OR "Cognit*".ti,ab OR "Awareness".ti,ab OR "Cognitive Dissonance".ti,ab OR "Cognitive Reserve".ti,ab OR "Comprehension".ti,ab OR "Consciousness".ti,ab OR "Imagination".ti,ab OR "Dreams".ti,ab OR "Fantasy".ti,ab OR "Intuition".ti,ab OR "Metacognition".ti,ab OR "Cognitive Rumination".ti,ab OR "cerebral function".ti,ab OR "cerebral functions".ti,ab OR *"Mental Function"/ OR exp *"Fatigue"/ OR exp *"Muscle Fatigue"/ OR "Fatigue".ti,ab OR "Fatigue*".ti,ab OR exp *"Quality of Life"/ OR "quality of life".ti,ab OR "QoL".ti,ab OR "life quality".ti,ab OR "HRQoL".ti,ab OR "PROM".ti,ab OR "PROMs".ti,ab OR *"Patient Reported-Outcome"/ OR "Patient Reported Outcome".ti,ab OR "Patient Reported Outcomes".ti,ab) AND **(exp** "**review"/ OR "review".mp OR "overview".mp OR "review*".mp OR "overview*".mp)** NOT ("acute anemia".ti OR "trauma".ti OR "trauma*".ti OR exp *"Injury"/ OR *"major surgery"/ OR "major surgery".ti OR "gastro-intestinal bleeding".ti OR "gastrointestinal bleeding".ti OR exp *"Gastrointestinal Hemorrhage"/ OR "Gastrointestinal Hemorrhage".ti OR "Gastrointestinal Haemorrhage".ti OR "Gastro intestinal Hemorrhage".ti OR "Gastro intestinal Haemorrhage".ti OR "glycosylated".ti OR exp *"Hemorrhagic Shock"/ OR "hemorrhagic shock".ti OR exp *"Transgender"/ OR "transsexual*".ti OR "trans sexual*".ti OR "transgender*".ti OR "trans gender*".ti OR "acute bleeding".ti OR "acute hemorrhag*".ti OR "acute haemorrhag*".ti) NOT (conference review or conference abstract).pt)

###

# **Supplementary material 9:** PRIO tool

| Section/Topic | **(Sub-) item #** | **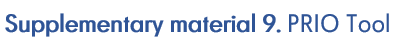Checklist item** | | | | | **Reported**  **on page #** |
| --- | --- | --- | --- | --- | --- | --- | --- |
| **TITLE** | | | | | | |  |
| 1. Title | 1a | Specify the study design with terms such as “overview of (systematic) reviews,” “umbrella review,” “(systematic) review of systematic reviews,” or “(systematic) meta-review” in the title of the OoSRs. | | | | | 1 |
|  | 1b | Mention “safety” or harms related terms, or the adverse event(s) of interest in the title of the OoSRs. | | | | | 1 |
| **ABSTRACT** | | | | | | |  |
| 2. Structured-like summary | 2a | Provide a structured-like abstract, as applicable: background, objective, data sources, selection criteria, data extraction, review appraisal, data synthesis methods, results, limitations, conclusions. | | | | | 2 |
|  | 2b | Report the main findings of analysis of harms undertaken in the OoSRs or/and in the included SRs. | | | | | 2 |
| **INTRODUCTION** | | | | | | |  |
| 3. Rationale | 3a | Specify the rationale and the scope (wide or narrow agendas) for the overview in the context of an existing body of knowledge on the topic. | | | | | 4 |
|  | 3b | Provide a balanced presentation of potential benefits and harms of the intervention(s). | | | | | 5 |
|  | 3c**^a^** | Define which events are considered harms according to previous literature and provide a clear rationale for the specific harms included in the OoSRs. | | | | | Not applicable |
| 4. Objectives  (PICOS) | 4 | Provide an explicit statement of research question(s) that specifies PICOS: | | | | | 5 |
|  |  | \|  \| \| --- \|  - Participants | \|  \| \| --- \|  - Interventions | \|  \| \| --- \|  - Comparators | \|  \| \| --- \|  - Outcomes | \|  \| \| --- \|  - Study design |  |
| **METHODS** | | | | | | |  |
| 5. Protocol and registration | 5a | Indicate clearly if a protocol exists or not. | | | | | 5 |
|  | 5b | If registered, provide the name of the registry (such as a valid Web address, PROSPERO). | | | | | 5 |
| 6. Eligibility criteria  & outcomes of interest | 6a | Specify inclusion and exclusion criteria for study design, participants, interventions and comparators in detail. | | | | | 5 |
|  | 6b | List (and define whenever it is necessary) the outcomes for which data were recorded, ideally include prioritization of main and additional outcomes. | | | | | 6 |
|  | 6c | Include adverse events as (primary or secondary) outcome of interest. Define them and grade their severity (such as mild, moderate, severe, fatal; severity could also be described in the appendix), if appropriate. | | | | | Not applicable |
|  | 6d**^b^** | Specify report characteristics (such as language restrictions, publication status, and years considered) used as criteria for eligibility for the OoSRs (see also item 7). | | | | | 6 |
| 7. Information sources | 7a | Search at least two electronic bases. | | | | | 5 |
|  | 7b | Search supplementary sources (e.g. hand-searching, reference lists, related reviews and guidelines, protocol registries, conference abstracts, and other gray literature). | | | | | 6 |
|  | 7c | Report the date last searched and/or dates of coverage for each database. | | | | | 5 |
| 8. Search strategy**^c^** | 8a | Specify full electronic search strategy (algorithm) for at least one database including any limits used (e.g. language and date restrictions-see also subitems 6d and 7c) such that it could be repeated. | | | | | Supp. 7 |
|  | 8b | Present any additional search process (e.g. algorithm or filter for adverse events, searches in pertinent websites) specifically to identify adverse events that have been investigated. | | | | | - |
| 9. Data management &  selection process | 9a**^d^** | Describe the software that was used to manage records and data throughout the OoSRs. | | | | | 6 |
|  | 9b | Define what is a SR and provide the process for selecting SRs and its relevant details (screening the title and abstract or full text by at least two reviewers, selection by multiple independent investigators and resolving disagreements by consensus). | | | | | 6 |
|  | 9c | Report any attempt to handle overlapping (include one review among multiple potential candidates by choosing for example the most updated SR, the most methodologically rigorous SR or the SR with larger number of primary studies). | | | | | 6 |
| 10. Additional search for  primary studies | 10 | Report additional search to identify eligible primary studies (e.g. searching in more databases or update the search) and its relevant details. | | | | | - |
| 11. Data collection process | 11a | Describe the method of data extraction from included SRs (e.g. data collection form, extraction in duplicate and independently, resolving disagreements by consensus). | | | | | 6 |
|  | 11b | Report any processes for obtaining, confirming or updating data from investigators (e.g. contact with authors of included reviews, obtain data from primary studies of included reviews). | | | | | 8 |
| 12. Data items | 12 | List (and define whenever is necessary) the specific variables for which data were recorded (e.g. PICOS items, number of included studies and participants, dose, length of follow up, results, funding sources) and any data assumptions and simplifications made. | | | | | 6 |
| 13. Assessment of methodological quality & quality of evidence | 13a | State the evaluation of reporting or/and methodological quality (eg. using PRISMA or PRISMA-harms, AMSTAR or R-AMSTAR) of the included reviews. | | | | | 6 |
|  | 13b**^e^** | State the evaluation of quality for individual studies that were included in the SRs (inform whether tools such as Jadad or RoB of Cochrane were used by the included reviews) and for the additional primary studies. | | | | | 6 |
|  | 13c | State the evaluation of quality of evidence (e.g. using GRADE approach). | | | | | 6 |
|  | 13d | Describe the methods (e.g. piloted forms, independently, in duplicate) used for the quality assessment. | | | | | 6 |
| 14. Meta-bias(es) | 14 | Specify any planned assessment of meta-bias(es) (such as publication bias or selective reporting across studies, ROBIS tool). | | | | | 6 |
| 15. Data synthesis | 15a | Specify clearly the method (narrative, meta-analysis or network meta-analysis) of handling or synthesizing data and their details (e.g. state the principal summary measures that were extracted or calculated, how heterogeneity was assessed, what statistical approaches were used if a quantitative synthesis has been conducted). | | | | | 6 |
|  | 15b | Describe the software that was used to analyze the data if a quantitative synthesis has been conducted. | | | | | 6 |
|  | 15c | Report if zero events are included in the studies and how they were handled in statistical analyses, if relevant. | | | | | - |
|  | 15d | Describe methods of any pre-specified additional analyses (such as sensitivity or subgroup analyses, meta-regression). | | | | | 6 |
| **RESULTS** | | | | | | |  |
| 16. Review & primary study selection | 16a | Provide the details of review selection (e.g. numbers of reviews screened, retrieved, and included and excluded in the overview) and the number of the additional eligible primary studies that were included, ideally with a flow diagram of the overview process. | | | | | 6 |
|  | 16b | Present a flow diagram that gives separately the number of studies focused on harms outcomes. | | | | | Fig. 2 |
|  | 16c**^c^** | List the studies (full citation) that were excluded after reading the full text and provide reasons. | | | | | Supp. 2 |
| 17. Review & primary study characteristics | 17a**^c^** | Describe characteristics of each included SR in tables (such as title or author, search date, PICOS, design and number of studies included, number and age range of participants, dose/frequency, follow up period [treatment duration], review limitations, results or conclusion) and of each additional primary study. | | | | | Supp. 1 |
|  | 17b | For each included SR report language and publication status restrictions that have been used. | | | | | Supp. 1 |
| 18. Overlapping | 18 | Present or/and discuss about overlapping of studies within SRs (at least one of the following): | | | | | 8 |
|  |  | - Present measures of overlap (such as CCA). | | | | |  |
|  |  | - Provide citation matrix.**^c^** | | | | |  |
|  |  | - Give the number of index publications or/and discuss about overlapping.**^f^** | | | | | 8 |
| 19. Present assessment of methodological quality & quality of evidence | 19 | Present results in text or/and tables**^c^** of any quality assessment (see also subitems 13a-c): | | | | | Fig 3 |
|  |  | - Reporting or/and methodological quality of the included SRs. | | | | | Fig 2 |
|  |  | - Inform for the quality of the individual studies that were included in the SRs (report results for sequence generation, allocation concealment, blinding, withdrawals, bias etc.) and for the additional included primary studies. | | | | | Supp. 1 |
|  |  | - Quality of evidence. | | | | | Supp. 3 |
| 20. Present meta-bias(es) | 20 | Present results of any assessment of meta-bias(es) (such as publication bias or selective reporting across studies, ROBIS assessment). | | | | | Supp. 4 |
| 21. Synthesis of results | 21a | Summarize and present the main findings of the overview for benefits and harms. If a quantitative synthesis has been conducted, present each summary measure with a confidence interval, prediction interval or a credible interval and measures of heterogeneity or inconsistency. | | | | | Fig 5-6 |
|  | 21b | Give results of any additional analyses (such as sensitivity, subgroup analyses, or meta-regression). | | | | | 9 |
|  | 21c | Report results for adverse events separately for each intervention. | | | | | - |
| **DISCUSSION** | | | | | | |  |
| 22. Summary of evidence | 22 | Provide a concise summary of the main findings with the strength and shortcomings of evidence for each main outcome. | | | | | 9 |
| 23. Limitations | 23a | Discuss limitations of either the overview or included studies (or both) (e.g. different eligibility criteria, limitations of searching reviews, language restrictions, publication and selection bias). | | | | | 10 |
|  | 23b | Report possible limitations of the included reviews related to harms (issues of missing data and information, definitions of harms, rare adverse effects). | | | | | 10 |
| 24. Conclusions | 24a | Provide a general interpretation of the results in coherence with the review findings and present implications for practice; consider the harms equally as carefully as the benefits and in the context of other evidence. | | | | | 10 |
|  | 24b | Present implications for future research. | | | | | 11 |
| **AUTHORSHIP** | | | | | | |  |
| 25. Contributions of authors | 25 | Provide contributions of authors. | | | | | 11 |
| 26. Dual (co-)authorship | 26 | Report about dual (co-)authorship in the limitation or declarations of interest section. | | | | | 11 |
| **FUNDING** | | | | | | |  |
| 27. Funding or other support | 27a | Indicate sources of financial and other support for the OoSRs (direct funding) or for the authors (indirect funding), or report no funding. | | | | | 11 |
|  | 27b | Provide name for the overview funder and/or sponsor, or for the authors’ supporters. | | | | | 11 |
|  | 27c | Describe roles of funder(s), sponsor(s), and/or institution(s), if any, in conducted the OoSRs. | | | | | 11 |

# **Supplementary references**

1. Aapro M, Österborg A, Gascón P, Ludwig H, Beguin Y. Prevalence and management of cancer related anaemia, iron deficiency and the specific role of I.V. iron. Ann Oncol. 2012;23(8):1954-1962.

2. Anand IS, Gupta P. Anemia and Iron Deficiency in Heart Failure: Current Concepts and Emerging Therapies. Circulation. 2018;138(1):80-98.

3. Bailey DM. Physiological implications of altitude training for endurance performance at sea level: A review. Br J Sports Med. 1997;31(3):183-190.

4. Balsalobre-Fernández C, Tejero-González CM, del Campo-Vecino J, Alonso-Curiel D. Hypoxic exposure as a means of increasing sporting performance: Fact or fiction? Rev Int Med y Ciencias la Act Fis y del Deport. 2014;14(53):183-198.

5. Baranauskas MN, Constantini K, Paris HL, Wiggins CC, Schlader ZJ, Chapman RF. Heat Versus Altitude Training for Endurance Performance at Sea Level. Exerc Sport Sci Rev. 2021;49(1):50-58.

6. Bazeley J, Wish JB. The Evolution of Target Hemoglobin Levels in Anemia of Chronic Kidney Disease. Adv Chronic Kidney Dis. 2019;26(4):229-236.

7. Bennett CL. The blue cross blue shield assessment technology review: Summary of findings. Best Pract Res Clin Haematol. 2005;18(3 SPEC. ISS.):423-431.

8. Bukmir L, Fišić M, Diminić-Lisica I, Ljubotina A. [ANEMIA IN CHRONIC KIDNEY DISEASE]. Acta Med Croatica. 2016;70(4-5):217-224.

9. Cacic DL, Hervig T, Seghatchian J. Blood doping: The flip side of transfusion and transfusion alternatives. Transfus Apher Sci. 2013;49(1):90-94.

10. Carson JL, Stanworth SJ, Dennis JA, et al. Transfusion thresholds for guiding red blood cell transfusion. Cochrane Database Syst Rev. 2021;2021(12).

11. Castelli R, Schiavon R, Rossi V, Deliliers GL. Management of anemia in low-risk myelodysplastic syndromes treated with erythropoiesis-stimulating agents newer and older agents. Med Oncol. 2018;35(5):1-10.

12. Chapman RF, Stickford ASL, Lundby C, Levine BD. Timing of return from altitude training for optimal sea level performance. J Appl Physiol. 2014;116(7):837-843.

13. Chen PH, Tsai SY, Hsu JL, Lin HW, Huang SH, Chung KH. Rapid Cycling and lower hemoglobin in elderly bipolar patients with brain atrophy. J Exp Clin Med. 2013;5(3):101-103.

14. Cingam S, Flatow-Trujillo L, Andritsos LA, Yi CA. Ruxolitinib in the treatment of polycythemia vera: An update on health-related quality of life and patient-reported outcomes. J Blood Med. 2019;10:381-390.

15. De Franceschi L, Iolascon A, Taher A, Cappellini MD. Clinical management of iron deficiency anemia in adults: Systemic review on advances in diagnosis and treatment. Eur J Intern Med. 2017;42:16-23.

16. de las Cuevas Allende R, Díaz de Entresotos L, Conde Díez S. Anaemia of chronic diseases: Pathophysiology, diagnosis and treatment. Med Clin (Barc). 2021;156(5):235-242.

17. De Paula P, Niebauer J. Effects of high altitude training on exercise capacity: Fact or myth. Sleep Breath. 2012;16(1):233-239.

18. Dempsey JA, Morgan BJ. Humans in hypoxia: A conspiracy of maladaptation?! Physiology. 2015;30(4):304-316.

19. Eichner ER. Perennial questions: On fatigue, on iron, and on anemia. Curr Sports Med Rep. 2012;11(6):274-275.

20. Ershler WB. Unexplained Anemia in the Elderly. Clin Geriatr Med. 2019;35(3):295-305.

21. Faiss R, Girard O, Millet GP. Advancing hypoxic training in team sports: From intermittent hypoxic training to repeated sprint training in hypoxia. Br J Sports Med. 2013;47(SUPPL. 1):2-9.

22. Ferguson T, Xu Y, Gunasekara R, et al. The cost effectiveness of erythropoietin-stimulating agents for treating anemia in patients on dialysis: A systematic review. Am J Nephrol. 2015;41(2):89-97.

23. Flaherty G, O’Connor R, Johnston N. Altitude training for elite endurance athletes: A review for the travel medicine practitioner. Travel Med Infect Dis. 2016;14(3):200-211.

24. Germing U, Oliva EN, Hiwase D, Almeida A. Treatment of Anemia in Transfusion-Dependent and Non-Transfusion-Dependent Lower-Risk MDS: Current and Emerging Strategies. HemaSphere. 2019;3(6):1-9.

25. Gómez Ramírez S, Remacha Sevilla ÁF, Muñoz Gómez M. Anaemia in the elderly. Med Clin (Barc). 2017;149(11):496-503.

26. Guinn NR, Cooter ML, Weiskopf RB. Lower hemoglobin concentration decreases time to death in severely anemic patients for whom blood transfusion is not an option. J Trauma Acute Care Surg. 2020;88(6):803-808.

27. Hanna RM, Streja E, Kalantar-Zadeh K. Burden of Anemia in Chronic Kidney Disease: Beyond Erythropoietin. Adv Ther. 2021;38(1):52-75.

28. Hébert PC, Hu LQ, Biro GP, Carter A. Review of physiologic mechanisms in response to anemia. Cmaj. 1997;156(11 SUPPL. 1):3-5.

29. Hellström-Lindberg E, Van De Loosdrecht A. Erythropoiesis stimulating agents and other growth factors in low-risk MDS. Best Pract Res Clin Haematol. 2013;26(4):401-410.

30. Jacobs RA, Lundby C, Robach P, Gassmann M. Red blood cell volume and the capacity for exercise at moderate to high altitude. Sport Med. 2012;42(8):643-663.

31. Jing Z, Wei-jie Y, Nan Z, Yi Z, Ling W. Hemoglobin Targets for Chronic Kidney Disease Patients with Anemia: A Systematic Review and Meta-analysis. PLoS One. 2012;7(8).

32. Jones M, Schenkel B, Just J, Fallowfield L. Epoetin alfa improves quality of life in patients with cancer: Results of a metaanalysis. Cancer. 2004;101(8):1720-1732.

33. Kiladjian J-J, Winton EF, Talpaz M, Verstovsek S. Ruxolitinib for the treatment of patients with polycythemia vera. Expert Rev Hematol. 2015;8(4):391-401.

34. Kiliçgedik A, Dündar C, Tigen MK. Kalp yetersizliǧinde anemi. Anadolu Kardiyol Derg. 2012;12(1):65-70.

35. Kimel M, Leidy NK, Mannix S, Dixon J. Does epoetin alfa improve health-related quality of life in chronically ill patients with anemia? Summary of trials of cancer, HIV/AIDS, and chronic kidney disease. Value Heal. 2008;11(1):57-75.

36. Kotecha D, Ngo K, Walters JAE, Manzano L, Palazzuoli A, Flather MD. Erythropoietin as a treatment of anemia in heart failure: Systematic review of randomized trials. Am Heart J. 2011;161(5):822-831.e2.

37. Krantz SB. State of the Art Lecture: Erythropoietin and the anaemia of chronic disease. Nephrol Dial Transplant. 1995;10(supp2):10-17.

38. Krishnan S, Sarda S, Kunzweiler C, et al. Literature Review of Fatigue Scales and Association with Clinically Meaningful Improvements in Outcomes Among Patients With and Without Paroxysmal Nocturnal Hemoglobinuria. Adv Ther. Published online 2022.

39. Leaf DE, Goldfarb DS. Interpretation and review of health-related quality of life data in CKD patients receiving treatment for anemia. Kidney Int. 2009;75(1):15-24.

40. Mies A, Platzbecker U. Increasing the effectiveness of hematopoiesis in myelodysplastic syndromes: erythropoiesis-stimulating agents and transforming growth factor-β superfamily inhibitors. Semin Hematol. 2017;54(3):141-146.

41. Montero D, Lundby C. Regulation of red blood cell volume with exercise training. Compr Physiol. 2019;9(1):149-164.

42. Oliva EN, Platzbecker U, Fenaux P, et al. Targeting health-related quality of life in patients with myelodysplastic syndromes – Current knowledge and lessons to be learned. Blood Rev. 2021;50:100851.

43. Otto JM, Montgomery HE, Richards T. Haemoglobin concentration and mass as determinants of exercise performance and of surgical outcome. Extrem Physiol Med. 2013;2(1):33.

44. Palmer SC, Saglimbene V, Mavridis D, et al. Erythropoiesis-stimulating agents for anaemia in adults with chronic kidney disease: a network meta-analysis. Cochrane Database Syst Rev. Published online December 8, 2014.

45. Pinchon DJ, Stanworth SJ, Dorée C, Brunskill S, Norfolk DR. Quality of life and use of red cell transfusion in patients with myelodysplastic syndromes. A systematic review. Am J Hematol. 2009;84(10):671-677.

46. Płoszczyca K, Langfort J, Czuba M. The Effects of Altitude Training on Erythropoietic Response and Hematological Variables in Adult Athletes: A Narrative Review. Front Physiol. 2018;9.

47. Roubinian N, Carson JL. Red Blood Cell Transfusion Strategies in Adult and Pediatric Patients with Malignancy. Hematol Oncol Clin North Am. 2016;30(3):529-540.

48. Seastone DJ, Gerds AT. Darbepoetin alfa for anemia with myelodysplastic syndrome. Expert Rev Hematol. 2015;8(2):139-146.

49. Shah HH, Fishbane S. Is there an established hemoglobin target range for patients undergoing chronic dialysis? Semin Dial. 2018;31(4):415-419.

50. Stanworth SJ, Killick S, McQuilten ZK, et al. Red cell transfusion in outpatients with myelodysplastic syndromes: a feasibility and exploratory randomised trial. Br J Haematol. 2020;189(2):279-290.

51. Silverberg DS, Wexler D, Iaina A, Schwartz D. Anaemia management in cardio renal disease. J Ren Care. 2010;36 Suppl 1(C):86-96.

52. Staibano P, Perelman I, Lombardi J, et al. Patient-Centered Outcomes in the Management of Anemia: A Scoping Review. Transfus Med Rev. 2019;33(1):7-11.

53. Stein BL, Moliterno AR, Tiu R V. Polycythemia vera disease burden: contributing factors, impact on quality of life, and emerging treatment options. Ann Hematol. 2014;93(12):1965-1976.

54. Stickel F, Helbling B, Heim M, et al. Critical review of the use of erythropoietin in the treatment of anaemia during therapy for chronic hepatitis C. J Viral Hepat. 2012;19(2):77-87.

55. Strauss W, Auerbach M. Health-related quality of life in patients with iron deficiency anemia: impact of treatment with intravenous iron. Patient Relat Outcome Meas. 2018;Volume 9:285-298.

56. Strzała M, Ostrowski A, Szyguła Z. Altitude training and its influence on physical endurance in swimmers. J Hum Kinet. 2011;28(1):91-105.

57. Wagner PD, Simonson TS, Wei G, et al. Sea-level haemoglobin concentration is associated with greater exercise capacity in Tibetan males at 4200 m. Exp Physiol. 2015;100(11):1256-1262.

58. Wehrlin JP, Marti B, Hallén J. Hemoglobin mass and aerobic performance at moderate altitude in elite athletes. Adv Exp Med Biol. 2016;903:357-374.

59. Wilde L, Pan J. Restrictive Versus Liberal Transfusion Strategies in Myelodysplastic Syndrome and Beyond. Clin Lymphoma, Myeloma Leuk. 2019;19(12):758-762.

60. Zelenkova I, Zotkin S, Korneev P, Koprov S, Grushin A. Comprehensive overview of hemoglobin mass and blood volume in elite athletes across a wide range of different sporting disciplines. J Sports Med Phys Fitness. 2019;59(2).

61. Collister D, Komenda P, Hiebert B, et al. The effect of erythropoietin-stimulating agents on health-related quality of life in anemia of chronic kidney disease: A systematic review and meta-analysis. Ann Intern Med. 2016;164(7):472-478.

62. Heuberger JAAC, Cohen Tervaert JM, Schepers FML, et al. Erythropoietin doping in cycling: Lack of evidence for efficacy and a negative risk-benefit. Br J Clin Pharmacol. 2013;75(6):1406-1421.

63. Sgrò P, Sansone M, Sansone A, Romanelli F, Di Luigi L. Effects of erythropoietin abuse on exercise performance. Phys Sportsmed. 2018;46(1):105-115.
